# *Drosophila melanogaster* establishes a species-specific mutualistic interaction with stable gut-colonizing bacteria

**DOI:** 10.1371/journal.pbio.2005710

**Published:** 2018-07-05

**Authors:** Inês S. Pais, Rita S. Valente, Marta Sporniak, Luis Teixeira

**Affiliations:** 1 Instituto Gulbenkian de Ciência, Oeiras, Portugal; 2 Faculdade de Medicina da Universidade de Lisboa, Lisboa, Portugal; University of Texas at Austin, United States of America

## Abstract

Animals live together with diverse bacteria that can impact their biology. In *Drosophila melanogaster*, gut-associated bacterial communities are relatively simple in composition but also have a strong impact on host development and physiology. It is generally assumed that gut bacteria in *D*. *melanogaster* are transient and their constant ingestion with food is required to maintain their presence in the gut. Here, we identify bacterial species from wild-caught *D*. *melanogaster* that stably associate with the host independently of continuous inoculation. Moreover, we show that specific *Acetobacter* wild isolates can proliferate in the gut. We further demonstrate that the interaction between *D*. *melanogaster* and the wild isolated *Acetobacter thailandicus* is mutually beneficial and that the stability of the gut association is key to this mutualism. The stable population in the gut of *D*. *melanogaster* allows continuous bacterial spreading into the environment, which is advantageous to the bacterium itself. The bacterial dissemination is in turn advantageous to the host because the next generation of flies develops in the presence of this particularly beneficial bacterium. *A*. *thailandicus* leads to a faster host development and higher fertility of emerging adults when compared to other bacteria isolated from wild-caught flies. Furthermore, *A*. *thailandicus* is sufficient and advantageous when *D*. *melanogaster* develops in axenic or freshly collected figs, respectively. This isolate of *A*. *thailandicus* colonizes several genotypes of *D*. *melanogaster* but not the closely related *D*. *simulans*, indicating that the stable association is host specific. This work establishes a new conceptual model to understand *D*. *melanogaster*–gut microbiota interactions in an ecological context; stable interactions can be mutualistic through microbial farming, a common strategy in insects. Moreover, these results develop the use of *D*. *melanogaster* as a model to study gut microbiota proliferation and colonization.

## Author summary

Animals, including humans, live together with complex bacterial communities in their gut that influence their physiology and health. The fruit fly *Drosophila melanogaster* is an excellent model organism to study host–microbe interactions and harbors a relatively simple gut bacterial community. The dominating hypothesis in the field is that gut bacteria in *D*. *melanogaster* are unstable and their constant ingestion with food is required to maintain their levels in the gut. Here, however, we show in *D*. *melanogaster* collected from a natural population, that stable gut bacteria do exist. We isolated specific species that can proliferate in the gut and form a stable association that is beneficial for both the bacteria and the flies. For the bacteria, they benefit from being constantly disseminated by the flies as they move around. For the flies, this is a form of farming, as the next generation of flies benefits from the association with these particular bacteria during development. Flies associated with these bacteria during the larval stages become adults faster and are more fertile than if they develop with other bacteria encountered in nature. Our findings show that *D*. *melanogaster* has stable colonizing bacteria in the gut, which are important for host development, establishing a new framework to study host–gut bacteria interactions.

## Introduction

Animals live with microbial communities that have a strong impact on their physiology, including their development, nutrition, immunity, and behavior [1]. These effects may be partially explained by adaptation of animals to the ubiquitous presence of microbes and integration of this cue in their developmental and physiological programs. However, association with specific microbes may increase their fitness in their environment or provide the capacity to explore new niches. For instance, many endosymbionts in insects provide essential metabolites, allowing hosts to explore food sources deficient in some nutrients, such as plant sap and blood [2–6].

A primary organ for animal–microbe interactions is the gut, which is an interface between the external environment and the animal body. The gut microbiota can be very complex and comprised of up to 1,000 different bacterial species, as in humans [7]. Its composition varies to different degrees between and within host species. Moreover, even within the same host it can be very dynamic and fluctuate with host age and health, diet, and other environmental conditions [8–11]. Understanding the composition of the gut microbiota, which factors regulate it, and how these interactions impact both the host and the microbes are, therefore, major research questions.

*Drosophila melanogaster* has been used as model system to study host interaction with gut bacteria [12,13]. Besides the host genetics, it has the advantages of having a simpler bacterial community, when compared with mammals, and of being relatively simple to produce axenic and gnotobiotic animals. *D*. *melanogaster* raised in axenic conditions have a delayed development and are not viable under certain nutritional conditions, and bacteria can rescue these developmental problems [14–16]. Bacteria also affect the fly life span, gut homeostasis, interaction with pathogens, and behavior [17–23]. All these phenotypes demonstrate the importance of bacteria to this host and the need to understand these interactions for a comprehensive view of *D*. *melanogaster* biology.

Despite the recognized importance of gut-associated bacteria to *D*. *melanogaster*, what constitutes its gut microbiota is still an open question. Laboratory *D*. *melanogaster* is associated with few bacterial species, which belong mainly to *Acetobacter* and *Lactobacillus* genera [20,22,24–27]. This contrasts with data from flies sampled in their natural environment, which have a more diverse population of bacteria. In addition to *Acetobacter* and *Lactobacillus*, they are also enriched in bacteria from other families and genera [25,28]. Because *D*. *melanogaster* feeds on fermenting and rotten fruits containing many microbes, it is, however, difficult to understand which of the bacteria are colonizing the host gut and which are transiently passing with the food. Likewise, a similar problem is present in laboratory conditions, where flies live in a relatively closed environment. The bacteria found in their gut could simply correspond to food growing bacteria ingested by the flies. This hypothesis is supported by the fact that frequent transfer of adult flies to clean food vials strongly reduces their gut bacterial loads [20,27]. Consequently, the current working model is that the gut-associated bacteria in *D*. *melanogaster* are environmentally acquired and do not constitute bona fide gut symbionts.

Most functional studies in *D*. *melanogaster*, however, have been performed with bacterial isolates from lab stocks. The properties of bacterial isolates from wild-caught *D*. *melanogaster* could differ. Bacteria found in the gut of some other *Drosophila* species differ from the bacteria present in their food source, suggesting that they can be gut symbionts [29,30] and raising the possibility of these also existing in *D*. *melanogaster*. Moreover, a recent study compared the ability of different *Lactobacillus plantarum* strains to colonize the gut and found that one wild strain was able to colonize flies more frequently than strains isolated from laboratory flies [31]. Therefore, it is possible that natural populations of *D*. *melanogaster* have stable colonizing bacterial communities in their guts.

Here, we analyzed bacterial isolates from the gut of wild-caught *D*. *melanogaster* and compared it to bacteria from lab stocks. Using a protocol that avoids reinfection of flies with bacteria growing on the food, we identified bacterial species that are stably associated with the gut of wild *D*. *melanogaster*. Moreover, these isolates can stably associate and proliferate in the gut of lab flies. We further analyze the specificity of these interactions and fitness advantage of stable associations. Our results lead to the identification of gut symbionts in *D*. *melanogaster* and demonstrate fitness advantages for both partners in an ecological context.

## Results

### Wild-caught flies have stable gut-colonizing bacteria

In order to analyze the diversity and stability of gut bacteria in *D*. *melanogaster*, we used culture-dependent techniques. We plated single gut homogenates in five different culture media. This approach allowed us to determine the absolute number of bacteria present in each gut and isolate bacteria for follow-up experiments.

**Fig 1:**
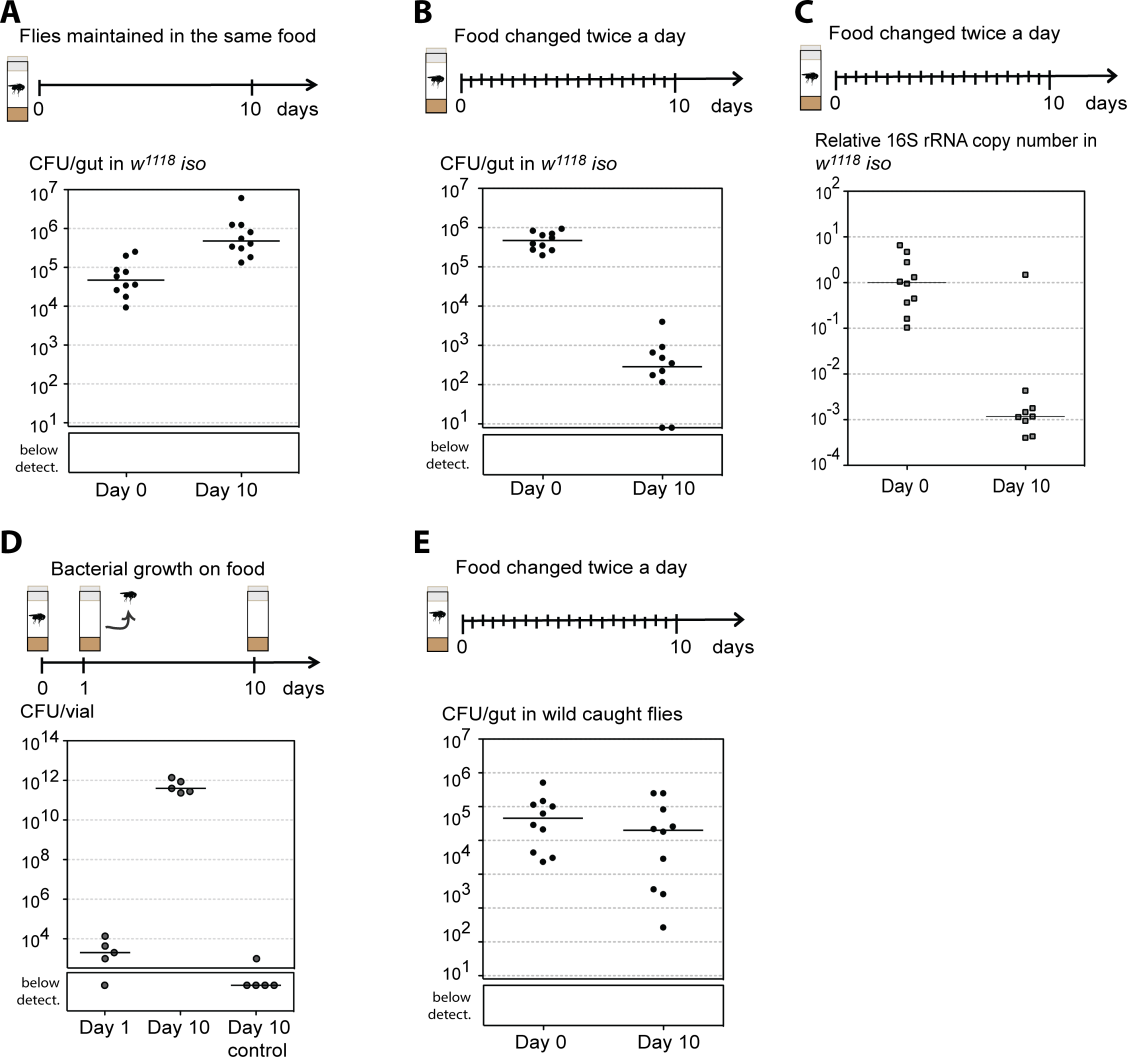
Wild-caught *Drosophila melanogaster* have a stable gut microbiota. Single 3–6-day-old *w*^*1118*^ *iso* males were kept in the same vial during 10 days (A) or exposed to a stability protocol by being passed to new vials twice a day (B, C). (A, B) Ten individuals were analyzed each day and total number of CFUs per gut was determined by bacterial plating. Bacterial levels between Day 0 and Day 10 increase in (A) and decrease in (B) (lmm, *p* < 0.001 for both). Supporting data can be found in S1 and S2 Data. (C) Relative amount of 16S rRNA bacterial gene was measured by qPCR in 10 individual guts from each day, using the host gene *Rpl32* as a reference gene. The relative amount of 16S rRNA gene decreases between days (lmm, *p* < 0.001). Supporting data can be found in S3 Data. (D) Single 3–6-day-old *w*^*1118*^ *iso* males were placed in food vials for 24 hours and then discarded. Bacterial levels on the food were determined at this point (Day 1) and after incubating the vials for a further 9 days (Day 10). Bacterial levels were also assessed in control vials, not exposed to flies (Day 10 control). Five vials were analyzed for each condition and total number of CFUs per vial was determined by bacterial plating. Bacterial levels increase between Day 1 and Day 10 (lm, *p* < 0.001). Supporting data can be found in S4 Data. (E) Bacterial levels from wild-caught flies at the day of collection (Day 0) and after 10 days of the stability protocol (Day 10). Ten individuals were analyzed for each day and total number of CFUs per gut was determined by plating. Bacterial levels on the flies significantly decrease with time (lmm, *p* = 0.004). Supporting data can be found in S5 Data. (A–E) Each dot represents an individual gut or vial and lines represent medians. Statistical analyses were performed together with replicate experiments shown in S1 Fig. CFU, colony-forming unit; lm, linear model; lmm, linear mixed model; *w*^*1118*^ *iso*, *w*^*1118*^ DrosDel isogenic strain; qPCR, quantitative PCR.

We started by analyzing levels of bacteria in the gut of flies from our standard laboratory stock *w*^*1118*^ DrosDel isogenic strain (*w*^*1118*^ *iso*) [32,33]. We assessed these levels in young conventionally raised flies (Day 0) and after these flies were maintained singly for 10 days either in the same vial or passed to a new vial twice a day (similarly to the protocol in [20]). The latter protocol was designed to decrease the probability of flies getting reinfected with their own bacteria or bacteria growing on fly food and, therefore, allowed us to test if there was a resident gut bacterial microbiota in this *D*. *melanogaster* lab stock (stability assay). In flies kept in the same vial for 10 days, bacterial levels in the gut increased approximately 17-fold (Fig 1A and S1A Fig, linear mixed model [lmm] fit, *p* < 0.001). In contrast, flies that were passed twice a day had an approximately 2,200-fold decrease in their gut bacterial levels (Fig 1B and S1A Fig, lmm, *p* < 0.001). A sharp decrease in bacterial loads was confirmed by quantitative PCR (qPCR), a culture-independent method, using universal primers for the 16S rRNA gene (Fig 1C and S1B Fig, lmm, *p* < 0.001). These results show that bacterial levels in the gut of these flies are dependent on fly husbandry and suggest that these bacteria are transient, similarly to what was previously shown with a different laboratory stock [20]. Because these bacteria are associated with the lab stock, and bacterial loads in the gut of these flies actually increase over time if they are kept in the same vials for 10 days, we tested their growth on fly food (Fig 1D). We placed single flies per vial (Day 0), discarded them after 24 hours (Day 1), and kept the vials for a further nine days (Day 10). Bacterial levels on the surface of the fly food increased 7.6×10^8^-fold from Day 1 to Day 10, clearly showing their capacity to grow on fly food (Fig 1D, linear model [lm], *p* < 0.001). Therefore, the bacteria associated with this lab stock grow on the fly food and are only transiently associated with the gut of adult flies.

We next asked if we could find stable bacteria in the gut of *D*. *melanogaster* collected from natural populations. We captured *D*. *melanogaster* from a population growing on fallen figs and quantified their gut bacterial levels at the time of collection (Day 0) and 10 days after, using the same stability assay designed to avoid reinfection (Day 10) (Fig 1E, S1C and S1D Fig). Although there is a statistically significant change in the bacterial levels in the gut with time (lmm, *p* = 0.004), the bacterial levels only decreased 4.8-fold in 10 days. Moreover, at Day 10, wild flies maintained 2.9×10^4^ colony-forming units (CFUs) per gut, while *w*^*1118*^ *iso* flies only had 100 CFUs per gut. Also, even after 20 days of this protocol, wild flies still maintained approximately 6.1×10^3^ CFU per gut (S1D Fig), showing long-term stability of their microbiota. These results show that wild flies carry bacteria that are stably associated with their gut.

**Fig 2:**
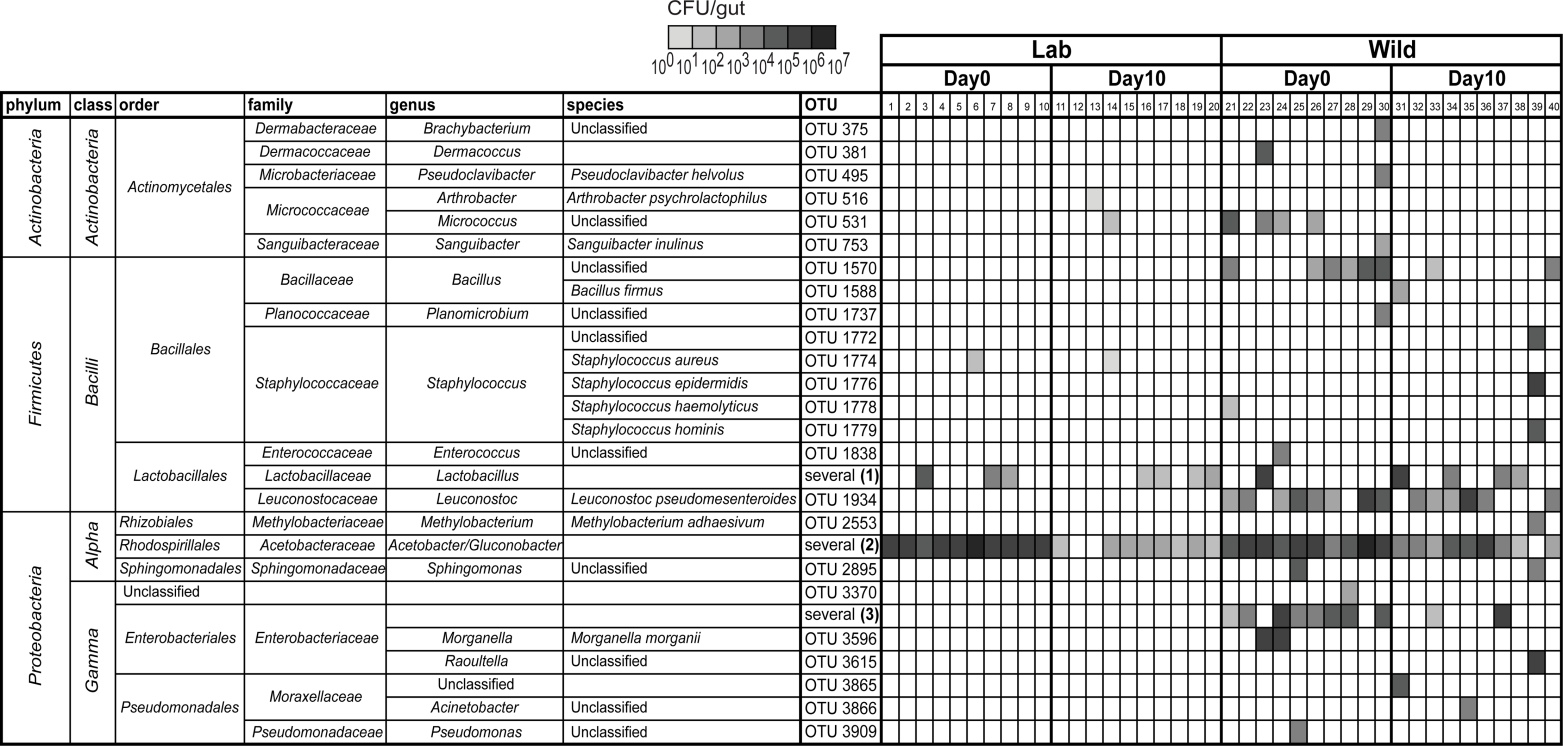
Higher diversity of gut bacterial communities in wild-caught *Drosophila melanogaster*. Bacterial OTUs present in the gut of laboratory (1–20) and wild-caught (21–40) flies before (Day 0) and after being exposed to the stability protocol (Day 10). Gut homogenates from flies represented in Fig 1B and 1E were plated in different culture media, and representative colonies of each morphological type were sequenced. Each column represents one individual gut. Bacterial levels are represented on a gray scale from 10^0^ to 10^7^ CFUs per gut. Colonies of different *Lactobacillus*, Acetobacteriaceae, or Enterobactereaceae were not possible to distinguish by morphological type and are grouped together. The presence of *Lactobacillus* species and *Leuconostoc pseudomesenteroides* in wild-caught flies is not independent (Pearson’s chi-squared test, *p* = 0.014). Frequencies of the different OTUs in these groups are represented on Fig 3B, 3D, 3F and 3H and S3B Fig. Supporting data can be found in S6 Data. CFU, colony-forming unit; OTU, operational taxonomic unit.

**Fig 3:**
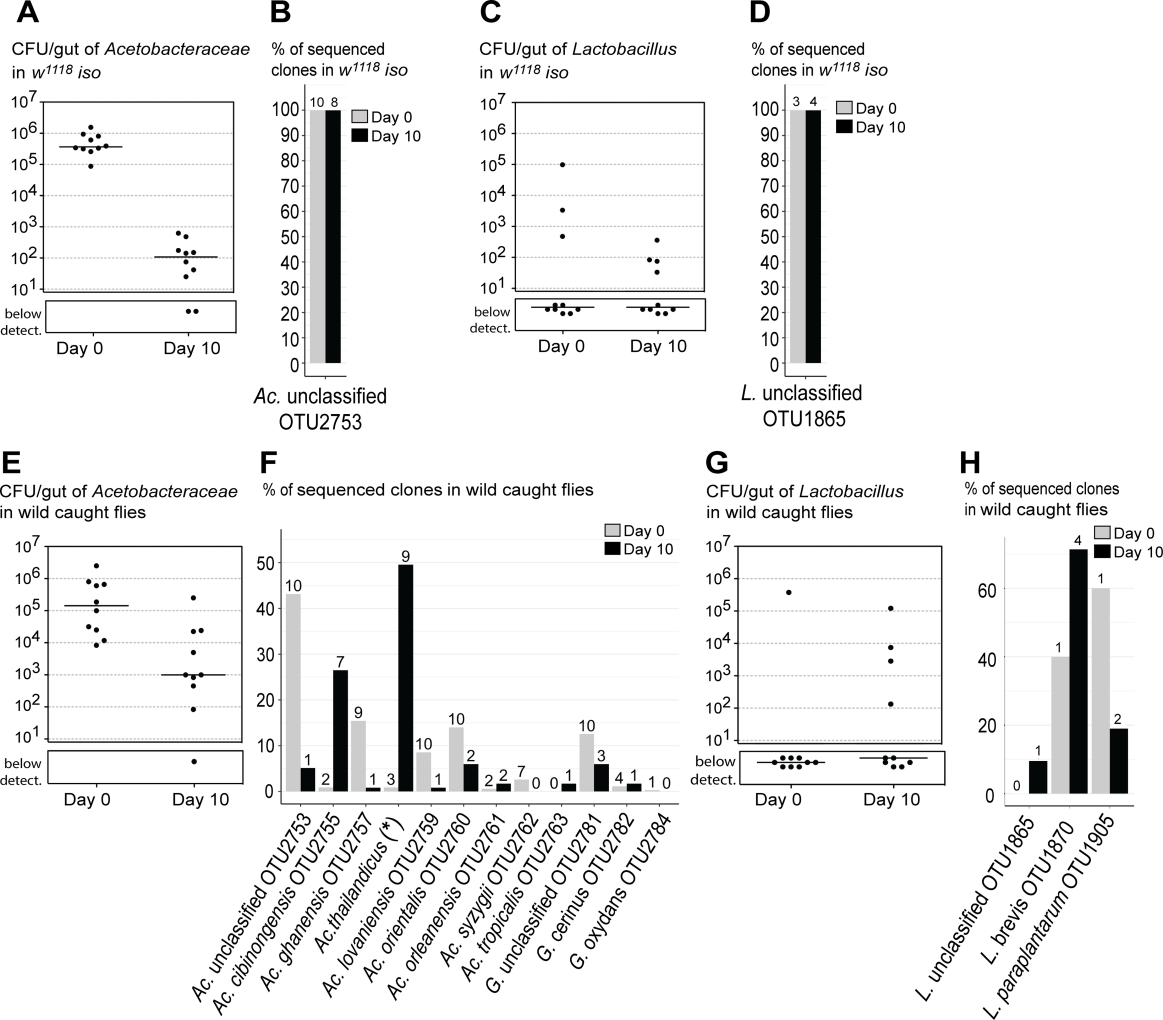
Wild-caught flies maintain particular *Acetobacter* species in the gut. Total levels of Acetobactereaceae (A, E) and *Lactobacillus* (C, G) in laboratory *w*^*1118*^ *iso* (A, C) and in wild-caught flies (E, G) before (Day 0) and after 10 days of the stability protocol (Day 10). Each dot represents one individual gut and lines represent medians. Levels of Acetobactereaceae decrease between days in both types of flies (lm, *p* ≤ 0.002 for both). Changes in levels of *Lactobacillus* are not significant in both (lm, *p* ≤ 0.302). Frequencies of sequenced colonies of Acetobactereaceae (B, F) and *Lactobacillus* (D, H) in *w*^*1118*^ *iso* (B, D) and in wild-caught flies (F, H). Numbers on the top of the bars correspond to the number of flies carrying each OTU, from a total of 10 flies (B, D, F, H). *Acetobacter thailandicus was initially identified as *A*. *indonesiensis* OTU2758 based on partial sequence of 16S rRNA gene; see Materials and methods and S1 Text. Supporting data can be found in S6 Data. *Ac*., *Acetobacter*; CFU, colony-forming unit; *G*., *Gluconobacter*; *L*., *Lactobacillus*; lm, linear model; OTU, operational taxonomic unit; *w*^*1118*^ *iso*, *w*^*1118*^ DrosDel isogenic strain.

In order to identify and isolate the bacteria that can stably interact with the gut of *D*. *melanogaster*, we analyzed the bacterial composition of the cultured gut extracts of *w*^*1118*^ *iso* and wild flies represented in Fig 1B and 1E. For each fly gut homogenate, in each of the five media, we distinguished colonies by morphology, determined CFUs per gut of each morphological type, and isolated two colonies of each morphological type. For each isolate, we sequenced by Sanger a fragment of the 16S rRNA gene, which included the V2 to V4 hypervariable regions. After sequencing, we classified morphological types into operational taxonomic units (OTUs), based on Greengenes alignment tool and database [34], and determined the number of CFUs of each OTU in each fly gut (Fig 2). In general, we could assign each morphological type to one OTU. However, in samples from wild flies we could not distinguish by morphology the colonies of different *Lactobacillus* species, different Acetobacteraceae (genera *Acetobacter* and *Gluconobacter*) species, and several genera of Enterobacteriaceae. We therefore calculated CFUs per fly for each of these groups of bacteria and not individual OTUs (Fig 2). The frequencies of the different OTUs belonging to these groups, in the different conditions, are shown in Fig 3B, 3D, 3F and 3H and S3 Fig.

Laboratory flies were mainly found associated with two OTUs, *Acetobacter* OTU2753 and *Lactobacillus* OTU1865, and accumulation curves indicate that we sampled most of the diversity present in these flies (S2 Fig). On Day 0, all the flies were associated with high levels of *Acetobacter* OTU2753 (Fig 3A and 3B), while *Lactobacillus* OTU1865 was only present in some individuals (Fig 3C and 3D). After 10 days of the stability assay, *Acetobacter* levels decreased (lm, *p* = 0.001), while *Lactobacillus* levels are not significantly different (*p* = 0.635) (Fig 3A and 3C). Importantly, when we analyzed the bacterial species that were capable of growing on fly food in Fig 1D, we found these two same OTUs, with *Acetobacter* OTU2753 being the most abundant. Altogether, these results show that this *D*. *melanogaster* laboratory stock has very low bacterial diversity, as previously reported in other stocks [25,26,28].

In contrast, wild-caught flies were associated with a higher diversity of bacterial species (Fig 2, Fig 3F and 3H and S3B Fig). From each gut of wild flies we isolated 9–16 different OTUs at Day 0, and 3–14 different OTUs at Day 10. In total, we isolated 35 and 31 different OTUs at Day 0 and Day 10, respectively (S2 Fig). Moreover, it seems that we are not close to saturation with these samples and that further sampling would allow the identification of more OTUs associated with the gut of *D*. *melanogaster* from this wild population (S2 Fig).

The individual characterization of bacterial species present in each gut allowed us to discriminate between OTUs that were only present in one or few individuals, albeit at higher levels, and OTUs associated with most individuals. At the day of collection (Day 0), 50% or more of the flies had in their gut *Bacillus* OTU1570, *Leuconostoc pseudomesenteroides* OTU1934, *Acetobacter* OTU2753, *Acetobacter ghanensis* OTU2757, *A*. *lovaniensis* OTU2759, *A*. *orientalis* OTU2760, *A*. *syzygii* OTU2762, *Gluconobacter* OTU2781, Enterobactereaceae OTU3529, *Tatumella* OTU3635, and *Kluyvera ascorbata* OTU3643 (Fig 2, Fig 3F and S3B Fig). Ten days after the stability assay, only a few bacteria remained associated with the gut of most individuals. One of these bacteria was *L*. *pseudomesenteroides*, which was present in 6 out of 10 flies and did not show a significant reduction in levels between Day 0 and Day 10 (Fig 2, S4 Fig, lm, *p* = 0.372). Bacteria from the Acetobacteraceae family also remained associated with the gut of most wild flies at an estimated 1.3×10^3^ CFU per gut at Day 10, despite a significant reduction of approximately 100-fold in their levels between Day 0 and Day 10 (lm, *p* = 0.002) (Fig 2, Fig 3E). However, the frequencies of different OTUs of Acetobacteraceae changed significantly between Day 0 and Day 10 (Fig 3F, Pearson’s chi-squared with Monte Carlo simulation, *p* < 0.001). At Day 10, all the OTUs that were dominant at Day 0 became present at lower frequencies, and *A*. *cibinongensis* OTU2755 and *A*. *thailandicus* became the dominant bacteria (Fig 3F). These two bacteria were present in at least 7 and 9 individuals out of 10, respectively, and together represented 76% of the sequenced colonies. Overall, this analysis identified three species that seem to be stably associated with the gut of wild flies in this population: *L*. *pseudomesenteroides*, *A*. *cibinongensis*, and *A*. *thailandicus*.

### *A*. *thailandicus* and *A*. *cibinongensis* stably associate with the gut of *D*. *melanogaster*

To study the interaction of these bacteria with *D*. *melanogaster*, we generated stocks of *w*^*1118*^ *iso* flies monoassociated with each of these bacteria and we tested their persistence using the stability assay. In agreement with our previous observations, the laboratory isolate of *Acetobacter* OTU2753 did not persist in the gut and disappeared from the majority of the flies (lmm, *p* < 0.001) (S5A and S5E Fig). On the other hand, the wild isolates of *A*. *cibinongensis*, *A*. *thailandicus*, and *L*. *pseudomesenteroides* persisted in the gut of flies until Day 10, showing a more stable association with the host (S5B–S5D and S5F–S5H Fig). *L*. *pseudomesenteroides* levels did not significantly change with treatment (*p* = 0.96) and, although *A*. *cibinongensis* and *A*. *thailandicus* levels significantly decreased in the 10 days (*p* < 0.001 for both), both remained in the gut at approximately 100 and 3,800 CFUs, respectively.

**Fig 4:**
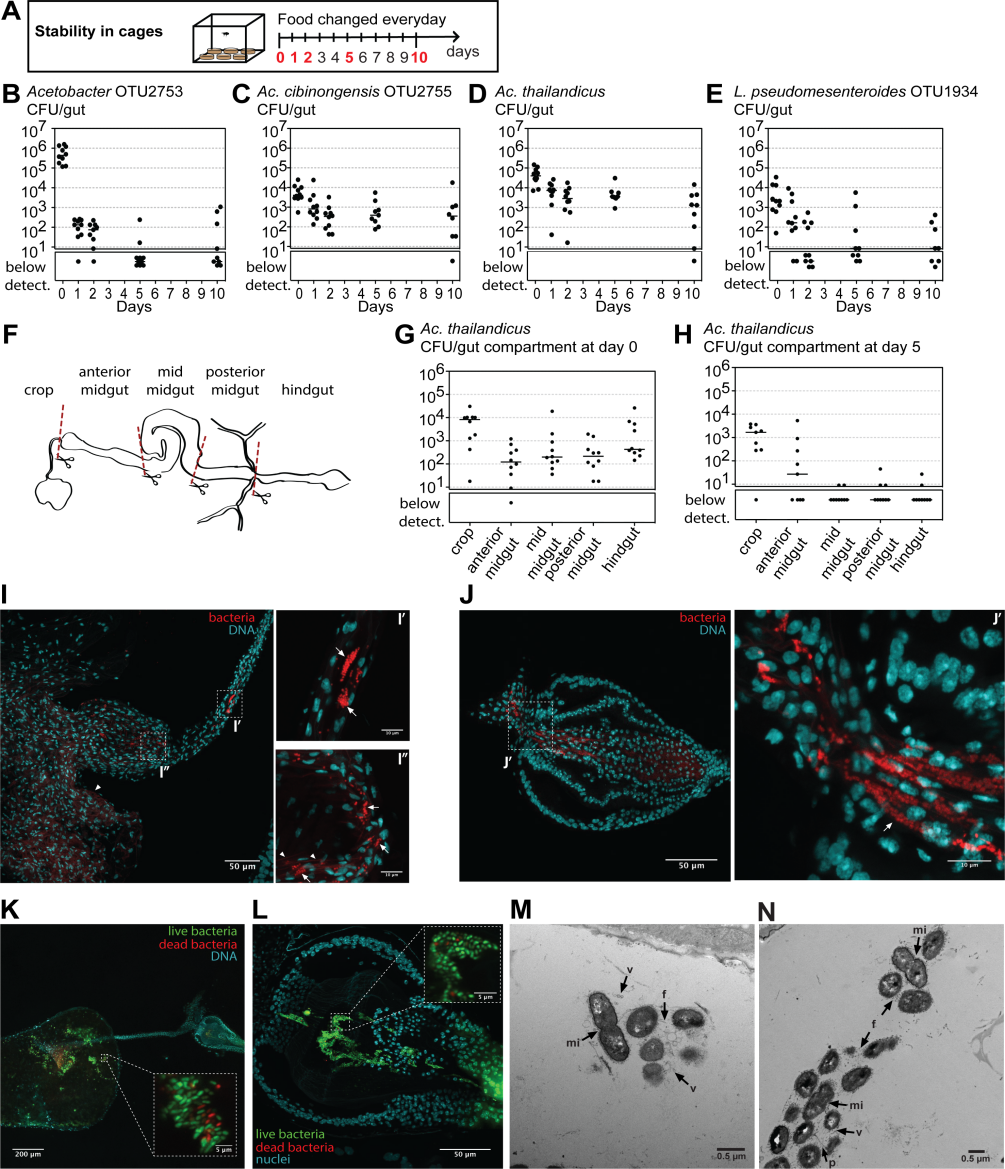
*Acetobacter thailandicus* stably persists in the foregut of *Drosophila melanogaster*. (A–E) Stability of different bacteria in monoassociation. Single 3–6-day-old *w*^*1118*^ *iso* males from monoassociated stocks, with *Acetobacter* OTU2753 (B), *A*. *cibinongensis* (C), *A*. *thailandicus*, (D) or *Leuconostoc pseudomesenteroides* (E), were exposed to the stability protocol in cages, as shown in the scheme (A). Number of CFUs in individual guts was assessed by plating at Days 0, 1, 2, 5, and 10 of the protocol. Stability of different bacteria was analyzed by fitting the data to an exponential decay model represented in S5I Fig. Supporting data can be found in S7 Data. (F–N) Localization of *A*. *thailandicus* in the gut. (G–H) Number of CFUs in each gut compartment from *w*^*1118*^ *iso* males monoassociated with *A*. *thailandicus* before (G) and after (H) 5 days of the stability protocol. Guts were dissected and cut according to the scheme in (F). Each dot represents one gut or one gut fragment and lines represent medians; statistical analyses were performed together with replicate experiments shown in S6 Fig. Supporting data can be found in S8 Data. (I, J) Fluorescent in situ hybridization with Cy3 labeled Bacteria 16S rRNA universal probe EUB338 in the gut of males monoassociated with *A*. *thailandicus*, after 5 days of the stability protocol. *A*. *thailandicus* persists in the crop duct (I′), the crop (I″), and the proventriculus (J and J′). In the crop, *A*. *thailandicus* cells are observed close to the chitin (I″). Chitin autofluorescence is indicated by white arrowheads and bacteria by white arrows. (K, L) Live/dead staining in the crop (K) and proventriculus (L) of males monoassociated with *A*. *thailandicus* 9 days after the stability protocol. Live bacteria stained with SYTO9 (green) and dead bacteria with propidium iodide (red). (I–L) DNA was stained with Hoechst. (M–N) Aggregates of *A*. *thailandicus* observed by transmission electron microscopy in the lumen of the proventriculus. Some cells present membrane invaginations (mi) and seem to be dividing. Cells appear to be attached to each other by external appendages such as fimbriae (f). A pili-like structure is also present (p). Extracellular vesicles are found between cells (v). Scale bar corresponds to 200 μm in (K), 50 μm in (I, J, L), 10 μm in (I′, I″, J′), 5 μm in the zoom in panels of (K, L), and 0.5 μm in (M, N). CFU, colony-forming unit; Cy3, cyanine 3 dye; mi, membrane invagination; OTU, operational taxonomic unit; p, pili; v, vesicles; *w*^*1118*^ *iso*, *w*^*1118*^ DrosDel isogenic strain.

To better assess the bacterial dynamics within the gut, we developed a stricter protocol to avoid reinfection. We maintained single flies in cages with a larger food surface (382 cm^2^ compared with 3.8 cm^2^ in vials), which was changed daily (Fig 4A). We assessed gut bacterial levels at the beginning of the experiment and after 1, 2, 5, and 10 days of this treatment. In accordance with previous data, *Acetobacter* OTU2753 levels rapidly decreased and most flies had no detectable bacteria in their gut after 5 days of treatment (Fig 4B). *A*. *cibinongensis* and *A*. *thailandicus* also presented an initial decrease in bacterial levels in the gut, but these seemed to stabilize after 2 days of treatment, confirming their stability in the gut (Fig 4C and 4D). However, and contrary to what was observed in vials, *L*. *pseudomesenteroides* was not stable when the protocol was performed in cages (Fig 4E). After 2 days, approximately 50% of flies lost *L*. *pseudomesenteroides* from their gut. An independent experimental replicate with data from only Day 0 and Day 5 showed similar results for all bacteria (S5E–S5H Fig).

We compared the dynamics of the gut levels of the four bacteria by fitting the data of Fig 4B–4E to an exponential decay model (S5I Fig). This model estimates the exponential decay rate, which corresponds to the rate of bacterial loss from the gut, and an asymptote that corresponds to the levels at which the bacteria tend to stabilize after this loss. The simplest model that explains the data has the same estimate for the exponential decay rate for all the bacteria. There are, however, significant differences between the asymptotes of all the bacteria (contrasts between nonlinear least-squares estimates, *p* < 0.014), except between *Acetobacter* OTU2753 and *L*. *pseudomesenteroides* (*p =* 0.116). Overall, an interpretation of this fit is that, in all cases, most of the bacterial population is in an unstable compartment at the beginning of the experiment, from where they tend to disappear, with similar dynamics. However, *A*. *cibinongensis* and *A*. *thailandicus* are also present in a stable compartment at levels that correspond to the calculated asymptotes (approximately 300 and 1,300 CFU per gut, respectively).

### The stable *A*. *thailandicus* population resides in the foregut of *D*. *melanogaster*

In order to identify in which gut region bacteria could be stably associated with the host, we analyzed *A*. *thailandicus* levels present in different gut regions before (Day 0) and after 5 days of the stability protocol in cages (Day 5) (Fig 4F). At Day 0, *A*. *thailandicus* was distributed along the gut, being present at lower levels in the midgut, compared with crop and hindgut (Fig 4G and S6A Fig). After 5 days, bacteria were found in two anterior gut sections, one comprising the crop and the other comprising the anterior midgut and the proventriculus (Fig 4H and S6B Fig). Fluorescent in situ hybridization (FISH) with a universal probe for 16S rRNA confirmed and refined these results. At Day 0, *A*. *thailandicus* is present in all gut compartments, but after 5 days of the stability protocol, the bacteria persist almost exclusively in the foregut of *D*. *melanogaster*. *A*. *thailandicus* was consistently found in the crop (mostly at the anterior part of the crop), the crop duct, and the proventriculus in both males and females (Fig 4I and 4J, S6C–S6D Fig, S7A–S7C Fig, S8 Fig). Interestingly, the bacteria seem to be aggregated in clusters (Fig 4I′, 4I″ and 4J′). In the crop these bacteria are mainly found at the periphery, not the lumen, and close to chitin (Fig 4I and 4I″). Bacteria were rarely found in the midgut and rectum and, when present, are also in small clusters (S7D and S7E Fig).

We performed a Live/Dead staining on the stable *A*. *thailandicus* population in order to analyze the proportion of live bacteria in these clusters. Most of the bacteria in the crop and proventriculus are alive (Fig 4K and 4L).

Electron microscopy in the crop and proventriculus confirmed that *A*. *thailandicus* is present in clusters and bacterial cells seem to be connected by external appendages, probably fimbriae (Fig 4M and 4N and S9 Fig) [35]. In the crop, the clusters of bacterial cells were found within folds and, therefore, are close to chitin (S9A and S9B Fig). Importantly, several bacteria presented membrane invaginations compatible with cell division in gram-negative bacteria (Fig 4M, S9H Fig) [36], indicating that these cells are proliferating. Interestingly, we also observed extracellular vesicles associated with the bacterial clusters, and structures resembling pili between bacteria (Fig 4M and 4N, S9H and S9I Fig).

Altogether, these analyses show that the stable population of *A*. *thailandicus* persists in aggregates in the crop and proventriculus of *D*. *melanogaster*. Their niche has a clear boundary at the end of the proventriculus because they are rarely found from the beginning of the midgut on.

### *A*. *thailandicus* and *A*. *cibinongensis* proliferate in the gut of *D*. *melanogaster*

**Fig 5:**
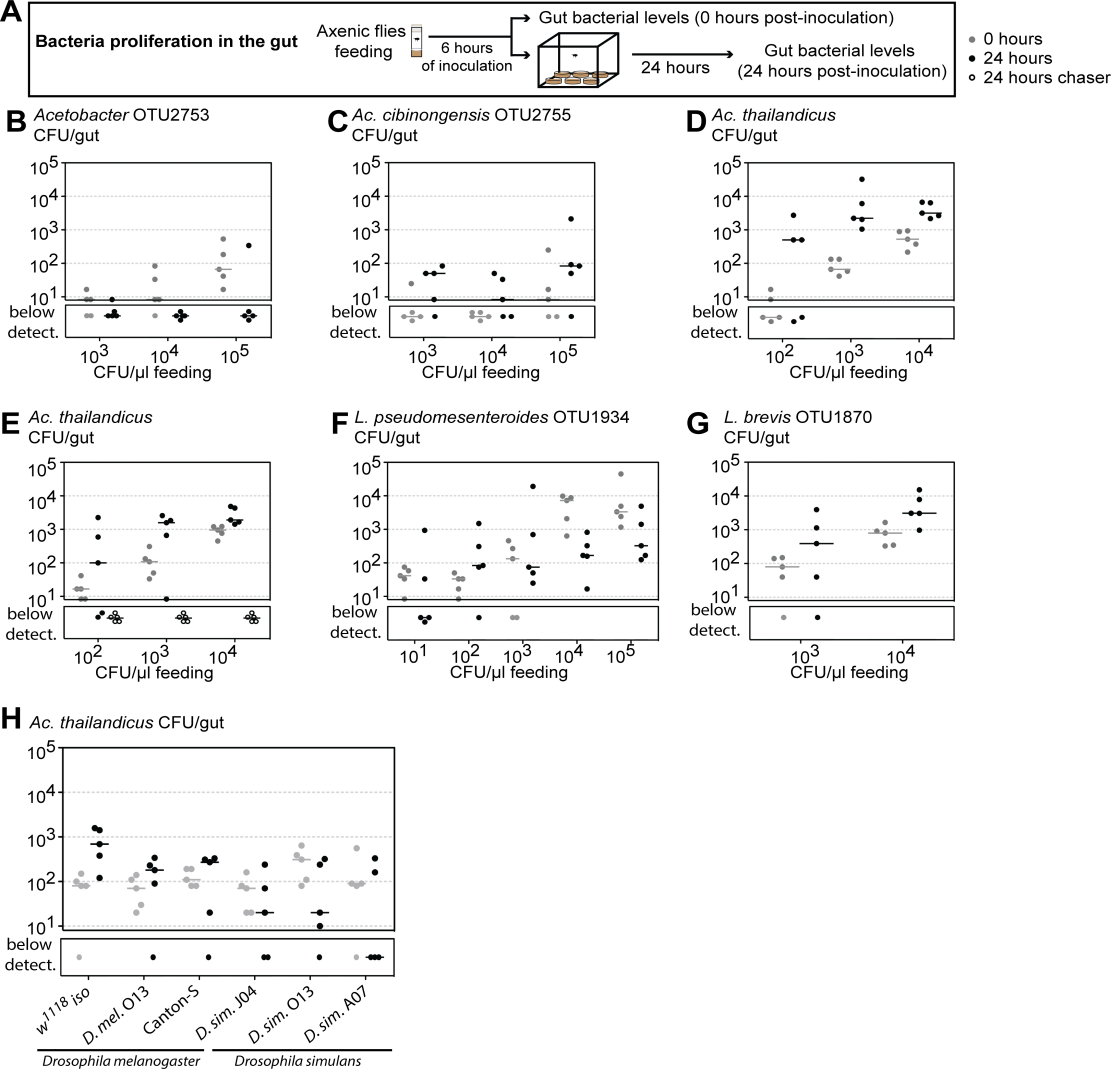
*Acetobacter thailandicus* and *A*. *cibinongensis* proliferate in the gut of *Drosophila melanogaster*. (A–G) Proliferation of different bacteria in the gut of *D*. *melanogaster*. Three- to six-day-old axenic *w*^*1118*^ *iso* males were inoculated for 6 hours, with different concentrations of *Acetobacter* OTU2753 (B), *A*. *cibinongensis* (C), *A*. *thailandicus* (D, E), *Leuconostoc pseudomesenteroides* (F), and *Lactobacillus brevis* (G). Bacterial levels were assessed 0 and 24 hours postinoculation. During this period, males were singly placed in cages, as shown in scheme (A). In (E), axenic chaser males were placed in cages together with males inoculated with *A*. *thailandicus*. At 24 hours, bacterial levels were assessed for both males. Bacterial levels between 0 and 24 hours decrease in flies inoculated with *Acetobacter* OTU2753 (lmm, *p* < 0.001), increase in flies inoculated with *A*. *cibinongensis*, *A*. *thailandicus*, and *L*. *brevis* (*p* = 0.024, *p* < 0.001, and *p* = 0.046, respectively), and do not significantly change in flies inoculated with *L*. *pseudomesenteroides* (*p* = 0.158). Supporting data can be found in S9 and S10 Data. (H) Proliferation of *A*. *thailandicus* in the gut of *D*. *melanogaster* and *D*. *simulans*. Three- to six-day-old *D*. *melanogaster* or *D*. *simulans* males were inoculated for 6 hours with *A*. *thailandicus* (10^4^ CFU/μL). Bacterial levels were assessed 0 and 24 hours postinoculation. During this period, males were singly placed in bottles. Three different genetic backgrounds for *D*. *melanogaster* (*w*^*1118*^ *iso*, *D*. *mel*. O13, and Canton-S) and for *D*. *simulans* (*D*. *sim*. J04, *D*. *sim*. O13, and *D*. *sim*. A07) were tested. Bacterial levels in the gut increase in *D*. *melanogaster* and decrease in *D*. *simulans* (lmm, *p* < 0.001). Supporting data can be found in S13 Data. Five individuals were analyzed for each condition, per replicate, and total number of CFUs per gut was determined by plating. Each dot represents one gut and the lines represent medians. Statistical analysis was performed together with replicate experiments shown in S10 Fig and S12C–S12E Fig. CFU, colony-forming unit; lmm, linear mixed model; *w*^*1118*^ *iso*, *w*^*1118*^ DrosDel isogenic strain.

We next asked to which extent these bacteria had the capacity to proliferate in the gut of *D*. *melanogaster*, because stability in the gut could be achieved through other mechanisms (e.g., bacteria could be simply attaching to the gut and avoiding elimination). Thus, we developed a protocol to analyze proliferation based on giving a small inoculum of bacteria and test if bacterial loads increase over 24 hours. We raised flies in axenic conditions and exposed 3–6-day-old males to different doses of bacteria. After 6 hours of feeding on the bacteria inoculum, flies were either collected to dissect and assess bacterial levels in the gut (0 hours) or placed singly in cages, as described above, and collected 24 hours later (Fig 5A). In this assay, *Acetobacter* OTU2753 did not colonize the gut of adult flies, and at the higher inoculum titers, the levels decreased between 0 and 24 hours (lmm, *p* < 0.001) (Fig 5B, S10A and S10E Fig), indicating that these bacteria cannot proliferate in the gut of *D*. *melanogaster*. On the other hand, the levels of *A*. *cibinongensis* and *A*. *thailandicus* increased in 24 hours (*p* = 0.024 and *p* < 0.001, respectively) (Fig 5C and 5D, S10B, S10C, S10F and S10G Fig), showing that these bacteria can proliferate in the gut of *D*. *melanogaster*. *A*. *thailandicus* proliferate more and reached higher levels than *A*. *cibinongensis* (*p* = 0.019). Interestingly, in flies exposed to *A*. *thailandicus* inocula superior to 10^2^ CFU/μL, these bacteria reach between 600 and 1,900 CFU per gut (Fig 4D, S10C and S10G Fig). These levels are similar to the stable compartment population size estimated above (1,300 CFU per gut), indicating that *A*. *thailandicus* can rapidly colonize a fly.

As all these *Acetobacter* species were able to grow on fly food (S11 Fig), it was still possible that the increase in the levels of *A*. *thailandicus* in the proliferation assay was due to a very fast growth on the fly food and re-acquirement by feeding. To test this possibility, we placed axenic (chaser) flies in cages simultaneously with the flies that had fed on *A*. *thailandicus* at time 0 hours of the experiment. At 24 hours, none of the axenic chaser flies had bacteria in their gut (Fig 5E and S10G Fig). This demonstrates that the levels measured in the inoculated fly were due to proliferation in the gut and not due to bacteria acquired from the food.

*L*. *pseudomesenteroides* levels did not significantly increase or decrease over 24 hours (lmm, *p* = 0.158) (Fig 4F and S10D Fig). At inocula superior to 10^2^ CFU/μL, *L*. *pseudomesenteroides* levels at 24 hours are between 150 and 550 CFU per gut. These results fail to show proliferation of *L*. *pseudomesenteroides* but indicate that this bacterium is not eliminated at the same rate as the unstable *Acetobacter* OTU2753.

Because *Lactobacillus* species are commonly found associated with *D*. *melanogaster* and shown to impact its physiology [25,26,28,37–39], we also tested isolates of *Lactobacillus paraplantarum* OTU1905 and *Lactobacillus brevis* OTU1870 in this assay (Fig 5G, S10H–S10J Fig). These *Lactobacillus* were isolated from the gut of wild flies at Day 0 of the stability assay (Fig 3G and 3H). *L*. *paraplantarum* levels do not change over 24 hours (lmm, *p* = 0.65) and can be sustained at 200–800 CFU per gut (similarly to *L*. *pseudomesenteroides*) (S10H and S10I Fig). On the other hand, the levels of *L*. *brevis* increase in 24 hours (*p* = 0.046), showing that this bacterium proliferates in the gut of *D*. *melanogaster* (Fig 5G and S10J Fig).

Overall, these assays show that *A*. *cibinongensis*, *A*. *thailandicus*, and *L*. *brevis* isolates proliferate in the gut of *D*. *melanogaster*. On the contrary, the transient *Acetobacter* OTU2753 cannot proliferate and is rapidly lost. *L*. *pseudomesenteroides* and *L*. *paraplantarum* have an intermediate phenotype in which proliferation is not shown, but the bacteria can sustain themselves in the gut over a period of 24 hours after oral inoculation.

### Gut proliferation of *A*. *thailandicus* is species specific

To test if proliferation of *A*. *thailandicus* in the gut is host specific, we compared its proliferation in *D*. *melanogaster* and *D*. *simulans*. These two species share the same habitat, feed on the same substrate, and are frequently captured together [40]. We used a proliferation protocol similar to the one described above (see figure legend, S12A and S12B Fig) to test three different genetic backgrounds of each host species. These included one isofemale line of each species that were collected simultaneously, from the same place as the initial collection of wild *D*. *melanogaster*. There is a significant difference in the colonization by *A*. *thailandicus* in these two host species (Fig 5H, S12C–S12E Fig, lmm, *p* < 0.001), with the levels increasing over 24 hours in *D*. *melanogaster* but decreasing in *D*. *simulans*. These results suggest that *D*. *melanogaster* and *A*. *thailandicus* interaction is host specific. Interestingly, although *A*. *thailandicus* colonizes all strains of *D*. *melanogaster* tested (Fig 5H, S12C–S12E Fig), there is variation in the growth at 24 hours, indicating modulation of this process by the host genotype (lmm, *p* = 0.002).

### *A*. *thailandicus* stable association with *D*. *melanogaster* is mutually beneficial

Symbiotic associations can range from pathogenic to mutualistic. Because *Acetobacter* species have been previously described as beneficial to *D*. *melanogaster* [16], we tested if the stable association between *D*. *melanogaster* with *A*. *thailandicus* could be advantageous for both. We started to test this hypothesis by comparing fitness parameters of flies monoassociated with *A*. *thailandicus*, *Acetobacter* OTU2753, and axenic flies, raised in standard fly lab food, by measuring time to pupariation and adulthood and total number of its progeny. Both *A*. *thailandicus* and *Acetobacter* OTU2753 monoassociated stocks had a much higher fertility than axenic flies and there was no significant difference between them (S13A and S13B Fig, lm, *p* < 0.001 for the comparisons of each *Acetobacter* monoassociation with axenic flies, in number of pupae or adults, *p* > 0.968 for the comparisons between *Acetobacter* monoassociated stocks). Flies monoassociated with either *Acetobacter* also developed until pupariation or adulthood approximately 3 days faster than axenic flies (S13C and S13D Fig, lm, *p* < 0.001 for each *Acetobacter* monoassociation comparison with axenic flies). Flies monoassociated with *Acetobacter* OTU2753 developed slightly faster to pupae (0.38 days) and adults (0.57 days) (*p* < 0.001 for each comparison). These results show that, in this setup, the association with either *Acetobacter* is clearly advantageous when comparing with axenic conditions and that the stable *A*. *thailandicus* does not provide a greater benefit than the lab isolate *Acetobacter* OTU2753.

**Fig 6:**
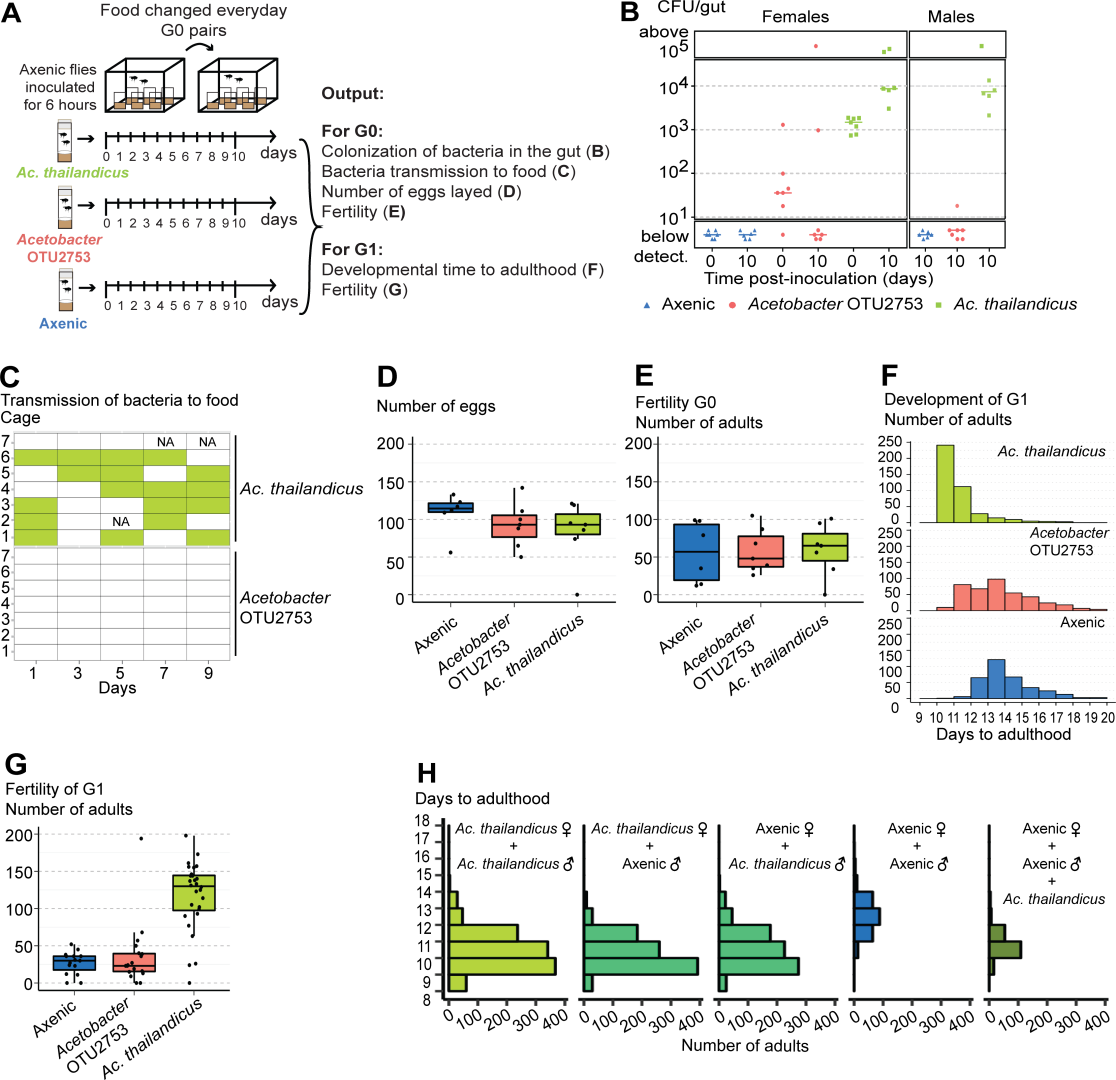
*Acetobacter thailandicus* stable association with *Drosophila melanogaster* is mutualistic. (A) Axenic 1–3-day-old *w*^*1118*^ *iso* males and females (G0) were in contact with an inoculum of 10^5^ CFU/μL of *Acetobacter* OTU2753, *A*. *thailandicus*, or sterile Mannitol (Axenic) for 6 hours. Two males and one female were placed per cage, with 6–7 cages for each condition, during 10 days, with food changed daily. This experimental setup corresponds to data shown in panels B–G. (B) Bacterial levels in single guts of females at time 0 (0 days) and 10 days postinoculation and in males 10 days postinoculation, analyzed by plating. Bacterial levels between the two time points increased in females inoculated with *A*. *thailandicus* and decreased in females inoculated with *Acetobacter* OTU2753 (Mann–Whitney test, *p* < 0.001 and *p* = 0.048, respectively). Supporting data can be found in S15 Data. (C) Presence of bacteria on the food collected from cages at days 1, 3, 5, 7, and 9 of the protocol, analyzed by plating. Filled rectangles represent presence of bacteria. NA stands for samples that were not analyzed. *A*. *thailandicus* is transmitted to the food with higher frequency than *Acetobacter* OTU2753 (glm-binomial, *p* < 0.001). Supporting data can be found in S16 Data. (D–G) Effect of bacterial association on the fitness of *D*. *melanogaster*. Total number of eggs laid by flies inoculated, or not, with different *Acetobacte*r (D) and total number of adults that emerged from these eggs (E). Total number of eggs or adults is not different between conditions (lmm, *p* > 0.484 for all comparisons). (F) Developmental time to adulthood of the progeny (G1) of flies inoculated, or not, with different *Acetobacter*. Developmental time to adulthood is faster in progeny from flies inoculated with *A*. *thailandicus* than in the other two conditions and in progeny from flies inoculated with *Acetobacter* OTU2753 compared to progeny from axenic flies (lmm, *p* < 0.001 for these comparisons). Supporting data can be found in S17 and S18 Data. (G) Fertility of G1 was assessed by placing two males and one female of G1 per vial, flipping them every other day for 10 days, and analyzing total number of emerged adults. Fifteen or more couples were made per condition. Fertility is higher in progeny from flies inoculated with *A*. *thailandicus* compared with the other two conditions (lmm, *p* < 0.001 for both comparisons) and not different in the comparison between the progeny of flies inoculated with *Acetobacter* OTU2753 or axenic (*p* = 0.592). Supporting data can be found in S19 Data. (H) One male and one female 1–2-day-old *w*^*1118*^ *iso*, either axenic or monoassociated with *A*. *thailandicus*, were placed in vials and flipped every other day for 10 days. To one set of vials with axenic parents, *A*. *thailandicus* was added on the eggs after passing the parents. Developmental time to adulthood of the progeny was assessed. Ten couples were made per condition. There are no differences on developmental time to adulthood if either or both parents are monoassociated with *A*. *thailandicus* (lmm, *p* > 0.412 for all these comparisons). Progeny from couples in which either or both parents are monoassociated and progeny from axenic flies in which *A*. *thailandicus* culture is added on the eggs develop faster than progeny from axenic flies (lmm, *p* < 0.001 for all these comparisons). Supporting data can be found in S20 Data. (B) Each dot represents one gut and lines represent medians. (D, E, and G) Each dot represents the total progeny of one female. All statistical analyses were done together with replicate experiments shown in S14 and S15 Figs. CFU, colony-forming unit; glm-binomial, generalized linear model with binomial distribution; lmm, linear mixed model fit; NA, not analyzed; *w*^*1118*^ *iso*, *w*^*1118*^ DrosDel isogenic strain.

However, the advantage of a stable association may not be revealed by directly studying monoassociated *D*. *melanogaster* stocks. In these conditions the bacteria are continuously associated with *D*. *melanogaster*, even if they are only present in the food or transiting through the gut. But in the wild, *D*. *melanogaster* adults freely move in space and can explore a continuously changing environment, a situation in which a stable association could be important. Therefore, we established a protocol to test the fitness benefits of the stable interaction in a scenario that simulates this changing environment. After 6 hours of feeding on an inoculum of bacteria, one female and two males were placed per cage and maintained there for 10 days, with food being changed daily (Fig 6A). After 10 days of this protocol, males exposed to *A*. *thailandicus* have a median of 6,800 CFU per gut (Fig 6B and S14A Fig), showing that colonization can be sustained for a long time. In females, *A*. *thailandicus* grows in the gut between the beginning of the experiment and 10 days in the cage (Wilcoxon rank sum test, *p* < 0.001) and reaches a median of 17,500 CFU per gut. These results show that *A*. *thailandicus* also colonizes and proliferates in female *D*. *melanogaster*. On the other hand, *Acetobacter* OTU2753 levels decrease between Day 0 and Day 10 in females (*p* = 0.048) and both sexes have a median of 0 CFU per gut at Day 10, confirming that flies are not colonized by these bacteria (Fig 6B and S14A Fig).

As a measure of the fitness benefit for the bacteria, we tested if they could be transmitted to the food. We analyzed bacterial transmission by flies during the experiment at days 1, 3, 5, 7, and 9. Flies associated with *A*. *thailandicus* transmitted bacteria to the food with a much higher frequency than flies associated with *Acetobacter* OTU2753, in which transmission occurred only once (Fig 6C, S14B Fig, generalized linear model with binomial distribution [glm-binomial], *p* < 0.001). Moreover, the probability of transmission of *A*. *thailandicus* to the food was independent of the day of the experiment (ANOVA on glm-binomial models, *p* = 0.811). These results show that, upon gut colonization, *A*. *thailandicus* can be continuously transmitted by *D*. *melanogaster*. The stable association may be advantageous to the bacteria and mediate their dispersal in the environment.

To compare the effect of this association on host fitness, we started by analyzing the fertility of the flies in terms of number of eggs laid and adult progeny, during the experiment. The number of eggs or adult progeny were not significantly different between axenic flies and flies exposed to either bacteria (Fig 6D and 6E, S14C and S14D Fig, lmm, *p* > 0.484 for all comparisons). However, the time that these embryos took to reach adulthood was different. Progeny from flies colonized by *A*. *thailandicus* developed 2 or 3 days faster than progeny from flies previously exposed to *Acetobacter* OTU2753 or axenic flies, respectively (Fig 6F, S14E Fig, lmm, *p* < 0.001 for both comparisons). However, the progeny of flies exposed to *Acetobacter* OTU2753 developed only 0.6 days faster than axenic flies (*p* < 0.001). Moreover, the fertility of this progeny was strongly influenced by the interaction of their parents with bacteria. The progeny from flies previously colonized by *A*. *thailandicus* had a much higher fertility than the progeny from flies previously exposed to *Acetobacter* OTU2753 or axenic flies (Fig 6G, S14F Fig, lmm, *p* < 0.001 for both comparisons), while there was no difference between the progeny of flies exposed to *Acetobacter* OTU2753 or axenic flies (*p* = 0.592). These data show that the interaction of adult flies with stable bacteria does not affect their fertility but has a strong influence on the development and fertility of its progeny.

This transgenerational effect could be due to an effect of the stable *A*. *thailandicus* gut population on the parents and a subsequent indirect effect on the progeny, or through the transmission of the bacteria to the next generation and its effect during larval development. We tested if the developmental time of the progeny was dependent on the bacterial association with either parent by analyzing the four possible couple combinations of flies raised axenically or monoassociated with *A*. *thailandicus* (Fig 6H, S15 Fig). There is no difference in developmental time to pupariation or adulthood if either or both parents are from the monoassociated stock (lmm, *p* > 0.412 for all these comparisons). The progeny of these three crosses develop, on average, 2.7–2.8 days faster than the progeny of crosses with both parents axenic (*p* < 0.001 for all comparison). These results show that the transgenerational effect on developmental time is not specifically associated with the mother or the father. Also, adding *A*. *thailandicus* to the progeny of axenic flies rescues the developmental delay. When bacteria are added, these flies develop approximately 2 days faster (*p* < 0.001). This is not a full rescue because axenic eggs plus *A*. *thailandicus* still develop, on average, 0.5–0.8 days slower than flies with either or both parents from monoassociated stocks (*p* < 0.001 for all comparisons). This may be explained by the fact that the bacteria are only added when the parents are removed from the vial, after 2 days of egg laying. These data are compatible with a scenario in which flies associated with *A*. *thailandicus*, either male or female, can transmit the bacteria to the next generation, which then plays an important role in its development. In agreement with this hypothesis, we have shown above that *A*. *thailandicus* can be continuously transmitted to the environment (Fig 6C, S14B Fig). Moreover, we detected bacteria in the surface of 20 out of 20 eggs laid by flies monoassociated with *A*. *thailandicus*, by testing bacterial growth in medium. This demonstrates that *A*. *thailandicus* is efficiently transmitted from mothers to their progeny.

We also observed that *A*. *thailandicus* affected the fertility of *D*. *melanogaster* in this assay. Similarly to the results above, there is no difference in total number of progeny if either or both parents are from the monoassociated stock (S15B, S15D, S15F and S15G Fig, pupae or adult number, lm, *p* > 0.180 for all these comparisons). However, if both parents are axenic, the number of pupae or adults is lower (*p* < 0.001 for all comparisons). This lower number of pupae or adults is not rescued by adding *A*. *thailandicus* to the axenic eggs (*p* = 0.998), indicating that these bacteria are not affecting egg to pupae or adult survival. Because exposing axenic adults to *A*. *thailandicus* does not alter their fertility (Fig 6D and 6E, S14C and S14D Fig), this fertility effect may be dependent on either parent development in the presence of *A*. *thailandicus* or in the presence of *A*. *thailandicus* in the fly food for the 2 days of the egg laying.

**Fig 7:**
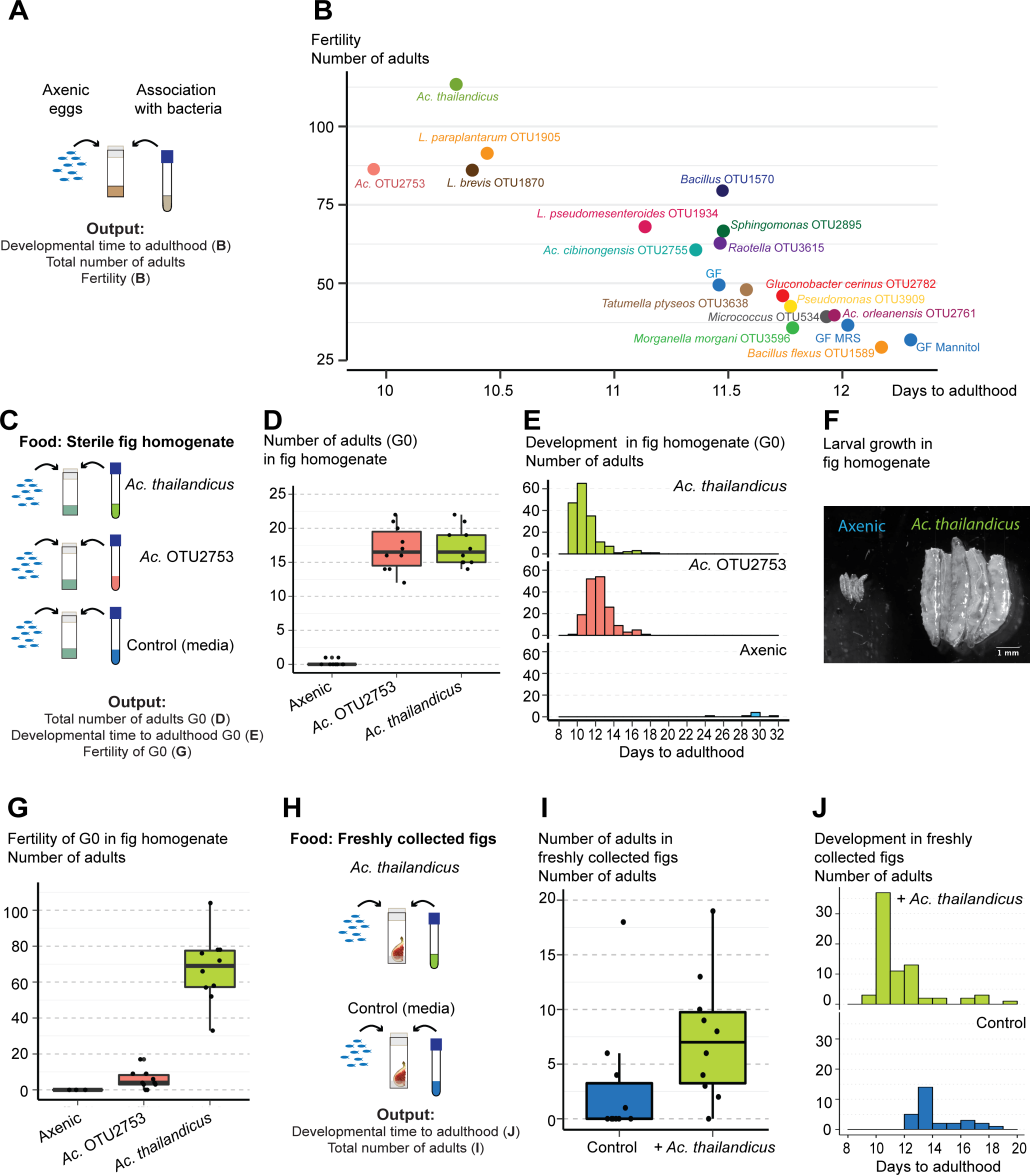
*Acetobacter thailandicus* is beneficial in the context of other wild bacteria and natural food substrates. (A) *w*^*1118*^ *iso* eggs were associated with different bacteria isolated from the gut of wild-caught *Drosophila melanogaster*. As controls, axenic eggs that had no treatment (GF) or in which sterile media were added (GF MRS and GF Mannitol) were used. (B) For each bacterium, estimates of developmental time to adulthood of these eggs are plotted against estimates of their fertility. These estimates derive from the statistical analysis of data presented in S16C–S16F Fig. There is a negative correlation between developmental time and fertility (Pearson correlation −0.91, *p* < 0.001). Supporting data can be found in S21 and S22 Data. (C) Thirty axenic *w*^*1118*^ *iso* eggs were placed in vials containing sterilized fig homogenate. *A*. *thailandicus*, *Acetobacter* OTU2753, or sterile culture media were added on the top of the eggs. Ten vials were used per condition. Total number of adults that emerged (D) and developmental time to adulthood (E) was determined. More eggs inoculated with *A*. *thailandicus* and *Acetobacter* OTU2753 developed to adulthood and faster than axenic eggs (lmm, *p* < 0.001 for both comparisons). Supporting data can be found in S23 Data. (F) Larvae 5 days postinoculation with *A*. *thailandicus* or sterile media in fig homogenate. (G) Progeny of flies developed in fig homogenate, with and without the addition of *Acetobacter* species. One male and one female were collected from G0 of each condition and placed per vial containing fig homogenate for 10 days, with vials flipped every other day. *A*. *thailandicus* and *Acetobacter* OTU2753 conditions have 10 replicates, but only 3 from axenic eggs were possible to perform. Flies that developed with *A*. *thailandicus* had more progeny than flies that developed with *Acetobacter* OTU2753 or sterile media (lmm, *p* < 0.001). Supporting data can be found in S24 Data. (H) Fifty axenic *w*^*1118*^ *iso* eggs were placed in vials containing freshly collected nonsterile figs. *A*. *thailandicus* culture or sterile media (Control) was added on the top of the eggs. The total number of adults that emerged (I) and their developmental time to adulthood (J) was analyzed. Ten vials were analyzed per condition. There were more adults emerging from vials inoculated with *A*. *thailandicus* (lmm, *p* = 0.010). Developmental time to adulthood was faster in eggs inoculated with *A*. *thailandicus* in this experimental replicate but was not significantly different in the other replicate represented on S18K Fig (lmm, *p* < 0.001 and *p* = 0.557, respectively). Supporting data can be found in S25 Data. Statistical analyses from (D–J) were done together with replicate experiments shown in S18 Fig. GF, germ free (axenic); lmm, linear mixed model fit; MRS, de Man, Rogosa and Sharpe broth; *w*^*1118*^ *iso*, *w*^*1118*^ DrosDel isogenic strain.

The results above suggest that a stable association with gut bacteria is beneficial to adult *D*. *melanogaster*, because it allows continuous transmission to the next generation, promoting its faster development and higher fertility. However, these experiments were performed by providing axenic food to flies, and in a natural scenario, flies are bound to encounter many other bacteria present in the food substrates. If all bacteria were equally beneficial for fly development, this stable association could be irrelevant. Therefore, we tested if different bacteria naturally encountered by *D*. *melanogaster* confer different fitness benefits to the flies. We sterilized eggs of *w*^*1118*^ *iso* and associated them with different bacteria found in the gut of flies from a natural population (sampled from the isolates of Fig 2, Fig 7A). We determined total number of adults that developed from these eggs, their developmental time, and their fertility. The number of adults that emerged (G0) was not different between associations with different bacteria or in germ-free conditions (S16A and S16B Fig, lmm, *p* > 0.282 for all pairwise comparisons). However, we did observe differences in the developmental time and fertility of these adults associated with different bacterial isolates, and found a negative correlation between these parameters (Pearson correlation −0.91, *p* < 0.001) (Fig 7B, S16C–S16F Fig, S17 Fig). Flies associated with *A*. *thailandicus* developed faster and are more fertile than axenic flies and flies associated with most of the other tested bacteria. Flies associated with *Acetobacter* OTU2753, *L*. *brevis*, and *L*. *paraplantarum* developed as fast and are as fertile as *A*. *thailandicus* (*p* > 0.200 for these pairwise comparisons). While flies associated with *A*. *cibinongensis* developed slower than with *A*. *thailandicus* (*p* = 0.023), the developmental time of flies with *L*. *pseudomesenteroides* is not significantly different (*p* = 0.224). However, both have lower fertility than flies with *A*. *thailandicus* (*p* < 0.001). Flies associated with *Bacillus flexus* OTU1589 were not different from axenic flies in terms of developmental time or fertility (*p* = 0.878). Overall, these data demonstrate that different bacteria have a variable effect on the development and fertility of *D*. *melanogaster*, with some not conferring any advantage to the flies’ development or fertility. *A*. *thailandicus* seems particularly beneficial to *D*. *melanogaster* and, therefore, the stable association may be advantageous to the host.

We also analyzed the impact of *A*. *thailandicus* on *D*. *melanogaster* fitness when they develop in fruit, a more natural food substrate, instead of standard fly food. We compared development from eggs to adults on a sterile fig homogenate, with the addition of *A*. *thailandicus*, *Acetobacter* OTU2753, or sterile medium (Fig 7C). The association with both *Acetobacter* strongly influenced the number of emerging adults, with very few flies reaching adulthood in axenic conditions (Fig 7D, S18A–S18C Fig, lmm, *p* < 0.001). Moreover, flies associated with *A*. *thailandicus* develop 1.5 days faster than flies associated with *Acetobacter* OTU2753 (Fig 7E, S18F Fig, lmm, *p* < 0.001). The few axenic flies that reach adulthood are slower and take on average 27 days (Fig 7E, S18D–S18F Fig, lmm, *p* < 0.001). This reflects a delay in growth because 5-day-old larvae in axenic conditions were much smaller than larvae with *A*. *thailandicus* (Fig 7F). We subsequently tested the number of progeny of flies that developed in these three conditions. Interestingly, the number of progeny was much higher in adults that developed on figs in the presence of *A*. *thailandicus* than in the presence of *Acetobacter* OTU2753 or axenic adults (Fig 7G, S18I Fig, lmm, *p* < 0.001). These results show that the benefit of *A*. *thailandicus* for the development and fertility of flies is even more pronounced in a natural food substrate.

Because, in nature, fruits are not sterile and flies develop in the presence of different microbial communities, we decided to test the potential benefit of *A*. *thailandicus* in freshly collected figs. We compared the development of sterilized eggs in natural collected figs in the presence or absence of *A*. *thailandicus*. Flies grown in the presence of *A*. *thailandicus* had approximately double the survival rate to adulthood of control flies with no bacteria added (Fig 7I, S18J Fig, lmm, *p* = 0.010). This is similar to the effect seen in sterile figs. The effect of *A*. *thailandicus* on the time to reach adulthood varies with replicate (Fig 7J, S18K Fig, lmm, *p* < 0.001). In one replicate, the presence of bacteria does not affect time of development (S18K Fig, *p* = 0.557), while in the other replicate, *A*. *thailandicus* decreases time of development by 3.5 days (Fig 7J, *p* < 0.001). This difference between replicates may reflect the variable bacteria consortiums in the figs collected from the tree at different times. These results support that the stable association between *D*. *melanogaster* and *A*. *thailandicus* is beneficial for the flies in their natural environment.

## Discussion

Here, we identify bacterial isolates from a natural population of *D*. *melanogaster* that can proliferate and stably colonize the gut of their host. These results demonstrate that *D*. *melanogaster* has bona fide gut bacterial symbionts in the wild. We further show that the association with one of these gut bacterial symbionts, *A*. *thailandicus*, can be mutually beneficial. On one hand, stable colonization of *D*. *melanogaster* gut permits continuous bacterial shedding to the environment, therefore potentially increasing bacterial dispersion in the wild. On the other hand, transmission of *A*. *thailandicus* to the food substrate, concomitant with egg laying, benefits *D*. *melanogaster* larval development. These bacteria shorten developmental time and increase fertility of *D*. *melanogaster*. This stable interaction may be particularly important for *D*. *melanogaster* because different bacteria differentially affect its development, and *A*. *thailandicus* is more beneficial than most bacteria sampled from the gut of wild flies. Moreover, *A*. *thailandicus* is still beneficial when larvae develop in nonsterile fruit collected from nature.

### Diversity and stability of gut bacteria in wild and laboratory *D*. *melanogaster*

The several protocols we developed were mainly based in culture-dependent techniques, in order to quantify absolute levels of live bacteria in the gut. Gut bacteria of *D*. *melanogaster* previously identified through 16S rRNA gene sequencing [20,26,28,41–46] belong to genera that can also be identified by culture-dependent techniques; however, it is possible that our approach missed gut bacteria that do not grow in the media or conditions that we used. Additionally, our approach mainly identifies the bacterial strains that are more abundant in the gut, as there is a limited number of colonies in the plates analyzed. Because of these limitations, our analysis may be incomplete. Nonetheless, our approach managed to quantify overall gut bacterial numbers in different husbandry conditions, and, when tested, the results were confirmed by qPCR. Moreover, we were able to identify, isolate, and analyze bacteria that can stably associate with *D*. *melanogaster* gut.

Our results show a striking difference in gut bacterial diversity between lab and wild-caught flies. Lab flies carry mainly two bacterial species corresponding to *Acetobacter* OTU2753 and *Lactobacillus* OTU1865. This low diversity and dominance of *Acetobacter* and *Lactobacillus* species is in agreement with several previous studies on the gut-associated bacteria in lab flies [20,22,24–27]. On the other hand, we were able to identify 35 different OTUs in the 10 individual flies freshly collected from the wild, and the sampling did not seem close to saturation. This higher diversity is also in agreement with previous reports [25,28]. The characterization of individual flies allowed us to identify Enterobacteriaceae, Acetobacteriaceae (mainly *Acetobacter* and *Gluconobacter* species), Leuconostocaceae, and Bacillaceae as the most prevalent families, present in over 50% of the flies. These families of bacteria have been identified before in wild-caught *D*. *melanogaster*, although Bacillaceae are found less frequently [25,28,41–43,46]. *Lactobacillus* was only found in 5 out of 20 wild-caught flies. Although the low prevalence of *Lactobacillus* could be a characteristic of this specific population, it is a general trend observed in other published surveys [25,28,41–43,46].

The *Acetobacter* and *Lactobacillus* species associated with our laboratory stock cannot stably persist in the gut in the absence of reinfection, and they grow on the fly food, similar to what was reported before [20]. Thus, these bacteria are only transiently passing through the gut. This result highlights how husbandry conditions can affect *D*. *melanogaster* gut bacterial levels and that these measured levels can be unrelated with gut colonization (also shown in [20,27]).

In contrast to lab flies, wild-caught flies carry bacteria that can persist in the gut of *D*. *melanogaster*. This shows that in its natural state *D*. *melanogaster* lives with gut-colonizing bacteria. *L*. *pseudomesenteroides*, *A*. *cibinongensis*, and *A*. *thailandicus* were each present in more than 50% of wild flies at the end of the stability protocol. They are, therefore, interesting bacteria to further characterize in their interaction with *D*. *melanogaster*.

*A*. *cibinongensis* and *L*. *pseudomesenteroides* have been previously studied in wild and lab *Drosophila* species by culture-dependent and -independent techniques [31,47–55]. *A*. *thailandicus* was recently isolated from the gut of lab *D*. *melanogaster* by culture methods [56]. Association of this species with *D*. *melanogaster* may have been missed in previous studies because the *A*. *thailandicus* 16S rRNA gene sequence was only recently available [57] and is very similar to this gene in other *Acetobacter* species.

Several bacteria were present in 50% or more of the flies when they were caught, but were severely reduced in frequency after the stability protocol. These species may be transient gut bacteria that were acquired from the environment. However, it is also possible that they are stable gut bacteria that cannot be sustained in the particular lab environment we used. For instance, in the fly food we used, there may be nutritional requirements missing for their maintenance or there could be compounds toxic to them (e.g., methylparaben). In the future, this protocol could be repeated using another food source, for example, the fruit matching the source of capture. However, the natural environment of *D*. *melanogaster* is very complex and includes decomposing and fermenting fruits replete with different microorganisms. Therefore, it will be difficult to study bacterial stability under conditions that completely match the ones found in nature.

At the end of the stability protocol, there was still a high diversity of bacteria in the gut of *D*. *melanogaster*, even if most were present in less than 50% of the flies. These may represent rare but stable gut bacteria of *D*. *melanogaster*, as in the case of *Lactobacillus* species. A particular fly (fly 39 in Fig 2) has an interesting pattern of microbiota composition, with six rare OTUs persisting at relatively high levels and without Lactobacillales or Acetobacteriaceae. This gut microbiota composition may represent a disease-related dysbiosis, and some of these bacteria could be pathogenic.

### Gut colonization by *A*. *thailandicus* and *A*. *cibinongensis*

In contrast to the lab isolate of *Acetobacter* OTU2753, both wild isolates of *A*. *cibinongensis* and *A*. *thailandicus* persist in the gut of lab flies when monoassociated, until the end of the stability protocol. However, the levels of both bacteria decreased significantly in the first 2 days of this assay, indicating that the majority of the bacteria found in the gut of these flies at the beginning of the experiment were transient. Nevertheless, both bacteria have the autonomous property to persist in the host, independently of other microbiota members. Moreover, this property seems largely independent of host background, because it is observed in the *w*^*1118*^ *iso* lab flies and in several individuals of the natural outbred population. In addition to being able to stably colonize, both *A*. *thailandicus* and *A*. *cibinongensis* proliferate in the gut of *D*. *melanogaster*, showing that these bacteria are bona fide *D*. *melanogaster* gut colonizers.

The niche of the stable population of *A*. *thailandicus* is the foregut of *D*. *melanogaster*. Live bacteria were consistently observed in the anterior part of the crop, crop duct, and proventriculus and absent in the midgut and hindgut. Light and electron microscopy show that these bacteria are present in clusters. The bacteria seem to be attached to each other by fimbriae, although the nature of these extracellular appendages needs future confirmation. This organization in clusters may contribute to the stability of the bacteria in the folds of the crop or in the proventriculus by physically decreasing their changes of being dragged through the gut. Additionally, the crop is a diverticulum of the esophagus that can store liquid food, and its lumen is not subject to the same linear flux as the rest of the gut [58,59], which might facilitate bacterial persistence. A similar argument is made for the appendix and cecum in humans and other mammals, as a reservoir of microbiota [60,61]. Another possibility is that *A*. *thailandicus* attaches to the cuticle lining of the foregut. Fimbriae, or other appendages, can also be involved in adherence to the host [62]. However, we did not observe direct adherence to the host by electron microscopy. In some instances, we saw close proximity between bacterial clusters and chitin folds of the crop, but a more thorough analysis will be required to assess this.

We observed a clear border in *A*. *thailandicus* colonization between the proventriculus and the midgut. This border may work as a physical or immunological barrier for microorganisms in insects [27,63–65], and the acidic region in the anterior midgut may also contribute to bacteria killing [38,66]. Also, the continuous secretion and movement of the peritrophic matrix, which separates the gut lumen from epithelial cells, may hamper stable bacterial colonization [67,68].

The crop was identified 130 ago as the region where yeasts proliferate in flies [69]. Several bacteria have also been shown to colonize the foregut in *D*. *melanogaster* and other insects, including the human pathogen *Yersinia pestis* and the plant pathogen *Xylella fastidiosa* [70–74]. Regurgitation of the foregut content has been implicated in transmission of *Y*. *pestis* to humans and transmission of bacteria to plant surfaces by *Bactrocera* and *Ceratitis* fruit flies [59,72,73,75]. This process may also be involved in transmission of *A*. *thailandicus* by *D*. *melanogaster*.

### *D*. *melanogaster* and *A*. *thailandicus* mutualism

Given the stable association between *D*. *melanogaster* and *A*. *thailandicus*, we asked if there was any advantage for either partner in this interaction. Symbiosis between a host and a microbe does not necessarily signify mutualism, and the effect of host association on the microbial partner has been less frequently studied [76,77]. Our results indicate that the stable association of *A*. *thailandicus* to the gut of the adult fly is advantageous to this bacterium because it can promote its dispersal.

The interaction with *A*. *thailandicus* is also advantageous to *D*. *melanogaster* in several scenarios. *A*. *thailandicus* shortens larvae developmental time, and this can contribute to an increased host fitness if there are no associated trade-offs [37,78]. Interestingly, adult flies that developed in the presence of *A*. *thailandicus* are also more fertile, a clear measure of fitness, when compared with flies that developed axenically. Other bacteria have been shown before to shorten the developmental time of *D*. *melanogaster* [15,16,79–82] and increase adult fertility when associated in larval stages [83]. However, out of the 15 bacteria isolated from wild flies, *A*. *thailandicus* induced the shorter development time and higher fertility. Therefore, out of the set of bacteria interacting with *D*. *melanogaster* in the wild, this stable gut symbiont is particularly beneficial.

We do not know the mechanism through which *A*. *thailandicus*, or the other bacteria we tested, benefit *D*. *melanogaster*. The negative correlation that we observed between developmental time and fertility suggests a similarity in the mechanisms behind these phenotypes. Microorganisms have long been recognized as important for *Drosophila* development and as a source of food [14,84]. In fact, the standard *Drosophila* food used in the lab is partly composed of dead *Saccharomyces cerevisiae* [85], which, in this diet, is required and sufficient for *Drosophila* development. Moreover, in lab diets the bacterial influence on host development is generally stronger the less yeast extract the food contains [15,16]. In *D*. *suzukii*, high doses of heat-killed bacteria and yeast can decrease the developmental time to the same extent as the same strains alive [86]. Also, in *D*. *melanogaster* adults, a constant supply of heat-killed yeast *Issatchenkia orientalis* can extend the life span of flies to the same extent as live yeast [19]. The nutritional value of these microorganisms may be based on supplying amino acids or vitamins to the host [14,19,49,84,87]. Other evidence indicates that the effect of microorganisms on development of *D*. *melanogaster* has a component independent of its nutritional value, and heat-killed bacteria are not sufficient to fully rescue the phenotype conferred by live bacteria [38]. Bacteria can directly impact host physiology by activating the insulin pathway via acetic acid production in the case of an *A*. *pomorum*, or gut proteases in the case of *L*. *plantarum* [16,39,88].

The benefit of *A*. *thailandicus* for *D*. *melanogaster* becomes even more evident when larvae develop in figs, a natural food substrate. On sterile figs homogenates, very few larvae reach adulthood in axenic conditions, and those that do are severely delayed in growth and are infertile as adults. These results show the insufficiency of fruit, or figs in this particular case, to support normal *D*. *melanogaster* development. *A*. *thailandicus* rescues these phenotypes and is, therefore, sufficient for *D*. *melanogaster* development on fruit, indicating a nutritional basis for the interaction. Interestingly, developmental time of flies is shorter and fertility is higher with the addition of *A*. *thailandicus* to figs than with the addition of *Acetobacter* OTU2753, contrary to what happens in the laboratory food. This may indicate adaptation of these bacteria to their food source and consequent impact on the host [89].

An alternative hypothesis is that bacteria are detoxifying some toxic components present on the food. Detoxifying symbiosis is known to occur in many insects [90]. However, the fact that *A*. *thailandicus* is beneficial both in lab food and figs indicates that to a large extent its benefit is independent of food toxins.

We did not see a direct effect of the stable *A*. *thailandicus* population on adults’ fertility. However, direct effects of bacteria on adults have been previously reported on oocyte development or fertility [83,91]. Many factors may explain the different results, including the identity of the bacteria tested and the relatively small bacterial stable population in the gut. Nonetheless, it will be interesting in the future to determine if the stable *A*. *thailandicus* population has any other effect on the adult physiology.

### Gut colonization by *Leuconostoc* and *Lactobacillus*

Analysis of *L*. *pseudomesenteroides* stability and proliferation in *D*. *melanogaster* gut produced ambiguous results. This bacterium seemed very stably associated with the gut of wild and monoassociated lab flies when the stability protocol was performed in vials. When we implemented the protocol using cages, however, it disappeared from 50% of the flies. These results illustrate how sensitive to experimental conditions this assay is, and that stringency is crucial. The proliferation assay did not clearly show an increase or decrease in *L*. *pseudomesenteroides* at 24 hours, when compared to the beginning of experiment. These results could be the consequence of this bacterium being able to very rapidly proliferate in the gut of the fly but unable to attach to the host and, therefore, requiring a constant cycle of reinoculation. Maybe this cycle could be kept in vials but broken down in cages. Further experiments will be required to test this hypothesis and elucidate the interaction of *L*. *pseudomesenteroides* with *D*. *melanogaster*.

*Lactobacillus* species were still present in wild flies at the end of stability protocol, although less frequently than *A*. *thailandicus*, *A*. *cibinongensis*, and *L*. *pseudomesenteroides*. Interestingly, the data indicate a negative interaction between *Lactobacillus* and *Leuconostoc* presence. Both are lactic acid bacteria (order Lactobacilalles), and they may occupy the same niche and compete for resources. Of the many bacterial isolates from the gut of wild flies, *L*. *brevis*, and *L*. *paraplantarum* are the most beneficial in terms of development time and fertility of *D*. *melanogaster*, together with *A*. *thailandicus*. This contrasts with previous reports indicating a small or null effect of lab *Lactobacillus* isolates on fecundity [37,83]. *L*. *brevis* is present in 4 out of 10 wild flies after the stability protocol and proliferates in the gut of *D*. *melanogaster*. So, *L*. *brevis* may also be a beneficial bona fide gut symbiont of *D*. *melanogaster*, although not as frequent as *A*. *thailandicus* in this population.

### Ecological advantage of a stable gut association with beneficial bacteria

**Fig 8:**
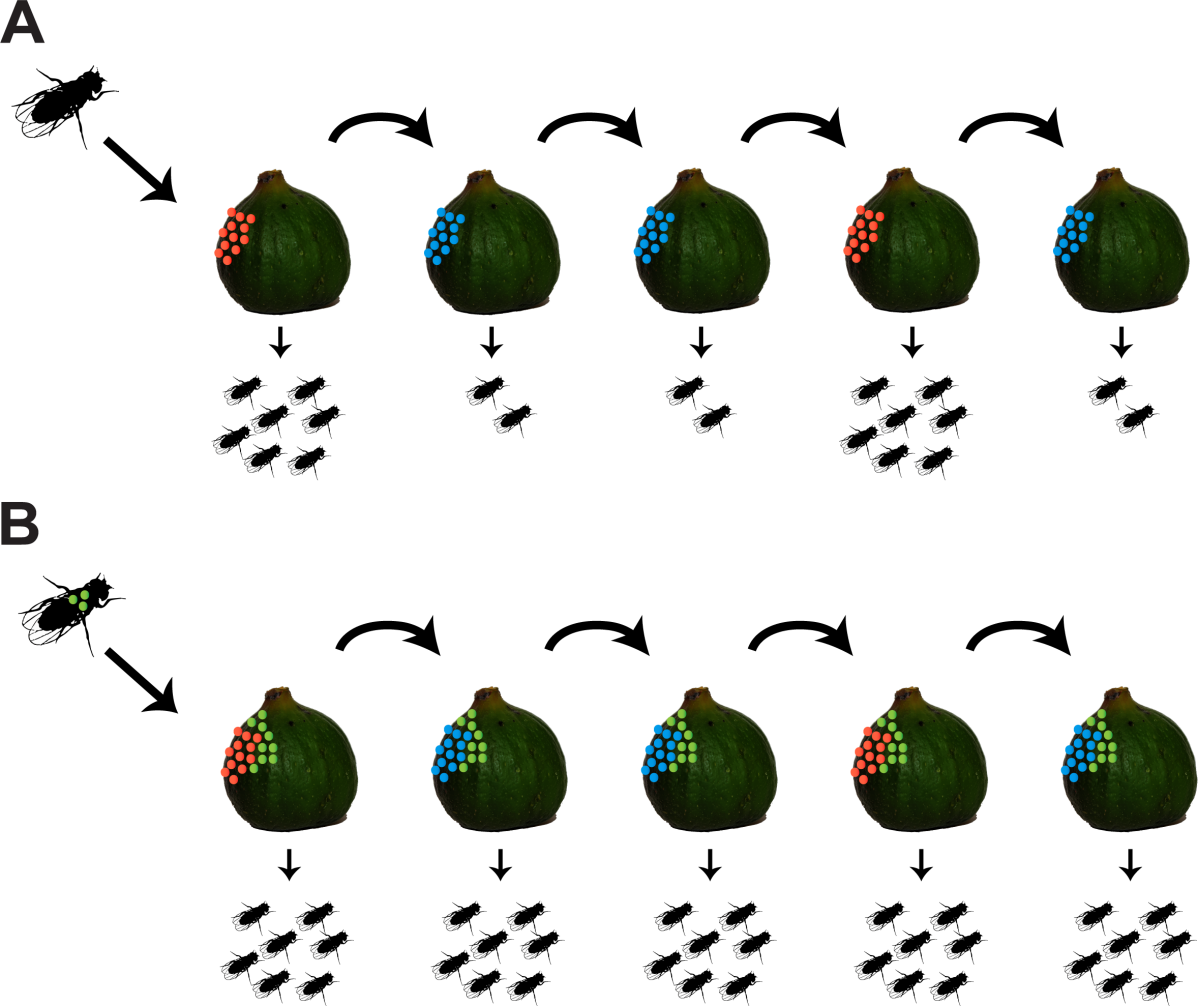
Model for an ecological advantage of a stable association between *Drosophila melanogaster* and beneficial gut bacteria. (A) In the absence of stable gut bacteria, the fitness of *D*. *melanogaster* is dependent on the presence of more (red) or less (blue) beneficial bacteria in the food substrate. (B) Carrying a stable population of beneficial bacteria (green) in the gut allows constant bacterial inoculation of food substrate and consequent association with the next host generation. This leads to a higher fitness of this next generation.

Our results indicate that the interaction between *D*. *melanogaster* and the gut symbiont *A*. *thailandicus* is especially beneficial for both partners in the wild (Fig 8). The small stable bacterial population in the gut serves as a reservoir for the inoculation of the environment that the adult fly explores and exploits. This is beneficial to the bacteria because it leads to their continuous dissemination. On the other hand, transmission of *A*. *thailandicus* to the food substrate of the next generation, concomitant with egg laying, benefits *D*. *melanogaster* development. This association is therefore a form of farming, a strategy adopted by several insects, including ants, termites, and ambrosia beetles with fungi [92]. The stability of the *D*. *melanogaster–A*. *thailandicus* interaction provides the host some independence from the local bacterial populations and enables it to explore and modulate bacterial populations in new locations.

Besides the interaction with these stable bacteria in the wild, *D*. *melanogaster* also interacts with a plethora of environmental bacteria and yeasts that may be transiently associated with the gut. Many of these non-colonizing microorganisms probably positively impact *D*. *melanogaster* biology, and vice versa. *D*. *melanogaster* are attracted to feed on, or oviposit in, substrates with specific potential benefiting bacteria and yeasts [14,91,93–97]. Attraction to fermenting fruits enriched with beneficial microbes may be a strategy adopted by *D*. *melanogaster* to increase interactions with these bacteria. Furthermore, *D*. *melanogaster* most likely disperses them as they transit through its gut. However, if these bacteria or yeasts are not stably associated with the flies, this would be a transient phenomenon. *D*. *melanogaster* may also benefit bacteria by promoting their growth in the food substrate [38], which could be advantageous for the host if biased towards beneficial bacteria. Despite all these potential mechanisms promoting beneficial interactions, relying on the immediate environmental and local microbial community may be suboptimal for *D*. *melanogaster* (Fig 8).

In the future it will be interesting to address some questions relevant for this model. For instance, we do not know how stable *A*. *thailandicus* is in the gut of larvae or if this stability is important. It may be sufficient for the bacteria to grow on the food substrate, because larvae are less mobile and they will be in constant contact with the local external population of bacteria. Another important aspect is to understand how adult flies acquire *A*. *thailandicus*. This could be through constant association throughout the developmental stages, including from larvae to pupae to adult, or de novo acquisition after adult eclosion [79].

This farming interaction model may extend to other bacteria, including *L*. *brevis*. Moreover, our study focused on the gut-colonizing bacterial species in one *D*. *melanogaster* population. It will be important to analyze other natural populations, from other diets and geographical regions, and determine to what extent there is conservation of stably colonizing species. This analysis could elucidate if there is a core gut microbiota of *D*. *melanogaster* based on stable symbionts. It will also be important to extend this analysis to other microbes, such as yeasts, given that flies are constantly exposed to them in the natural habitat.

Interactions between microbes may affect their colonization and their influence on host phenotypes. These may happen with other colonizing bacteria, with environmental bacteria on the food substrate, or while in transit through the gut. Our analysis of wild-caught flies incorporates, to a certain degree, this complexity. For instance, *A*. *thailandicus* that stably colonizes in monoassociation is also present in the gut of the majority of wild flies of the population we analyzed, showing that its association is robust in the face of rich bacterial communities. Moreover, the beneficial effect of this bacterium observed in monoassociation is also present in the context of complex and natural microbial communities of figs. On the other hand, the analysis of wild-caught flies also indicates a negative interaction between *Lactobacillus* and *Leuconostoc* species.

### Specificity of gut symbionts

We show that stable interactions are specific from both host and the bacterial perspectives. Subtle differences in the bacteria associated with *D*. *melanogaster* and *D*. *simulans* in the wild have been found before [28], but differences may be clearer when looking into the stable gut symbionts of different *Drosophila* species.

The presence of these species-specific mutualistic interactions of gut bacteria with *D*. *melanogaster* raises the possibility that these are long-term interactions and the result of adaptation. Therefore, they may be a good system to study host-symbiont evolution and even address questions of coevolution and cospeciation [30,98–100].

We still do not know the cause of the specificity of these colonizations. The interaction between the host immune system and different bacteria could be one of the mechanisms involved in this selection. Bacteria can down-regulate or escape from the host immune system to establish infection [101], and in *D*. *melanogaster*, alterations in immunity have an impact on gut bacterial compositions or load [22,27,102]. Many innate immune genes in *Drosophila* species are under fast positive selection [103–105], and differences in these genes could mediate association of different *Drosophila* species with different stable gut bacteria.

### Stable gut bacteria in *D*. *melanogaster* as an experimental system

Although the perspective of a transient microbiota has been dominant in most analyses of gut bacteria in *Drosophila* [20,27,38,43,106], there is some evidence of stable gut bacteria in these flies. Recently it was shown that a wild isolate of *L*. *plantarum* has a higher frequency of gut colonization than a lab isolate [31]. These results are in agreement with a tendency for wild isolates of bacteria being better at colonizing *D*. *melanogaster*. However, in this study, once bacterial colonization was established, titers were constant over time in wild and lab isolates [31]. It will be interesting to also test these isolates with the proliferation and stability protocols that we describe here. On a different approach, analysis of wild-caught individuals from mushroom, and cactus-feeding *Drosophila* species have identified bacterial strains highly enriched in the gut but very poorly represented in matched substrate samples [29,30]. This indicates that these enriched bacteria are gut symbionts, and it will also be interesting to study them in more detail.

The presence of stable associations in the wild raises the question of why these seem to have been lost in laboratory stocks. Part of the answer may be related to the fact that association with non-colonizing bacteria can be as beneficial as with colonizing bacteria in the lab (e.g., *Acetobacter* OTU2753 versus *A*. *thailandicus*). Fly husbandry conditions in the lab normally ensure transmission of bacteria from generation to generation, even if they do not stably colonize the gut. Therefore, under laboratory conditions, there may be a loss of selective pressure for stability. This can lead to loss of the capacity to stably colonize the gut by the bacteria, either by drift or by selection, if there is a cost associated with this capacity. Alternatively, colonizing bacteria may be replaced by non-colonizing strains in the lab. The lab diet is relatively uniform and different from the natural diet; therefore, bacteria better adapted to these conditions may outcompete wild isolates [47]. Moreover, use of antifungal antimicrobials, and sometimes antibiotics, may constantly or occasionally severely disrupt bacterial communities associated with the flies that are then replaced with local bacterial strains that do not have the capacity to colonize *Drosophila*. One or combinations of these factors may, over the long periods of time that flies are kept in the lab, lead to the loss of the original microbiota. From our experience, wild bacterial isolates seem to be easily outcompeted in lab conditions and replaced by other bacteria, because we needed to carefully handle the fly stocks to keep the monoassociations with wild isolates.

Exploring the interactions between the host and its natural colonizing symbionts can uncover new phenotypes missed in laboratory experiments. Previous studies with other organisms have shown that indeed this can be the case. For instance, in the nematode *Caenorhabditis elegans*, bacteria isolated from natural habitats conferred higher fitness when compared with the standard *Escherichia coli* used in the laboratory [107,108]. Also, wild collected mice harbor a different microbiota than laboratory mice, which decreases inflammation and is protective upon infection and tumorigenesis [109]. The capacity to colonize and proliferate in the gut of *D*. *melanogaster* described in this study demonstrates different properties of lab and wild bacterial isolates. Moreover, other phenotypes associated with wild isolates may yet be identified.

The stable interaction we found between *D*. *melanogaster* and gut bacteria will be useful to address important questions in the gut microbiota field using this model system. This includes identifying and characterizing, from the host and bacteria perspective, genes required for colonization and for the control of this interaction. Moreover, it will allow understanding determinants of specificity, which are largely unknown, although adhesion and biofilm formation are important in this process [110,111]. These questions are also relevant to better understand and manipulate insect gut symbionts. The release of insects with specific gut bacteria in interventions may be useful against pests (e.g., by increasing the fitness of sterile males [112]) and against vectors of disease (e.g., by increasing resistance to pathogens [113,114]). Knowing what regulates gut stability may be important for the success of these approaches.

Our work defines a new paradigm for the association between *D*. *melanogaster* and gut bacteria, in which stable associations exist and contribute to the fitness of both partners in an ecological context. Therefore, this new conceptual and experimental framework to study gut stable symbionts will contribute to the growing field of *Drosophila*–microbe interactions.

## Materials and methods

### Wild fly collection, stocks source, and maintenance

Wild flies were collected with traps, with fallen figs as bait, placed for 24 hours under a fig tree in Oeiras, Portugal (GPS coordinates 38°41'32.1"N, 9°18'59.4"W). *D*. *melanogaster* and *D*. *simulans* males were identified according to [40]. All the material to collect and sort wild flies was sterilized prior to use.

DrosDel *w*^*1118*^ isogenic stock (*w*^*1118*^ *iso*) [33] was used as a laboratory stock, unless otherwise indicated. The female lines *D*. *melanogaster* O13 and *D*. *simulans* O13 were established from single wild females collected in 2013, and later identified to the species level. Other stocks used were *D*. *melanogaster* Canton-S (Bloomington Drosophila Stock Center at Indiana University, stock #1) and *D*. *simulans* A07 and J04 (Drosophila Species Stock Center from California University, stocks #14021–0251.260 and #14021–0251.187, respectively). Unless otherwise indicated, flies were 3–6 days old in the beginning of experiments. The age of wild-caught flies is uncontrolled.

Stocks were kept and experiments were performed at 25°C in standard *Drosophila* food composed of 1.05 L water, 80 g molasses, 22 g beet syrup, 8 g agar, 10 g soy flour, 80 g cornmeal, 18 g yeast. Food was autoclaved and cooled to 45°C before adding 30 mL of a solution containing 0.2 g of carbendazim (Sigma) and 100 g of methylparaben (Sigma) in 1 L of absolute ethanol.

### Bacterial culture

Analysis of bacteria present in the gut was performed by culture-dependent methods in order to isolate bacteria for further manipulations. From each fly, the gut (including crop, midgut, and hindgut), together with the Malpighian tubules, was dissected in Tris-HCl 50 mM, pH 7.5, and homogenized with a plastic pestle in an 1.5 mL microcentrifuge tube with 250 μL Lysogeny Broth (LB). Each sample was serially diluted (1:10 factor) and 30 μL from each dilution were plated in five different culture media: LB (GRiSP), de Man, Rogosa and Sharpe broth (MRS) (Merck), Liver Infusion Broth (Becton Dickinson), Brain heart infusion (BHI) (Sigma-Aldrich), and Mannitol (3 g of Bacto Peptone [Becton Dickinson], 5 g of Yeast Extract [Sigma-Aldrich], 25 g of D-Mannitol [Sigma-Aldrich], 1 L of Milli-Q water). Plates were incubated at 25°C for 6 days and dilutions containing 30–300 CFUs were used to count and isolate bacteria.

To analyze flies or food associated with only specific bacterial isolates, samples were plated on specific media to grow the correspondent bacteria (Mannitol for *Acetobacter* and MRS for *Leuconostoc* or *Lactobacillus*). Plates were incubated at 25°C for 4 days.

### Quantification, isolation, and identification of gut-associated bacteria

For quantification of total bacteria in each gut sample, we selected the data from the medium that presented the highest number of colonies.

For a detailed analysis, bacterial colonies were assigned in each culture medium plate, per sample, to distinct morphological types, and their number determined. Two colonies of each morphological type, per culture medium plate, per sample, were re-streaked and, after growth, colonies were picked, resuspended in 500 μL LB containing 15% glycerol (v/v), and frozen at −80°C.

To identify each bacterial isolate, a PCR was performed to amplify the 16S rRNA gene. For most samples a bacterial colony, or part of it, was directly placed in the PCR reaction tube (colony PCR). In the few cases in which amplification was unsuccessful by colony PCR, DNA extraction was performed with ZR Fungal/Bacterial DNA MiniPrep Kit (Zymo Research) according to the manufacturer's instructions. Primers used were: 27f (5′-GAGAGTTTGATCCTGGCTCAG-3′) and 1495r (5′-CTACGGCTACCTTGTTACGA-3′), with the following PCR conditions: 94°C for 4 minutes; 30 cycles of 95°C for 30 seconds, 58°C for 1 minute, and 72°C for 2 minutes; 72°C for 10 minutes. PCR products were sequenced at Source Biosciences Sequencing Center. Sequences were trimmed to 800 bp of each sequence, including V2 to V4 hypervariable regions. These sequences were aligned against a core set aligned fasta file from Greengenes [34], using PyNAST [115], and classified into OTUs according to Greengenes taxonomy [34]. Sequences that matched *Ralstonia* OTU3005, *Novosphingobium stygium* OTU2886, and *Novosphingobium* OTU2881 were removed from the analysis because they were occasionally present on negative controls for PCR.

In most cases, each morphological type corresponded to one OTU. However, three groups of bacteria had different OTUs commonly assigned to the same morphological type. Thus, these bacteria could not be distinguished within their group, based on colony morphology. These groups are composed of bacteria belonging to the *Lactobacillus* genus, the Acectobacteraceae family (*Acetobacter* and *Gluconobacter* genera), or the Enterobacteriaceae family. The frequencies of the sequenced colonies from each group are represented in Fig 3 and S3 Fig.

To determine CFUs per gut for each OTU, or group of bacteria, the data from the medium that presented the highest number of colonies was selected.

Bacterial isolates used for phenotypic analysis (*Acetobacter* OTU2753, *A*. *thailandicus*, *A*. *cibinongensis*, *L*. *pseudomesenteroides*, and all isolates used in Fig 7B and S16 Fig) were sequenced with both 27f and 1495r primers and analyzed at least from V2 to V8 hypervariable regions of the 16S rRNA sequence. Sequences were automatically edited with PhredPhrap, and consensus sequences were generated using BioEdit Sequence Alignment Editor Software. Sequences are in S1 Text and deposited in GenBank with the following accession numbers: MG808351.1, MG808350.1, MG808352.1, MG808353.1, MG808354.1, MG808355.1, MG808356.1, MG808357.1, MG808358.1, MG808359.1, MG808360.1, MG808361.1, MG808362.1, MG808363.1, MG808364.1, and MG808365.1.

The *A*. *thailandicus* isolate was initially identified as *A*. *indonesiensis* OTU2758 based on the Greengenes analysis of the V2–V4 hypervariable regions. Later, the analysis of the full 16S rRNA gene sequence matched several different *Acetobacter* OTUs with 98% identity in the Greengenes analysis. Therefore, we used BLAST to analyze the full sequence against the NCBI 16S rRNA sequences (Bacteria and Archaea) database [116]. *A*. *thailandicus* 16S rRNA gene was the best hit and was 99% identical to the sequence of this isolate [57].

### Real-time qPCR for 16S rRNA gene

DNA was extracted from dissected single guts with QIAamp DNA Micro kit (Qiagen) as described in the protocol "isolation of Genomic DNA from Tissues." To facilitate DNA extraction from gram-positive bacteria, the guts were homogenized in 180 μL of enzymatic lysis buffer with Lysozyme (from DNeasy Blood & Tissue Kit, QIAGEN) and incubated for 1 hour at 37°C before starting the protocol. DNA concentrations were determined with a NanoDrop ND-1000 Spectrophotometer. qPCR reactions were carried out in CFX384 Real-Time PCR Detection System (BioRad). For each reaction in a 384-well plate (BioRad), 6 μL of iQ SYBR Green supermix (BioRad), 0.5 μL of each primer solution at 3.6 mM, and 5 μL of diluted DNA were used. Each plate contained three technical replicates of every sample for each set of primers. Primers used to amplify the 16S rRNA gene were: 8FM (5′-AGAGTTTGATCMTGGCTCAG-3′) and Bact515R (5′-TTACCGCGGCKGCTGGCAC-3′) [117]. Primers used to amplify *Rpl32* were Rpl32 forward (5′-CCGCTTCAAGGGACAGTATC-3′) and Rpl32 reverse (5′-CAATCTCCTTGCGCTTCTTG-3′). The thermal cycling protocol for the amplification was initially 50°C for 2 minutes, and denaturation for 10 minutes at 95°C, followed by 40 cycles of 30 seconds at 95°C, 1 minute at 59°C and 30 seconds at 72°C. Melting curves were analyzed to confirm specificity of amplified products. Ct values for manual threshold of 10 were obtained using the program SDS 2.4 or with Bio-Rad CFX Manager, with default threshold settings. Gene levels of 16S rRNA were calculated relative to the Day 0 sample with the Pfaffl method [118] using *Drosophila Rpl32* as a reference gene.

### Generation of axenic and monoassociated flies

To develop axenic flies, embryos were sterilized with 2% sodium hypochlorite during 10 minutes, followed by 70% ethanol during 5 minutes, and washed with sterile water. Embryos were placed in sterilized food vials and maintained in axenic conditions or monoassociated with 40 μL of overnight bacterial culture of specific isolates. Monoassociated stocks were kept at 25°C and flipped every 20 days, using sterile gloves. We waited at least two generations in monoassociation before performing experiments.

### Analysis of bacterial stability in the gut

The gut stability protocol in vials was based on placing a single fly per vial, with a food surface of 3.8 cm^2^, and changing it twice a day to new vials. The stability protocol in cages was based on placing a single fly per cage with six petri dishes, with a total fly food surface of 382 cm^2^, and changing them daily. Bacterial levels were analyzed in single guts.

To analyze the gut region where stable bacteria are present, individual guts were dissected into five different regions: crop, anterior midgut, mid midgut, posterior midgut, and hindgut. The proventriculus was included in the anterior midgut sample. Each gut region from a single fly was homogenized, plated, and quantified, as described above.

### Analysis of bacterial proliferation in the gut

The proliferation assay was based on providing an inoculum of bacteria to axenic male flies for 6 hours and measuring gut bacterial levels, by plating, immediately at the end of this period (time 0 hours), and 24 hours later. Bacteria were grown in the Mannitol (*Acetobacter*) or MRS (*Leuconostoc* or *Lactobacillus*) liquid media in a shaker at 28°C overnight. Bacterial concentrations (cell/mL) were calculated based on OD600 using a spectrophotometer (SmartSpec 3000 from Biorad), using the formula OD1 = 5 × 10^8^ cell/mL. The inoculum was provided in vials by adding 180 μL of bacterial solution in 2.5% sucrose to a round filter paper placed on top of the fly food. After the inoculation period, flies were placed singly in cages or in bottles (food surface: 382 cm^2^ and 28 cm^2^, respectively) for 24 hours. Bacterial levels were analyzed in single guts by plating.

To confirm that the 24 hours data corresponded to bacteria growing in the gut and not bacteria growing on the fly food and in transit, we added an axenic fly to the cage or bottle at time 0, in some experiments. Bacterial levels in the gut of these chaser flies were determined at time 24 hours, simultaneously with the cohabiting experimental fly. Chaser flies and experimental flies were distinguished by marking one or the other, in different experimental replicates, with a dot in the wing with a permanent pen.

### Analysis of bacterial proliferation on fly food

To analyze bacterial growth on food from bacteria associated with flies, conventionally reared 3–6-day-old males were placed singly in vials for 24 hours, in order to contaminate the food with bacteria. After that period, flies were discarded and vials were incubated for 9 days at 25°C. Bacterial levels were determined after discarding the flies (Day 1) and after the 9 days of incubation (Day 10). Vials that never contained flies before were used as control vials and incubated also for 9 days (Day 10 control). Of the top layer of food, 2.9 g were homogenized in 10 mL LB. This homogenate was plated in the five different media.

To analyze growth of *Acetobacter* species on the fly food, 3–6-day-old males monoassociated with the different *Acetobacter* were singly placed in vials with 4 mL of fly food for 16 hours. After that period, males were discarded and bacterial levels were assessed at that time point (Day 0) and after 1 or 5 days of incubating the vials at 25°C. All the food from the vial was homogenized in 4 mL LB. Mannitol plates were incubated at 25°C for 4 days.

### Fitness parameters determination

To determine fitness parameters in monoassociated stocks (S13 Fig), one virgin female and three 0–3-day-old males were placed per vial for 3 days and then discarded. Time to pupariation and to adulthood was daily assessed, as well as total number of pupae and adults.

To analyze fitness parameters of flies in a changing environment (Fig 6, S14 Fig), axenic 1–3-day-old females and males were in contact for 6 hours with an inoculum of 10^5^ CFU/μL *Acetobacter* OTU2753, *A*. *thailandicus*, or with sterile Mannitol. After this period, one female and two males were placed per cage for 10 days. Each cage contained six bottles with food that were changed every day (total food surface of 170 cm^2^). Single gut bacterial loads were analyzed in females 0 hours and 10 days postinoculation and in males 10 days after inoculation.

From each cage, all six bottles were daily collected, the number of eggs was counted, and bottles were kept to daily assess adult emergence (Fertility of G0 and Development of G1). Transmission of bacteria to the food was analyzed in bottles without eggs at days 1, 3, 5, 7, and 9. The food surface was washed with 1,000 μL of Mannitol, and 100 μL of this suspension was plated in Mannitol. As a control, food from axenic flies was also tested at Days 1 and 9, and no bacteria were detected.

To analyze fertility of G1, bottles from day 9 and 10 from each condition were used to collect flies. One female and one male from the same condition were placed per vial and flipped to new ones every other day, during 10 days. Adult emergence was daily assessed to determine total number of adults (Fertility of G1).

To analyze if the benefit of *A*. *thailandicus* was dependent on the association with either parent, we compared the four possible pairs of males and females from an axenic stock and a stock monoassociated with *A*. *thailandicus* (Fig 6H, S15 Fig). We placed one female and one male, both 1–2 days old, per vial, and flies were passed to new vials every other day during 10 days. We also tested a condition in which 30 μL of an overnight *A*. *thailandicus* culture was added to the progeny of axenic parents immediately after emptying it of parents. We daily assessed developmental time to pupariation and adulthood.

To analyze fitness parameters conferred by different natural bacterial isolates (Fig 7B, S16 Fig), 50 sterilized eggs were placed per vial and inoculated with 40 μL of an overnight bacterial culture. All isolates were grown at 28°C in Mannitol, except *L*. *brevis*, *L*. *paraplantarum*, and *L*. *pseudomesenteroides*, which were grown in MRS. As controls, we analyzed sterilized eggs associated with only Mannitol or MRS, or with no medium added. Number of adults (G0) and developmental time to adulthood (G0) were assessed. One male and female of the first adults emerging from each condition were placed per vial and flipped every other day during 8 or 10 days. Adult emergence was daily assessed to determine total number of adults (Fertility of G0).

To analyze the impact of *A*. *thailandicus* on fitness parameters in sterile figs homogenate (Fig 7C–7G, S18A–S18I Fig), 30 or 50 sterilized eggs were placed per vial and inoculated with 40 μL of an overnight culture of *A*. *thailandicus*, *Acetobacter* OTU2753, or sterile Mannitol. Adult emergence was daily assessed. For the analysis of this G0 fertility, one male and one female adult that emerged from these vials were placed per vial and flipped every other day during 8 or 10 days. Adult emergence was daily assessed to determine total number of adults (Fertility of G0). Bacteria presence was confirmed on the last vials of egg laying, by resuspending the food in 1 mL of PBS 1× and plating it in Mannitol. Both bacteria were present. The fig food homogenate was produced with 300 mL homogenized commercial frozen figs, 600 mL water, and 4.8 g agar. After autoclave, food was poured into each vial in sterile conditions, inside a laminar flow hood.

To analyze the fitness impact of *A*. *thailandicus* in fresh figs, we collected these at the same location where the wild flies were collected. Figs were cut in quarters and placed in vials with sterilized agar (0.8% agar in water) at the bottom to fix the fig. Thirty sterilized embryos were placed on the top of these figs and inoculated with 40 μL of an overnight culture of *A*. *thailandicus* or sterile Mannitol. Quarters originated from the same fig were distributed to the two conditions. Adult emergence was daily assessed. As a control, figs without the addition of eggs were kept, and no flies emerged from those ones. Also, all flies that emerged from the experimental conditions had white eyes, confirming that they developed from the sterilized eggs and not from a possible contamination with wild flies present in the figs.

### Imaging

All the imaging analysis was performed in flies monoassociated with *A*. *thailandicus*.

FISH was performed in flies at Day 0 and Day 5 of the stability protocol in cages, with daily changed food. Flies were dissected in cold PBS 1×, and guts were fixed and permeabilized in 4% paraformaldehyde (Merck) and 1% Triton-X-100 (Sigma) in PBS 1× (PBST) for 90 minutes at 4°C. Samples were washed once for 15 minutes with PBST and twice for 5 minutes with phosphate-buffered saline 1× (PBS) at room temperature (RT). Dehydration was performed in an ascending series of ethanol concentrations (50%, 80%, and 100%) for 3 minutes each, rinsed twice in PBS 1×, and incubated overnight at 4°C in 6% hydrogen peroxide (Sigma) and 80% ethanol. Samples were then washed twice for 5 minutes in PBS 1×, prehybridized for 30 minutes without the probe, and hybridized overnight at 37°C with the universal probe for 16S rRNA gene EUB338 (5′-Cy3-GCTGCCTCCCGTAGGAGT-3′) [119] (Sigma) in hybridization buffer (5× SSC [ThermoFisher], 50% formamide [Sigma], 20mM Tris-HCl, 8% Dextran Sulfate, 0.1% SDS [Sigma], and 200 ng/mL of probe). Post-hybridization washes were performed at 55°C. Samples were rinsed once in 2× SSC, twice for 15 minutes with 20% formamide in 0.5× SSC, once for 15 minutes in 0.5× SSC, and once for 15 minutes with 2× SSC. Samples were counterstained with Hoechst for 20 minutes in PBS 1× to stain DNA. After washing in 1× PBS, guts were mounted in Vectashield Mounting Medium (Vector laboratories).

To observe live and dead bacterial cells in the gut, we used males after 9 days of the stability protocol. Live and dead bacteria were stained with SYTO9 and propidium iodide (PI), respectively, using the Invitrogen LIVE/DEAD BacLight Bacterial Viability Kit (L7012), and the protocol was adapted from [27]. Males were fed with a 20 μL mix of 2.5% sucrose, 1.5 μL of SYTO9, and 1.5 μL of PI, and after 1 hour, guts were dissected and counterstained with Hoechst (Sigma) for 10 minutes in 1× PBS. Samples were washed in 0.9% NaCl, mounted in Vectashield Mounting Medium, and immediately observed.

Confocal images were taken with Leica SP5 and processed in Fiji [120].

For transmission electron microscopy (TEM), flies were dissected 5 days after the stability protocol in cages and the guts were fixed with 2% formaldehyde (EMS) and 2.5% glutaraldehyde (Polysciences) in 0.1 M phosphate buffer (PB) (pH 7.4) for 90 minutes. Three washes were performed for 10 minutes each in 0.1 M PB, and samples were embedded for support and orientation in 2% low melting point agarose (OmniPur) and stained with toluidine blue. After solidifying, guts were postfixed with 1% osmium tetroxide (EMS) in 0.1 M PB on ice for 1 hour. After three washes in dH_2_O for 10 minutes each, samples were en bloc stained with 0.5% uranyl acetate in dH2O for 1 hour in the dark. Dehydration was performed with ascending series of ethanol concentrations (30%, 50%, 75%, 90%, and 100%). Infiltration was performed with ascending concentrations of Embed-812 epoxy resin (EMS) (25%, 50%, and 75%, for 1 hour each, and 100% overnight) and cured overnight at 60°C. Proventriculus and crop were cut at 70 nm for TEM using a Leica UC7/FC7 Ultramicrotome. Ultrathin sections were post-stained with 0.5% uranyl acetate and Reynolds lead citrate for 5 minutes each and observed in a Hitachi H-7650 electron microscope operating at 100 KeV.

### Statistical analysis

The statistical analysis was performed in R [121] and graphs were generated using the package ggplot2 [122] and GraphPad. The script of all the analyses is provided in S2 Text, where details can be found.

Bacterial levels; number of eggs, pupae, and adults; and time to pupariation and adulthood were analyzed using linear models, or linear mixed-effect models (package *lme4* [123]) if there were random factors. Significance of interactions between factors was tested by comparing models fitting the data with and without the interactions using analysis of variance (ANOVA). Models were simplified when interactions were not significant. Pairwise comparisons of the estimates from fitted models were analyzed using *lmerTest* [124], *lsmeans* [125], and *multcomp* [126] packages.

Time course analysis of bacterial stability in cages was performed fitting a nonlinear least-squares model with the parameters of an exponential decay curve. Model simplification was achieved through ANOVA and Akaike information criterion (AIC) of fitted models.

Bacterial levels in flies in the changing environment cage assay were analyzed with the nonparametric Mann–Whitney test because some data points were high and not estimated precisely.

Bacteria transmission to bottles in the changing environment cage assay was analyzed with a generalized linear mixed-effects (*lme4* package) with a binomial distribution.

Independence of *Lactobacillus* and *Leuconostoc*, or different Acetobactereaceae, presence in wild-caught flies was tested with the Pearson’s chi-squared test.

Correlation between developmental time and fertility of flies that developed associated with different bacteria was tested through the Pearson correlation of the means of these parameters.

## Supporting information

S1 Fig Wild-caught *Drosophila melanogaster* have a stable gut microbiota. Single 3–6-day-old *w*^*1118*^ *iso* males were kept in the same vial during 10 days (A) or exposed to a stability protocol by being passed to new vials twice a day (A, B). (A) Five individuals were analyzed at each day, and total number of CFUs per gut was determined by bacterial plating. Bacterial levels increase in the flies maintained in the same vials and decrease in the flies flipped to new vials twice a day (lmm, *p* < 0.001 for both). Supporting data can be found in S1 and S2 Data. (B) Relative amount of 16S rRNA bacterial gene was measured by quantitative-PCR in five individual guts from each day, using the host gene *Rpl32* as a reference gene. Relative amount of 16S rRNA gene decreases between days (lmm, *p* < 0.001). Supporting data can be found in S3 Data. (C, D) Bacterial levels from wild-caught flies on the day of collection (Day 0) and after 5, 10, or 20 days of the stability protocol. Bacterial levels in the flies significantly decrease with time (lmm, *p* = 0.004). Supporting data can be found in S5 Data. Each dot represents an individual gut and the lines represent medians. Statistical analyses were performed together with replicate experiments shown in Fig 1. CFU, colony-forming unit; lmm, linear mixed model; *w*^*1118*^ *iso*, *w*^*1118*^ DrosDel isogenic strain.
(TIF)

S2 Fig Higher diversity of gut bacterial communities in wild-caught *Drosophila melanogaster*. Accumulation curve of the different bacterial OTUs present in wild-caught and laboratory flies before (Day 0) and after (Day 10) being exposed to the stability protocol. Supporting data can be found in S6 Data. OTU, operational taxonomic unit.
(TIF)

S3 Fig Total levels and diversity of Enterobacteriaceae in wild-caught *Drosophila melanogaster*. (A) Levels of Enterobacteriaceae in the gut of wild-caught flies before (Day 0) and after 10 days of the stability protocol (Day 10). Each dot represents one gut and lines represent medians. Levels of Enterobacteriaceae decrease between days (lm, *p* = 0.01). (B) Frequencies of sequenced colonies of Enterobacteriaceae for Day 0 and Day 10, represented as several in Fig 2. Numbers on the top of the bars correspond to the number of flies carrying that specific OTU, from a total of 10 flies. Supporting data can be found in S6 Data. lm, linear model; OTU, operational taxonomic unit.
(TIF)

S4 Fig *Leuconostoc pseudomesenteroides* stably associates with the gut of wild *Drosophila melanogaster*. Total *L*. *pseudomesenteroides* levels in the gut of wild-caught flies on the day of collection (Day 0) and after 10 days of the stability protocol (Day 10). Levels of *L*. *pseudomesenteroides* are not significantly different between days (lm, *p* = 0.372). Each dot represents one gut and the line represents the median. Supporting data can be found in S6 Data. lm, linear model.
(TIF)

S5 Fig *Acetobacter thailandicus* and *A*. *cibinongensis* stably colonize the gut of *Drosophila melanogaster*. Single 3–6-day-old *w*^*1118*^ *iso* males from monoassociated stocks with *Acetobacter* OTU2753 (A, E), *A*. *cibinongensis* OTU2755 (B, F), *A*. *thailandicus* (C, G), or *Leuconostoc pseudomesenteroides* (D, H) were exposed to the stability protocol for 10 days in vials (A–H) or 5 days in cages (E–H). Number of CFUs in individual guts was assessed by plating before and after 5 or 10 days of the stability protocol. Ten flies were analyzed for each condition. *Acetobacter* OTU2753, *A*. *cibinongensis*, and *A*. *thailandicus* levels decrease between Day 0 and Day 10 in vials (lmm, *p* < 0.001 for all), but *L*. *pseudomesenteroides* levels do not significantly change (*p* = 0.96). (I) Data from Fig 4B–4E were fitted to an exponential decay model that estimates the exponential decay rate, which corresponds to the rate of bacterial loss from the gut, and an asymptote, which corresponds to the levels at which the bacteria levels tend to stabilize after this loss. The rate of decay is the same for all the bacteria, but there are differences between the asymptotes of all bacteria (contrasts of nonlinear least-squares model estimates, *p* < 0.014), except between *Acetobacter* OTU2753 and *L*. *pseudomesenteroides* (*p* = 0.116). Supporting data can be found in S7 Data. CFU, colony-forming unit; lmm, linear mixed model fit; *w*^*1118*^ *iso*, *w*^*1118*^ DrosDel isogenic strain.
(TIF)

S6 Fig *Acetobacter thailandicus* stable population persists in the crop, crop duct, and proventriculus of *Drosophila melanogaster*. (A, B) Number of CFUs in each gut compartment from *w*^*1118*^ *iso* males monoassociated with *A*. *thailandicus* before (A) and after (B) 5 days of the stability protocol. Each dot represents one gut or one gut fragment and lines represent medians. Supporting data can be found in S8 Data. (C, D) Fluorescent in situ hybridization with Cy3 labeled Bacteria 16S rRNA universal probe EUB338 for bacteria in the gut of males monoassociated with *A*. *thailandicus* at Day 0 (C) and Day 5 (D) of the stability protocol. On Day 0, bacteria are found in all gut compartments, while at Day 5, bacteria persist in the crop, crop duct, and proventriculos. On (D), arrows point to bacteria and arrowheads to autofluorescence. Scale bar corresponds to 50 μm. CFU, colony-forming unit; Cy3, cyanine dye 3; *w*^*1118*^ *iso*, *w1118* DrosDel isogenic strain.
(TIF)

S7 Fig Fluorescent in situ hybridization of *Acetobacter thailandicus* in *Drosophila melanogaster* females and males. Fluorescent in situ hybridization with Cy3 labeled Bacteria 16S rRNA universal probe EUB338 for bacteria was performed in flies monoassociated with *A*. *thailandicus* 5 days after the stability protocol. Similar to males, bacteria persist in the anterior part of the crop (A), crop duct (B), and proventriculus (C) of females. (D, E) In a few cases, bacteria were also found in the anterior midgut (D) or in the rectum of flies (E). DNA was stained with Hoechst. Scale bar corresponds to 50 μm in (A, E) and to 10 μm in (B–D). Cy3, cyanine 3 dye.
(TIF)

S8 Fig Negative control for fluorescent in situ hybridization. Fluorescent in situ hybridization with Cy3 labeled Bacteria 16S rRNA universal probe EUB338for bacteria was performed in axenic flies as a control. Full gut (A), anterior part of the crop (B), crop duct (C), and proventriculus (D). Arrowheads point to autofluorescence of chitin and food. DNA was stained with Hoechst. Scale bar corresponds to 500 μm in (A) and to 10 μm in B–D. Cy3, cyanine 3 dye.
(TIF)

S9 Fig Clusters of *Acetobacter thailandicus* in the crop and proventriculus. Clusters of bacterial cells (b) near the chitin folds of the crop (A, B) and in the lumen of the proventriculus (C–I) observed by scanning electron microscopy. Some cells present membrane invaginations (mi) and seem to be dividing. Cells seem to be attached to each other by external appendages, such as fimbriae (f). Extracellular vesicles are found between cells (v). Scale bar corresponds to 10 μm in (C, E), 2 μm in (A, B, D), and 1 μm in (F, G, H, I). (D) is a magnification of (C). (F) and (G) are magnifications of (E). b, bacterial cell; f, fimbriae; mi, membrane invagination; v, vesicles.
(TIF)

S10 Fig *Acetobacter thailandicus*, *A*. *cibinongensis*, and *Lactobacillus brevis* proliferate in the gut of *Drosophila melanogaster*. Three- to six-day-old axenic *w*^*1118*^ *iso* males were inoculated for 6 hours with different concentrations of *Acetobacter* OTU2753 (A, E), *A*. *cibinongensis* OTU2755 (B, F), *A*. *thailandicus* (C, G), *Leuconostoc pseudomesenteroides* (D), *Lactobacillus paraplantarum* (H, I), and *L*. *brevis* (J). Bacterial levels were assessed by plating 0 and 24 hours postinoculation. During this period, males were singly placed in cages. In (G), axenic chaser males were placed in cages together with males inoculated with *A*. *thailandicus*. At 24 hours, bacterial levels were assessed in both males. Bacterial levels between 0 and 24 hours decrease in flies inoculated with *Acetobacter* OTU2753 (lmm, *p* < 0.001), increase in flies inoculated with *A*. *cibinongensis*, *A*. *thailandicus*, and *L*. *brevis* (lmm, *p* = 0.024, *p* < 0.001, and *p* = 0.046, respectively) and do not significantly change in flies inoculated with *L*. *pseudomesenteroides* and *L*. *paraplantarum* (lmm, *p* = 0.158 and *p* = 0.65, respectively). Four to five males were used per condition, except in (B), in which three males were used at one time point and in (D), in which two males were used on the inoculation 10^4^ CFU/μL. Each dot represents one gut and lines represent medians. Statistical analyses were performed together with replicate experiments shown in Fig 5B–5G. Supporting data can be found in S9 and S10 Data. CFU, colony-forming unit; lmm, linear mixed model; *w*^*1118*^ *iso*, *w*^*1118*^ DrosDel isogenic strain.
(TIF)

S11 Fig *Acetobacter* species grow on the fly food media. Single 3–6-day-old *w*^*1118*^ *iso* males from a monoassociated stock with *Acetobacter* OTU2753 (A, D), *A*. *thailandicus* (B, E), or *A*. *cibinongensis* (C, F) were placed per vial for a period of 16 hours and then discarded. Bacterial levels on the food were determined by plating after discarding the flies (Day 0) and after 1 or 5 days of incubating these vials. Levels of *Acetobacter* on the food increase for all conditions between Day 0 and Day 5 (lmm, *p* < 0.001). Five vials were used per condition. Each dot represents the bacterial levels on the food of one vial and lines represent medians. Supporting data can be found in S11 Data. lmm, linear mixed model; *w*^*1118*^ *iso*, *w*^*1118*^ DrosDel isogenic strain.
(TIF)

S12 Fig *Acetobacter thailandicus* proliferates in the gut of *Drosophila melanogaster* and not in *D*. *simulans*. (A, B) Optimization of proliferation protocol in bottles. Axenic 3–6-day-old *w*^*1118*^ *iso* were inoculated for 6 hours with different concentrations of *Acetobacter* OTU2753 (A) or *A*. *thailandicus* (B). Bacterial levels were assessed 0 and 24 hours postinoculation. During this period, males were singly placed in bottles (food surface of 28 cm^2^) together with an axenic chaser male, from which bacterial levels were also assessed at 24 hours. Levels of *Acetobacter* OTU2753 decrease between days (lmm, *p* < 0.001). Levels of *A*. *thailandicus* increase when flies are inoculated with the lowest concentration (*p* < 0.001) and are maintained when flies are inoculated with the highest concentration (*p* = 0.426). Supporting data can be found in S12 Data. (C–E) Axenic 3–6-day-old *D*. *melanogaster* or *D*. *simulans* males were inoculated for 6 hours with 10^3^ CFU/μL (C, D) or 10^4^ CFU/μL (E) of *A*. *thailandicus*. Bacterial levels were assessed 0 and 24 hours postinoculation. During this period, males were singly placed in bottles. Three different genetic backgrounds of *D*. *melanogaster* (*w*^*1118*^ *iso*, *D*. *mel*. O13, and Canton-S) and of *D*. *simulans* (*D*. *sim*. J04, *D*. *sim*. O13, and *D*. *sim*. A07) were used. Bacterial levels in the gut increase in *D*. *melanogaster* and decrease in *D*. *simulans* (*p* < 0.001). Supporting data can be found in S13 Data. Five individuals were analyzed for each condition, per experimental replicate, and total number of CFUs per gut determined by plating. Each dot represents one gut and the line represents medians. Statistical analyses were performed together with the replicate experiment shown in Fig 5H. CFU, colony-forming unit; lmm, linear mixed model; *w*^*1118*^ *iso*, *w*^*1118*^ DrosDel isogenic strain.
(TIF)

S13 Fig Flies monoassociated with *Acetobacter* develop faster and are more fertile than axenic flies in a constant environment. Total number of pupae (A), total number of adults (B), developmental time to pupariation (C), and developmental time to adulthood (D) were analyzed in flies from a monoassociated stock with *Acetobacter* OTU2753 or *A*. *thailandicus*, or in axenic flies. One female and three males from each condition were placed per vial for 3 days and then discarded. Number of pupae or emerged adults was daily assessed. Ten vials were used per condition. Flies monoassociated with either *Acetobacter* species develop faster and have higher fertility than axenic flies (lm, *p* < 0.001). (A, B) Each dot represents the total progeny of one female. Supporting data can be found in S14 Data. lm, linear model.
(TIF)

S14 Fig *Acetobacter thailandicus* stable association with *Drosophila melanogaster* is mutualistic. Axenic 1–3-day-old *w*^*1118*^ *iso* males and females (G0) were in contact with an inoculum of 10^5^ CFU/μL of *Acetobacter* OTU2753 or *A*. *thailandicus* for 6 hours. Two males and one female were placed per cage, with five cages for each condition, during 10 days, with daily changed food. (A) Bacterial levels in single guts of females 0 days and 10 days postinoculation and in males 10 days postinoculation, analyzed by plating. Bacterial levels between the two time points increased in females inoculated with *A*. *thailandicus* and decreased in females inoculated with *Acetobacter* OTU2753 (Mann–Whitney test, *p* < 0.001 and *p* = 0.048, respectively). Supporting data can be found in S15 Data. (B) Presence of bacteria on the food collected from cages at days 1, 3, 5, 7, and 9 of the protocol, analyzed by plating. Filled rectangles represent presence of bacteria. *A*. *thailandicus* is transmitted to the food with higher frequency than *Acetobacter* OTU2753 (glm-binomial, *p* < 0.001). Supporting data can be found in S16 Data. (C–F) Effect of bacterial association on the fitness of *D*. *melanogaster*. Total number of eggs laid by flies inoculated with different *Acetobacte*r (C) and total number of adults that emerged from these eggs (D). Total number of eggs or adults is not different between conditions (lmm, *p* > 0.484). (E) Developmental time to adulthood of the progeny (G1) of flies inoculated with different *Acetobacter*. Developmental time to adulthood is faster in progeny from flies inoculated with *A*. *thailandicus* than in progeny from flies inoculated with *Acetobacter* OTU2753 (lmm, *p* < 0.001). Supporting data can be found in S17 and S18 Data. (F) Fertility of G1. Two males and one female of G1 were placed per vial and flipped every other day for 10 days. Five couples were made per condition. Total number of emerged adults was analyzed. Fertility is higher in progeny from flies inoculated with *A*. *thailandicus* than in progeny from flies inoculated with *Acetobacter* OTU2753 (lmm, *p* < 0.001). Supporting data can be found in S19 Data. Statistical analyses were performed together with replicate experiments shown in Fig 6B–6G. CFU, colony-forming unit; glm-binomial, generalized linear model with binomial distribution; lmm, linear mixed model; *w*^*1118*^ *iso*, *w*^*1118*^ DrosDel isogenic strain.
(TIF)

S15 Fig Both parents transmit the beneficial effect of *Acetobacter thailandicus* to their progeny. Combinations of one male and one female 1–2 days old *w*^*1118*^ *iso*, either axenic or monoassociated with *A*. *thailandicus* (Bact.), were placed in vials and flipped every other day for 10 days. To one set of vials with axenic parents, *A*. *thailandicus* was added on the eggs after passing the parents. Ten couples were made per condition. Developmental time to pupariation (A, E), to adulthood (C), total number of pupae (B, F), and total number of adults (D, G) was assessed. (A–D) correspond to one experimental replicate and (E–G) correspond to another experimental replicate, together with data from Fig 6H. Progeny from couples in which either or both parents are monoassociated and progeny from axenic flies in which *A*. *thailandicus* culture is added on the eggs develop faster than progeny from axenic flies (lmm, *p* < 0.001, for all these comparisons). Total number of progeny (pupae or adults) from couples in which either or both parents are monoassociated with *A*. *thailandicus* is higher than in progeny from axenic flies (lmm, *p* < 0.001). (B, D, F, G) Each dot represents the total progeny of one female. Statistical analyses were performed together with replicate experiment shown in Fig 6H. Supporting data can be found in S20 Data. Bact., *A*. *thailandicus*; lmm, linear mixed model; *w*^*1118*^ *iso*, *w*^*1118*^ DrosDel isogenic strain.
(TIF)

S16 Fig Different bacterial species have different impact on host developmental time and fertility. Fifty *w*^*1118*^ *iso* eggs were associated with different bacteria isolated from the gut of wild-caught *Drosophila melanogaster*. As controls, axenic eggs that had no treatment (GF) or in which sterile media were added (GF MRS and GF Mannitol) were used. Ten vials were used for each condition. Total number of emerged adults (A, B) and their developmental time to adulthood was daily assessed (C, D). Number of emerged adults is not significantly different between conditions (lmm, *p* > 0.282 for all pairwise comparisons). Flies from eggs associated with *Acetobacter thailandicus* developed faster than from axenic eggs or eggs associated with 11 out of the other 15 bacteria (lmm, *p* < 0.038 for these pairwise comparisons). Supporting data can be found in S21 Data. (E, F) Fertility of G0 was assessed. Two males and one female that developed in the presence of different bacteria (G0) were placed per vial and flipped every other day for 8 (E) or 10 (F) days. Five couples were made per condition. Total number of emerged adults was analyzed. Flies associated with *A*. *thailandicus* are more fertile than axenic flies or flies associated with 11 out of the other 15 bacteria (lmm, *p* < 0.018). Supporting data can be found in S22 Data. (A, C, E) and (B, D, F) correspond to two experimental replicates. Correlation between developmental time and fertility is represented in Fig 7B. Each dot represents the total progeny of one female (A, B, E, F) and the size of the circle represents the mean number of adults that emerged per day (C, D). Statistical groups of significance for C, D, E, F are shown in S17 Fig. lmm, linear mixed model; GF, germ free (axenic); MRS, de Man, Rogosa and Shrape broth.
(TIF)

S17 Fig Statistical groups of significance for developmental time and fertility of flies associated with different bacterial isolates. Developmental time to adulthood (A) and fertility (B) of flies associated with different bacterial isolates from S16 Fig were analyzed with Tukey’s pairwise comparisons on the lmm estimates. Statistical groups of significance were generated with *cld* function in R. Groups with the same letter are not significantly different from each other. Supporting data can be found in S21 and S22 Data. lmm, linear mixed model.
(TIF)

S18 Fig *Acetobacter thailandicus* is beneficial for *Drosophila melanogaster* in a natural food source. (A–F) Thirty axenic *w*^*1118*^ *iso* eggs were placed in vials containing sterilized fig homogenate. *A*. *thailandicus*, *Acetobacter* OTU2753, or sterile culture media were added on the top of the eggs. Four to ten vials were used per condition. Total number of adults that emerged (A–C) and developmental time to adulthood (D–F) were determined. More eggs inoculated with *A*. *thailandicus* and *Acetobacter* OTU2753 developed to adulthood, and faster than axenic eggs (lmm, *p* < 0.001 for both comparisons). Supporting data can be found in S23 Data. (G–I) Progeny of flies developed in fig homogenate with and without the addition of *Acetobacter* species. One male and one female were collected from G0 of each condition and placed per vial containing fig homogenate for 10 days, with vials flipped every other day. *A*. *thailandicus* and *Acetobacter* OTU2753 conditions have 10 replicates, but only 1 to 9 replicates from axenic eggs were possible to perform. Flies that were inoculated with *A*. *thailandicus* had higher progeny numbers than flies inoculated with *Acetobacter* OTU2753 or sterile media (lmm, *p* < 0.001). Supporting data can be found in S24 Data. (J, K) Fifty axenic *w*^*1118*^ *iso* eggs were placed in vials containing freshly collected nonsterile figs. *A*. *thailandicus* culture or sterile media (Control) was added on the top of the eggs. The total number of adults that emerged (J) and their developmental time to adulthood (K) were analyzed. Ten vials were analyzed per condition. There were more adults emerging from vials inoculated with *A*. *thailandicus* (lmm, *p* = 0.010). Developmental time to adulthood was not significantly different in this experimental replicate but was faster in eggs inoculated with *A*. *thailandicus* in the other replicate represented in Fig 7J (lmm, *p* = 0.557 and *p* < 0.001, respectively). Supporting data can be found in S25 Data. Statistical analyses were performed together with replicate experiments shown in Fig 7D–7J. lmm, linear mixed model; *w*^*1118*^ *iso*, *w*^*1118*^ DrosDel isogenic strain.
(TIF)

S1 Text Sequences of the full 16S rRNA gene of the bacteria used in the phenotypic assays. Sequence obtained by amplifying the gene with the primers 27F and 1495r. Code corresponds to code of laboratory isolate. Results of analysis on Greengenes, and of the BLAST analysis against the NCBI 16S rRNA sequences (Bacteria and Archaea) database are also shown.
(DOCX)

S2 Text R script for data analysis. Text is in R Markdown format.
(RMD)

S1 Data Bacterial levels from *w*^*1118*^ *iso* before and after 10 days in the same vial. Bacterial numbers (CFUs) calculated per gut from each culture media used (BHI, LB, MRS, Mannitol, or Liver) at Day 0 or Day 10 of the protocol. Data for Fig 1A and S1A Fig. BHI, Brain heart infusion; CFU, colony-forming unit; LB, Lysogeny Broth; MRS, de Man, Rogosa and Sharpe broth; *w*^*1118*^ *iso*, *w*^*1118*^ DrosDel isogenic strain.
(CSV)

S2 Data Bacterial levels from *w*^*1118*^ *iso* before and after 10 days of being flipped to new vials twice a day. Bacterial numbers (CFUs) calculated per gut from each culture media used (BHI, LB, MRS, Mannitol, or Liver) at Day 0 or Day 10 of the protocol. Data for Fig 1B and S1A Fig. BHI, Brain heart infusion; CFU, colony-forming unit; LB, Lysogeny Broth; MRS, de Man, Rogosa and Sharpe broth; *w*^*1118*^ *iso*, *w*^*1118*^ DrosDel isogenic strain.
(CSV)

S3 Data Relative 16S rRNA copy number *w*^*1118*^ *iso* before and 10 days after being flipped to new vials twice a day. Data for Fig 1C and S1B Fig. *w*^*1118*^ *iso*, *w*^*1118*^ DrosDel isogenic strain.
(CSV)

S4 Data Bacterial levels on the food after inoculating the food with flies. Bacterial numbers calculated per food vial from each culture media used (BHI, LB, MRS, Mannitol, or Liver) 1 or 10 days after placing one fly. Data for Fig 1D. BHI, Brain heart infusion; LB, Lysogeny Broth; MRS, de Man, Rogosa and Sharpe broth; *w*^*1118*^ *iso*, *w*^*1118*^ DrosDel isogenic strain.
(CSV)

S5 Data Bacterial levels from wild-caught flies before and 5, 10, or 20 days after being flipped to new vials twice a day. Bacterial numbers (CFUs) calculated per gut from each culture media used (BHI, LB, MRS, Mannitol, or Liver) at day 0, 5, 10, or 20 of the protocol. Data for Fig 1E, S1C and S1D Fig. BHI, Brain heart infusion; CFU, colony-forming unit; LB, Lysogeny Broth; MRS, de Man, Rogosa and Sharpe broth.
(CSV)

S6 Data Database for bacterial isolates that were sequenced and classified. Source—sample origin. Day—day of the stability protocol. Media and Dilution—culture media and respective dilution from where colonies were isolated. Fly—fly individual identity. Cfu_plate and Cfu_gut—number of colonies of each morphological type per plate and calculated per gut. morphotype—morphological type for one medium and one dilution. There is no correspondence with the same morphotype number in different media or flies. Bact_Code—code of laboratory isolate. greengenes_tax_string—list of taxonomic assignment according to Greengenes taxonomy. greengenes_prokMSA_id—identifier for the nearest neighbor sequence in the Greengenes database. greengenes_Simrank_id—percent of 7mers shared between the query sequence and the nearest neighbor sequence. greengenes_DNAML_id—identity between the query and the nearest neighbor sequences. greengenes_DNAML_columns—number of bases compared between the query and the nearest neighbor sequences. sequence—16S rRNA gene partial sequence. fly_ID—concatenation of Source, Day, and Fly information. Unique_morpho—concatenation of Source, Day, Fly, Media, and morphotype information. Data for Fig 2, Fig 3, S2, S3 and S4 Figs.
(CSV)

S7 Data Bacterial levels in *w*^*1118*^ *iso* monoassociated with different bacteria before and after being exposed to the stability protocol in vials and in cages. Data for Fig 4B–4E, S5A–S5I Fig. *w*^*1118*^ *iso*, *w*^*1118*^ DrosDel isogenic strain.
(CSV)

S8 Data Stability of *Acetobacter thailandicus* in monoassociated *w*^*1118*^ *iso* with in different gut regions. Data for Fig 4G and 4H and S6A and S6B Fig. *w*^*1118*^ *iso*, *w*^*1118*^ DrosDel isogenic strain.
(CSV)

S9 Data Proliferation of different *Acetobacter* species and *Leuconostoc* in *w*^*1118*^ *iso*. Data for Fig 5B–5F and S10A–S10G Fig. *w*^*1118*^ *iso*, *w*^*1118*^ DrosDel isogenic strain.
(CSV)

S10 Data Proliferation of *Lactobacillus* species in *w*^*1118*^ *iso*. Data for Fig 5G and S10H–S10J Fig. *w*^*1118*^ *iso*, *w*^*1118*^ DrosDel isogenic strain.
(CSV)

S11 Data *Acetobacter* growth on food. Data for S11 Fig.
(CSV)

S12 Data Proliferation of *Acetobacter thailandicus* in *w*^*1118*^ *iso* during 24 hours in bottles with chaser GF flies. Data for S12A and S12B Fig. GF, germ free (axenic); *w*^*1118*^ *iso*, *w*^*1118*^ DrosDel isogenic strain.
(CSV)

S13 Data Proliferation of *Acetobacter thailandicus* in *Drosophila melanogaster* and *D*. *simulans*. Data for Fig 5H and S12C–S12E Fig.
(CSV)

S14 Data Developmental time to pupariation and to adulthood of *w*^*1118*^ *iso* monoassociated with *Acetobacter thailandicus*, *Acetobacter* OTU2753, and axenic. Columns D–P correspond to number of new pupae or adults on Days 6–18 after egg laying. Data for S13 Fig. *w*^*1118*^ *iso*, *w1118* DrosDel isogenic strain.
(CSV)

S15 Data Colonization of *Acetobacter thailandicus* and *Acetobacter* OTU2753 in *w^1118^ iso* males and females at 0 days and 10 days after inoculation with the bacteria and being exposed to the stability protocol. nc—growth of bacteria in lowest dilution plate is too high to determine precisely CFUs. These data are represented as “above 10^5^ CFU/gut” in figures. Data for Fig 6B and S14A Fig. CFU, colony-forming unit; *w*^*1118*^ *iso*, *w*^*1118*^ DrosDel isogenic strain.
(CSV)

S16 Data Transmission of *Acetobacter thailandicus* to food. Columns D–H correspond to assessment of bacteria in the food on days 1, 3, 5, 7, and 9 of the experiment. No data—no data collected. nc—growth of bacteria in lowest dilution plate is too high to determine precisely CFUs. Data for Fig 6C and S14B Fig. CFU, colony-forming unit.
(CSV)

S17 Data Number of eggs laid by *w*^*1118*^ *iso* inoculated with *Acetobacter thailandicus*, *Acetobacter* OTU2753, or axenic, over the 10 days in cages. Columns D–M correspond to the number of eggs on Days 1–10 of the experiment. Data for Fig 6D and S14C Fig. *w*^*1118*^ *iso*, *w*^*1118*^ DrosDel isogenic strain.
(CSV)

S18 Data Developmental time of progeny from *w*^*1118*^ *iso* inoculated with *Acetobacter thailandicus*, *Acetobacter* OTU2753, or axenic over the 10 days in cages. Columns E–O correspond to the number of new emerged adults in bottles at Days 10–20 after egg laying. Progeny was counted in bottles collected on different days. Data for Fig 6E and 6F and S14D and S14E Fig. *w*^*1118*^ *iso*, *w*^*1118*^ DrosDel isogenic strain.
(CSV)

S19 Data Fertility from progeny from *w*^*1118*^ *iso* inoculated with *Acetobacter thailandicus*, *Acetobacter* OTU2753, or control. cagepair—cage from where the pairs were collected. daypair—pairs were collected from bottles of Day 9 or 10 of the experiment. vialday—progeny was counted in vials from different days. Pairs were placed in new food vials every other day until Day 8. Columns H–V correspond to the number of new emerged adults on Days 8–22 after egg laying. Data for Fig 6G and S14F Fig. *w*^*1118*^ *iso*, *w*^*1118*^ DrosDel isogenic strain.
(CSV)

S20 Data Developmental time to pupariation, adulthood, and respective total number of progeny from one or both parents monoassociated with *Acetobacter thailandicus*. Conditions used: 53F + 53M—both parents associated with *A*. *thailandicus*. GFF + GFM—both parents axenic. 53F + GFM—female with *A*. *thailandicus*, male axenic. GFF + 53M—female axenic, male with *A*. *thailandicus*. GFF + GFM + Bact—both parents axenic and *A*. *thailandicus* added. Vialday—progeny were counted in vials from different days; pairs were placed in new food vials every other day until Day 8. Columns F–W correspond to number of new pupae or emerged adults on Days 5–22 after egg laying. Data for Fig 6H and S15 Fig. Bact, *A*. *thailandicus*; GFF, germ free (axenic) females; GFM, germ free (axenic) males.
(CSV)

S21 Data Developmental time to adulthood from *w*^*1118*^ *iso* associated with different bacterial isolates. Columns E–O correspond to number of new emerged adults on Days 9–19 after egg laying. Data for Fig 7B, S16A–S16D Fig and S17A Fig. *w*^*1118*^ *iso*, *w*^*1118*^ DrosDel isogenic strain.
(CSV)

S22 Data Fertility of *w*^*1118*^ *iso* developed with different bacterial isolates. Data for Fig 7B, S16E and S16F Fig and S17B Fig. *w*^*1118*^ *iso*, *w*^*1118*^ DrosDel isogenic strain.
(CSV)

S23 Data Developmental time of axenic *w*^*1118*^ *iso*, associated with *Acetobacter thailandicus* or associated with *Acetobacter* OTU2753 in sterile fig homogenate. Columns D–AD correspond to the number of new emerged adults on days 9–35 after egg laying. Data for Fig 7D and 7E and S18A–S18F Fig. *w*^*1118*^ *iso*, *w*^*1118*^ DrosDel isogenic strain.
(CSV)

S24 Data Fertility of axenic *w*^*1118*^ *iso* associated with *Acetobacter thailandicus* or associated with *Acetobacter* OTU2753 in sterile fig homogenate. Data for Fig 7G and S18G–S18I Fig. *w*^*1118*^ *iso*, *w*^*1118*^ DrosDel isogenic strain.
(CSV)

S25 Data Developmental time of *w*^*1118*^ *iso* with and without the addition of *Acetobacter thailandicus* in freshly collected figs. Columns E–U correspond to the number of new emerged adults on Days 9–25 after egg laying. Data for Fig 7I and 7J and S18J and S18K Fig. *w*^*1118*^ *iso*, *w*^*1118*^ DrosDel isogenic strain.
(CSV)

## Abbreviations

AIC: Akaike information criterion
BHI: Brain heart infusion
CFU: colony-forming unit
FISH: fluorescent in situ hybridization
glm-binomial: generalized linear model with binomial distribution
LB: Lysogeny Broth
lm: linear model
lmm: linear mixed model
MRS: de Man, Rogosa and Sharpe broth
OTU: operational taxonomic unit
PB: phosphate buffer
PBS: phosphate-buffered saline
PBST: 1% Triton-X-100 in PBS 1×
PI: propidium iodide
qPCR: quantitative PCR
RT: room temperature
TEM: transmission electron microscopy
w^1118^ iso: *w*^*1118*^ DrosDel isogenic strain
V2: hypervariable region 2 of 16S rRNA gene
V4: hypervariable region 4 of 16S rRNA gene
