# Supplementary figures and images for: Drosophila melanogaster establishes a species-specific mutualistic interaction with stable gut-colonizing bacteria
Source: PLoS Biol. 2018 Jul 5;16(7):e2005710. doi: 10.1371/journal.pbio.2005710 (PMC6049943; doi:10.1371/journal.pbio.2005710)

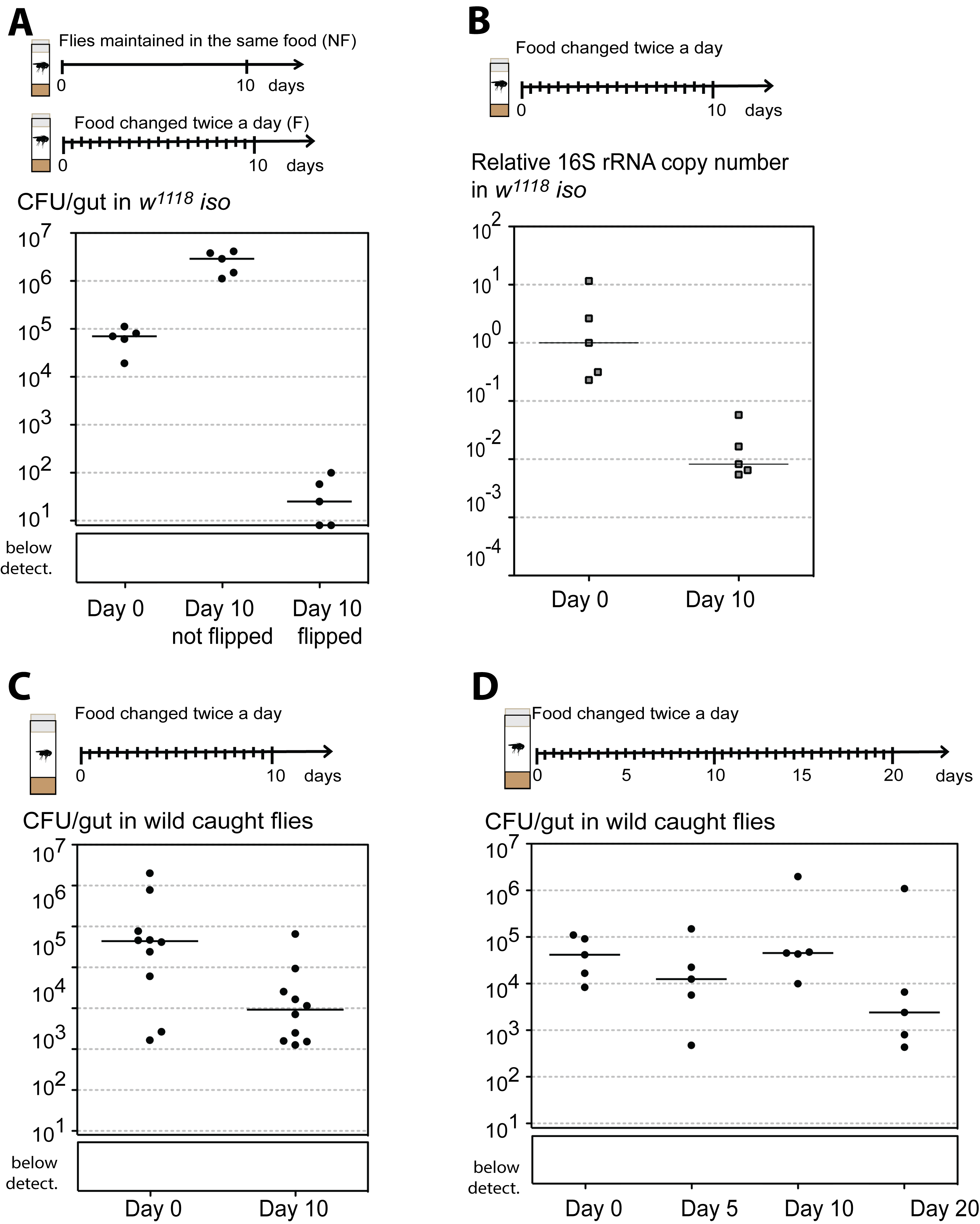

Supplement: S1 Fig — Single 3–6-day-old w1118 iso males were kept in the same vial during 10 days (A) or exposed to a stability protocol by being passed to new vials twice a day (A, B). (A) Five individuals were analyzed at each day, and total number of CFUs per gut was determined by bacterial plating. Bacterial levels increase in the flies maintained in the same vials and decrease in the flies flipped to new vials twice a day (lmm, p < 0.001 for both). Supporting data can be found in S1 and S2 Data. (B) Relative amount of 16S rRNA bacterial gene was measured by quantitative-PCR in five individual guts from each day, using the host gene Rpl32 as a reference gene. Relative amount of 16S rRNA gene decreases between days (lmm, p < 0.001). Supporting data can be found in S3 Data. (C, D) Bacterial levels from wild-caught flies on the day of collection (Day 0) and after 5, 10, or 20 days of the stability protocol. Bacterial levels in the flies significantly decrease with time (lmm, p = 0.004). Supporting data can be found in S5 Data. Each dot represents an individual gut and the lines represent medians. Statistical analyses were performed together with replicate experiments shown in Fig 1. CFU, colony-forming unit; lmm, linear mixed model; w1118 iso, w1118 DrosDel isogenic strain. (TIF) [file pbio.2005710.s001.tif]

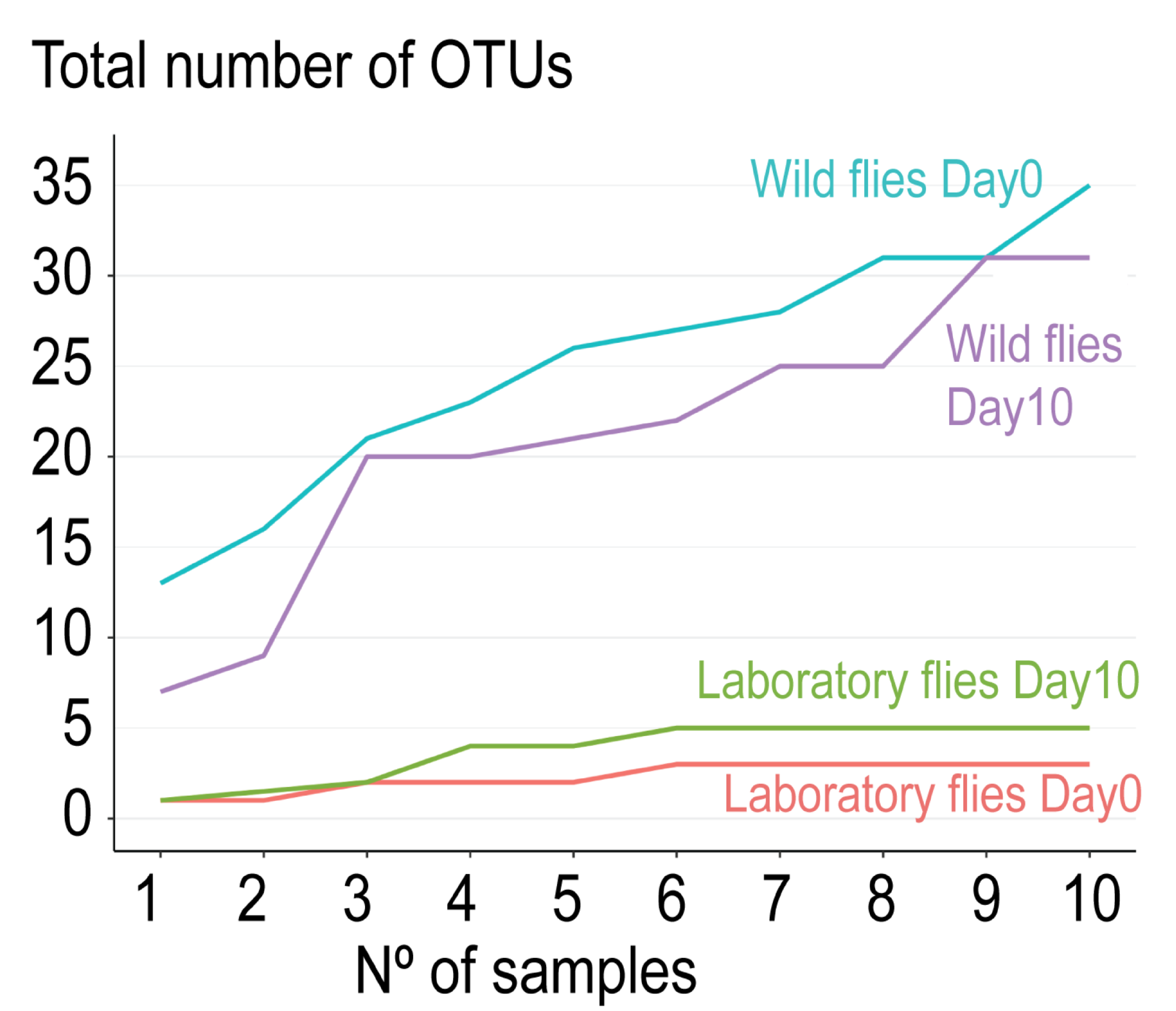

Supplement: S2 Fig — Accumulation curve of the different bacterial OTUs present in wild-caught and laboratory flies before (Day 0) and after (Day 10) being exposed to the stability protocol. Supporting data can be found in S6 Data. OTU, operational taxonomic unit. (TIF) [file pbio.2005710.s002.tif]

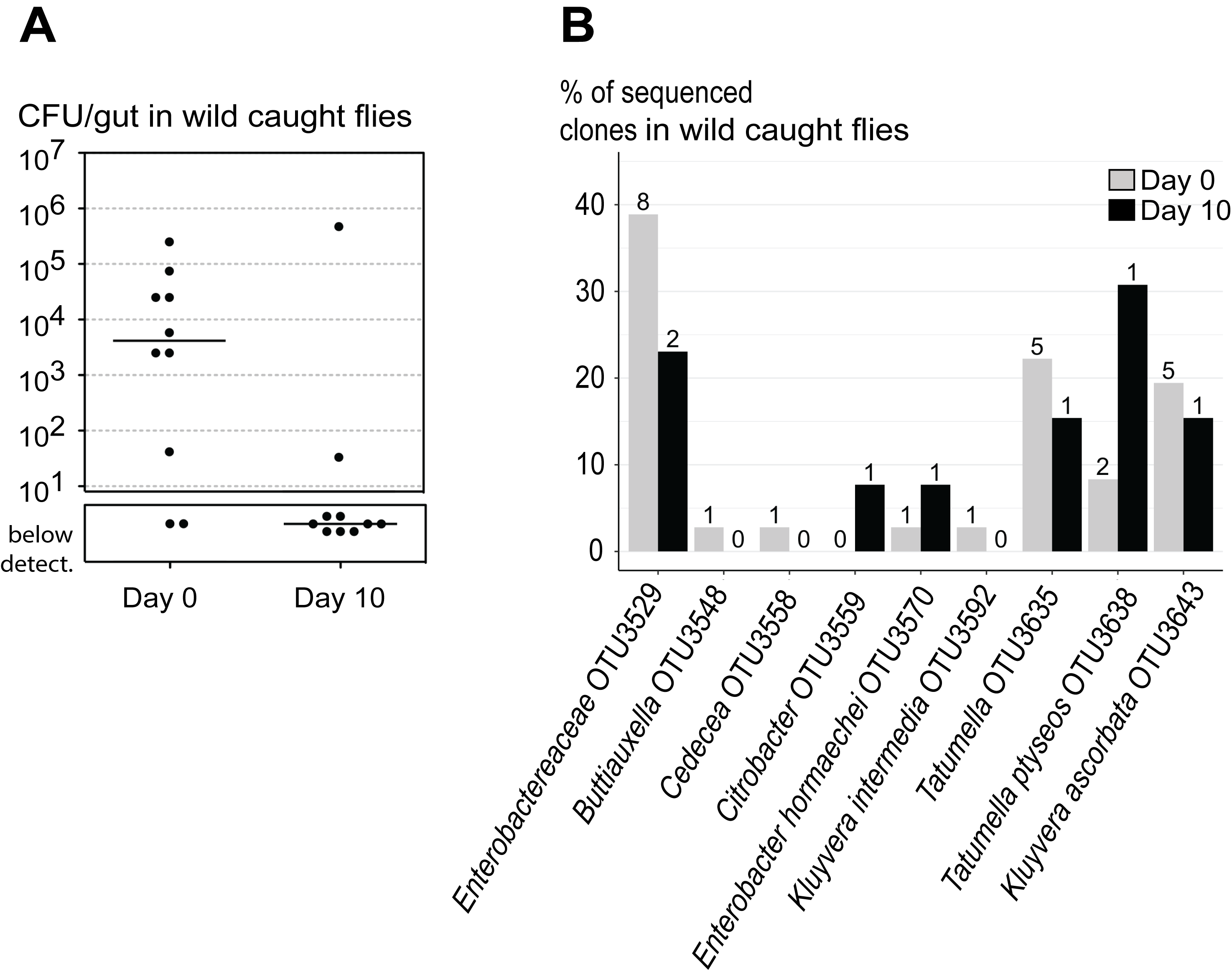

Supplement: S3 Fig — (A) Levels of Enterobacteriaceae in the gut of wild-caught flies before (Day 0) and after 10 days of the stability protocol (Day 10). Each dot represents one gut and lines represent medians. Levels of Enterobacteriaceae decrease between days (lm, p = 0.01). (B) Frequencies of sequenced colonies of Enterobacteriaceae for Day 0 and Day 10, represented as several in Fig 2. Numbers on the top of the bars correspond to the number of flies carrying that specific OTU, from a total of 10 flies. Supporting data can be found in S6 Data. lm, linear model; OTU, operational taxonomic unit. (TIF) [file pbio.2005710.s003.tif]

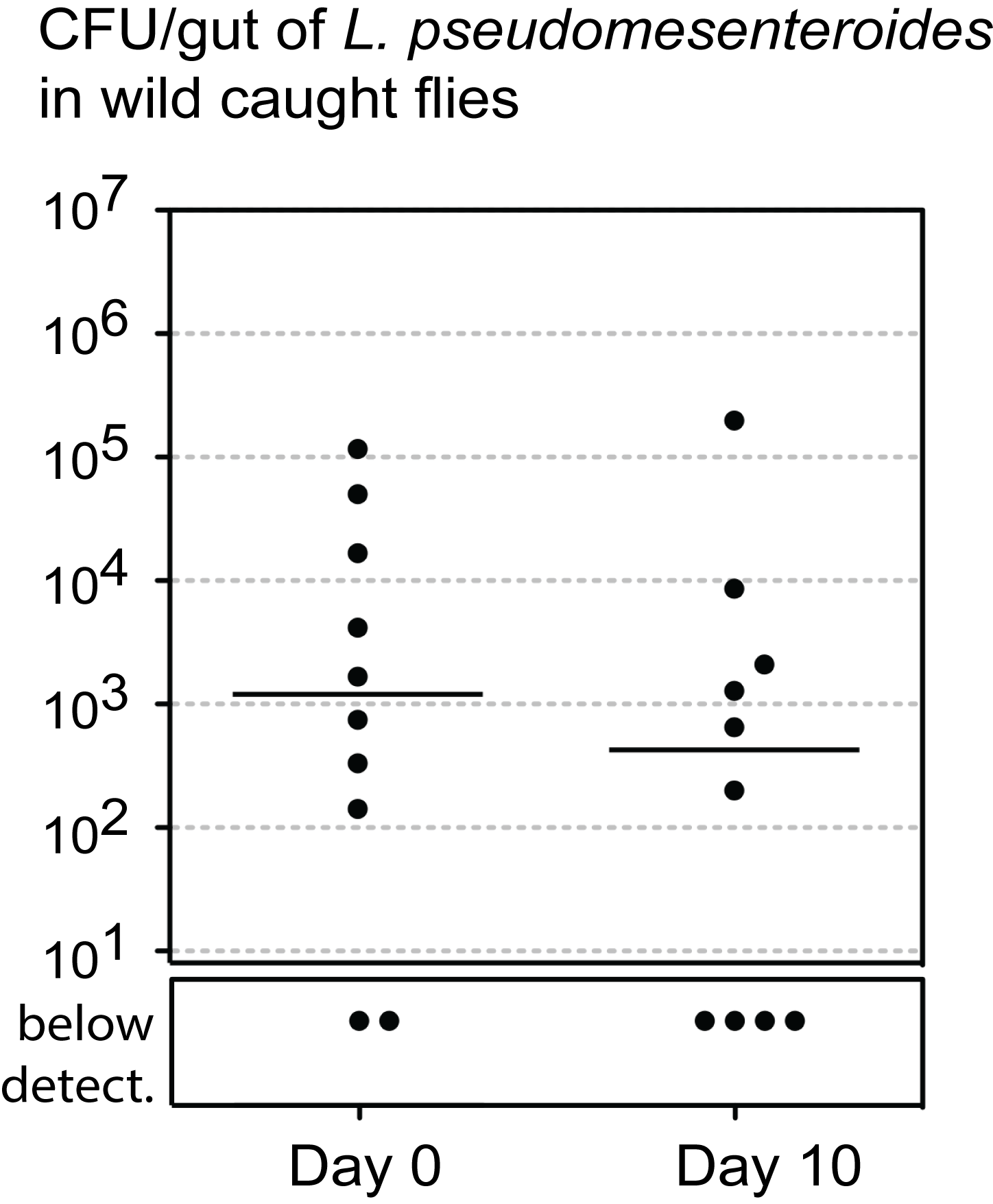

Supplement: S4 Fig — Total L. pseudomesenteroides levels in the gut of wild-caught flies on the day of collection (Day 0) and after 10 days of the stability protocol (Day 10). Levels of L. pseudomesenteroides are not significantly different between days (lm, p = 0.372). Each dot represents one gut and the line represents the median. Supporting data can be found in S6 Data. lm, linear model. (TIF) [file pbio.2005710.s004.tif]

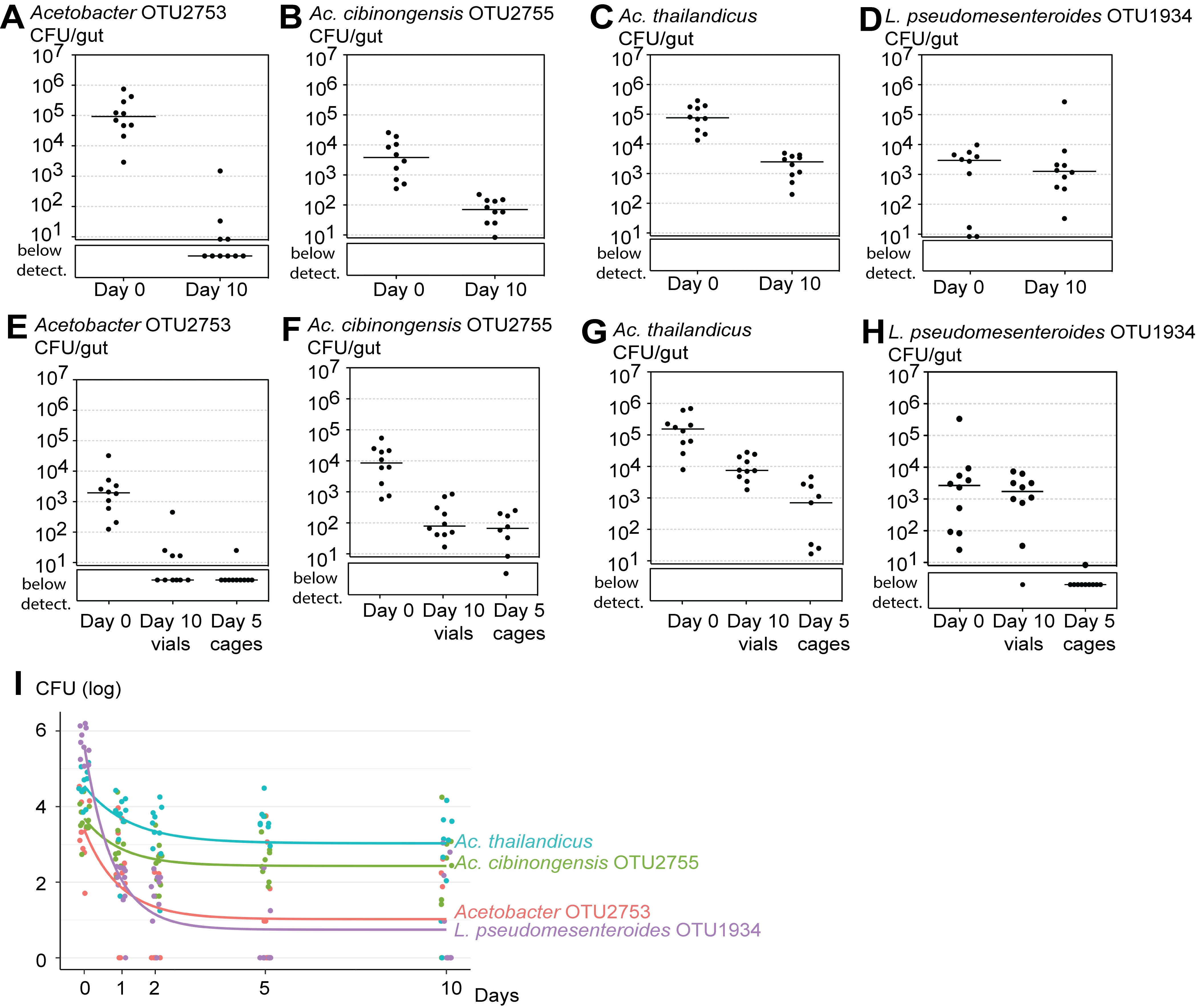

Supplement: S5 Fig — Single 3–6-day-old w1118 iso males from monoassociated stocks with Acetobacter OTU2753 (A, E), A. cibinongensis OTU2755 (B, F), A. thailandicus (C, G), or Leuconostoc pseudomesenteroides (D, H) were exposed to the stability protocol for 10 days in vials (A–H) or 5 days in cages (E–H). Number of CFUs in individual guts was assessed by plating before and after 5 or 10 days of the stability protocol. Ten flies were analyzed for each condition. Acetobacter OTU2753, A. cibinongensis, and A. thailandicus levels decrease between Day 0 and Day 10 in vials (lmm, p < 0.001 for all), but L. pseudomesenteroides levels do not significantly change (p = 0.96). (I) Data from Fig 4B–4E were fitted to an exponential decay model that estimates the exponential decay rate, which corresponds to the rate of bacterial loss from the gut, and an asymptote, which corresponds to the levels at which the bacteria levels tend to stabilize after this loss. The rate of decay is the same for all the bacteria, but there are differences between the asymptotes of all bacteria (contrasts of nonlinear least-squares model estimates, p < 0.014), except between Acetobacter OTU2753 and L. pseudomesenteroides (p = 0.116). Supporting data can be found in S7 Data. CFU, colony-forming unit; lmm, linear mixed model fit; w1118 iso, w1118 DrosDel isogenic strain. (TIF) [file pbio.2005710.s005.tif]

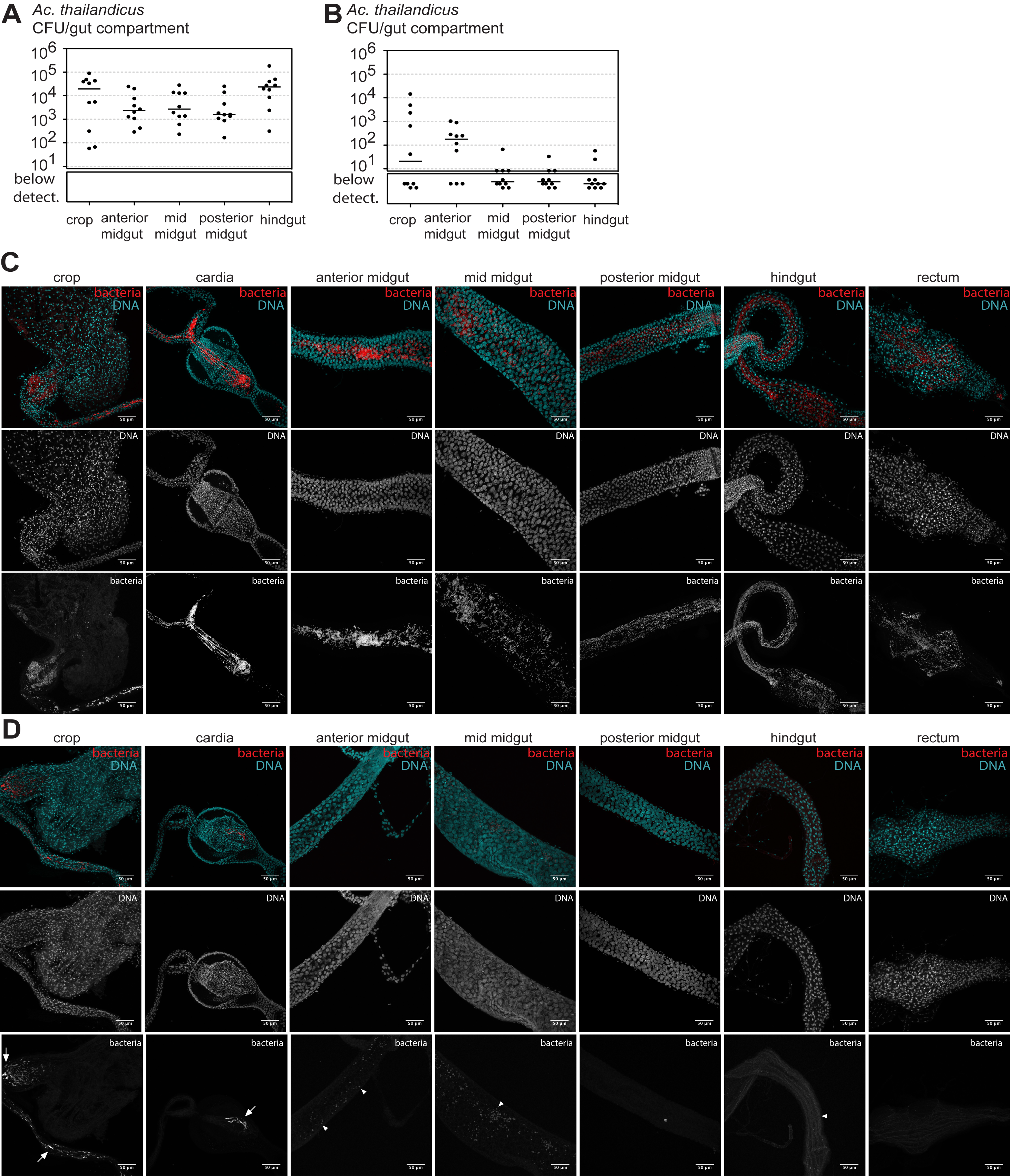

Supplement: S6 Fig — (A, B) Number of CFUs in each gut compartment from w1118 iso males monoassociated with A. thailandicus before (A) and after (B) 5 days of the stability protocol. Each dot represents one gut or one gut fragment and lines represent medians. Supporting data can be found in S8 Data. (C, D) Fluorescent in situ hybridization with Cy3 labeled Bacteria 16S rRNA universal probe EUB338 for bacteria in the gut of males monoassociated with A. thailandicus at Day 0 (C) and Day 5 (D) of the stability protocol. On Day 0, bacteria are found in all gut compartments, while at Day 5, bacteria persist in the crop, crop duct, and proventriculos. On (D), arrows point to bacteria and arrowheads to autofluorescence. Scale bar corresponds to 50 μm. CFU, colony-forming unit; Cy3, cyanine dye 3; w1118 iso, w1118 DrosDel isogenic strain. (TIF) [file pbio.2005710.s006.tif]

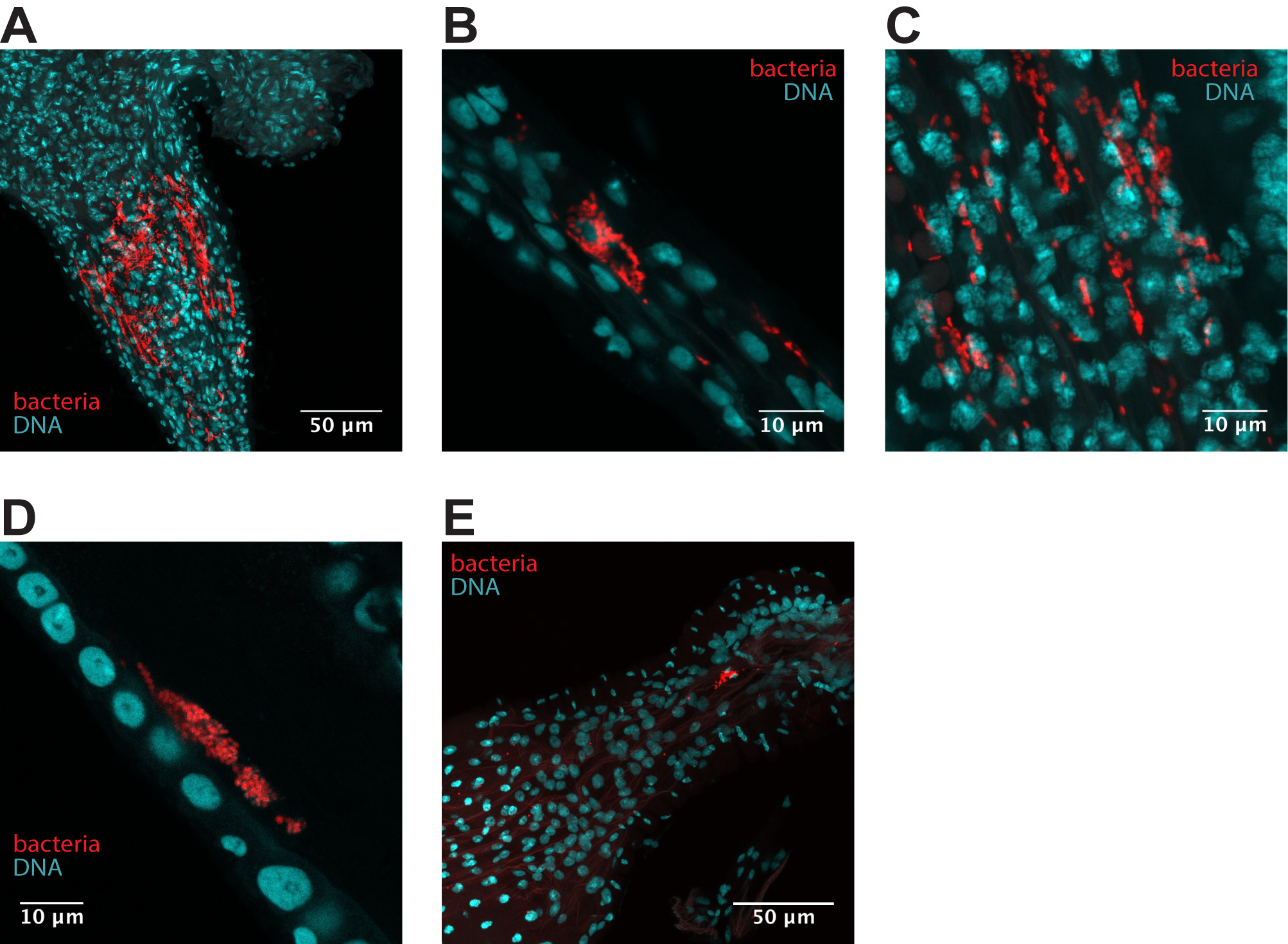

Supplement: S7 Fig — Fluorescent in situ hybridization with Cy3 labeled Bacteria 16S rRNA universal probe EUB338 for bacteria was performed in flies monoassociated with A. thailandicus 5 days after the stability protocol. Similar to males, bacteria persist in the anterior part of the crop (A), crop duct (B), and proventriculus (C) of females. (D, E) In a few cases, bacteria were also found in the anterior midgut (D) or in the rectum of flies (E). DNA was stained with Hoechst. Scale bar corresponds to 50 μm in (A, E) and to 10 μm in (B–D). Cy3, cyanine 3 dye. (TIF) [file pbio.2005710.s007.tif]

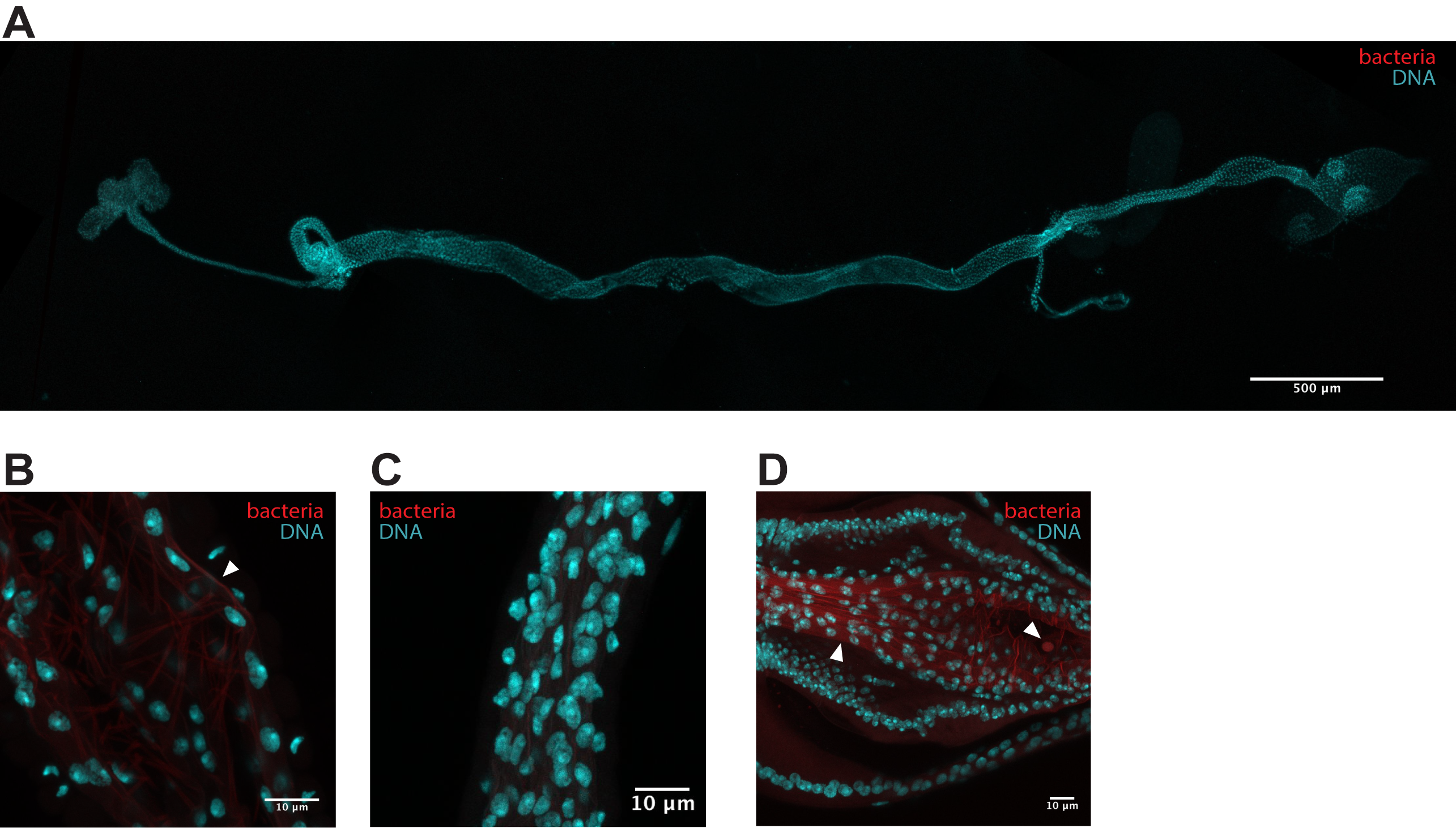

Supplement: S8 Fig — Fluorescent in situ hybridization with Cy3 labeled Bacteria 16S rRNA universal probe EUB338for bacteria was performed in axenic flies as a control. Full gut (A), anterior part of the crop (B), crop duct (C), and proventriculus (D). Arrowheads point to autofluorescence of chitin and food. DNA was stained with Hoechst. Scale bar corresponds to 500 μm in (A) and to 10 μm in B–D. Cy3, cyanine 3 dye. (TIF) [file pbio.2005710.s008.tif]

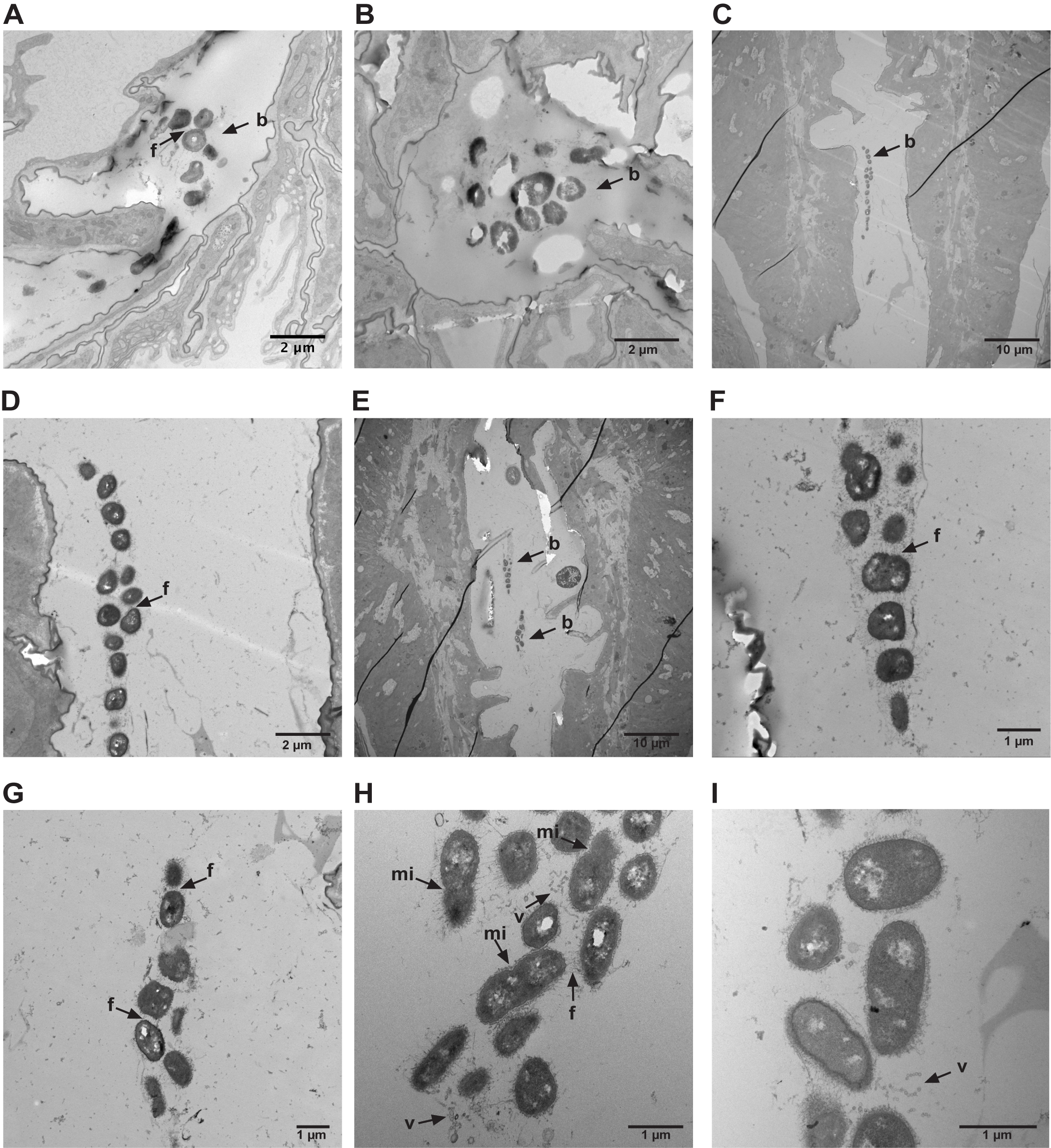

Supplement: S9 Fig — Clusters of bacterial cells (b) near the chitin folds of the crop (A, B) and in the lumen of the proventriculus (C–I) observed by scanning electron microscopy. Some cells present membrane invaginations (mi) and seem to be dividing. Cells seem to be attached to each other by external appendages, such as fimbriae (f). Extracellular vesicles are found between cells (v). Scale bar corresponds to 10 μm in (C, E), 2 μm in (A, B, D), and 1 μm in (F, G, H, I). (D) is a magnification of (C). (F) and (G) are magnifications of (E). b, bacterial cell; f, fimbriae; mi, membrane invagination; v, vesicles. (TIF) [file pbio.2005710.s009.tif]

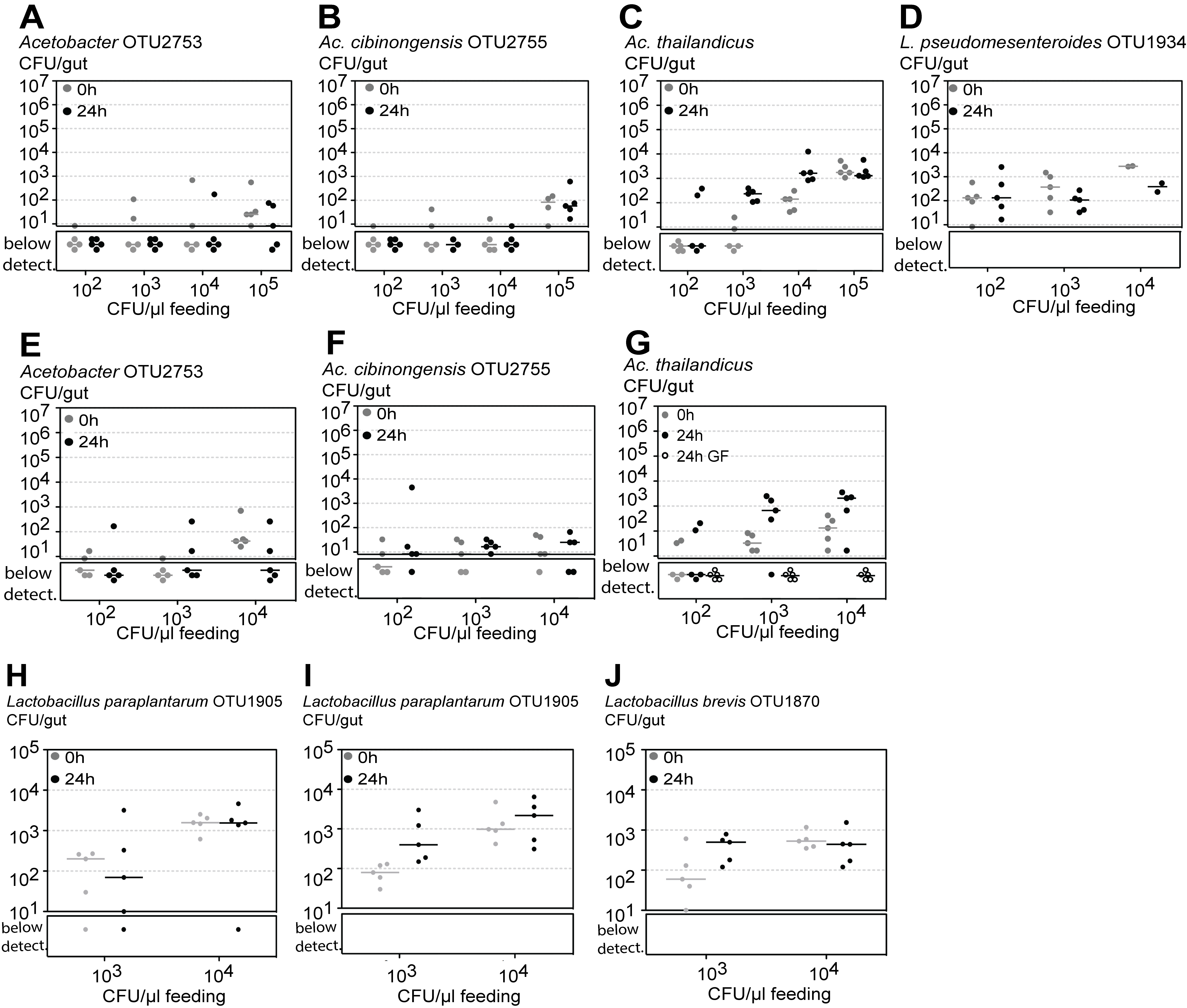

Supplement: S10 Fig — Three- to six-day-old axenic w1118 iso males were inoculated for 6 hours with different concentrations of Acetobacter OTU2753 (A, E), A. cibinongensis OTU2755 (B, F), A. thailandicus (C, G), Leuconostoc pseudomesenteroides (D), Lactobacillus paraplantarum (H, I), and L. brevis (J). Bacterial levels were assessed by plating 0 and 24 hours postinoculation. During this period, males were singly placed in cages. In (G), axenic chaser males were placed in cages together with males inoculated with A. thailandicus. At 24 hours, bacterial levels were assessed in both males. Bacterial levels between 0 and 24 hours decrease in flies inoculated with Acetobacter OTU2753 (lmm, p < 0.001), increase in flies inoculated with A. cibinongensis, A. thailandicus, and L. brevis (lmm, p = 0.024, p < 0.001, and p = 0.046, respectively) and do not significantly change in flies inoculated with L. pseudomesenteroides and L. paraplantarum (lmm, p = 0.158 and p = 0.65, respectively). Four to five males were used per condition, except in (B), in which three males were used at one time point and in (D), in which two males were used on the inoculation 104 CFU/μL. Each dot represents one gut and lines represent medians. Statistical analyses were performed together with replicate experiments shown in Fig 5B–5G. Supporting data can be found in S9 and S10 Data. CFU, colony-forming unit; lmm, linear mixed model; w1118 iso, w1118 DrosDel isogenic strain. (TIF) [file pbio.2005710.s010.tif]

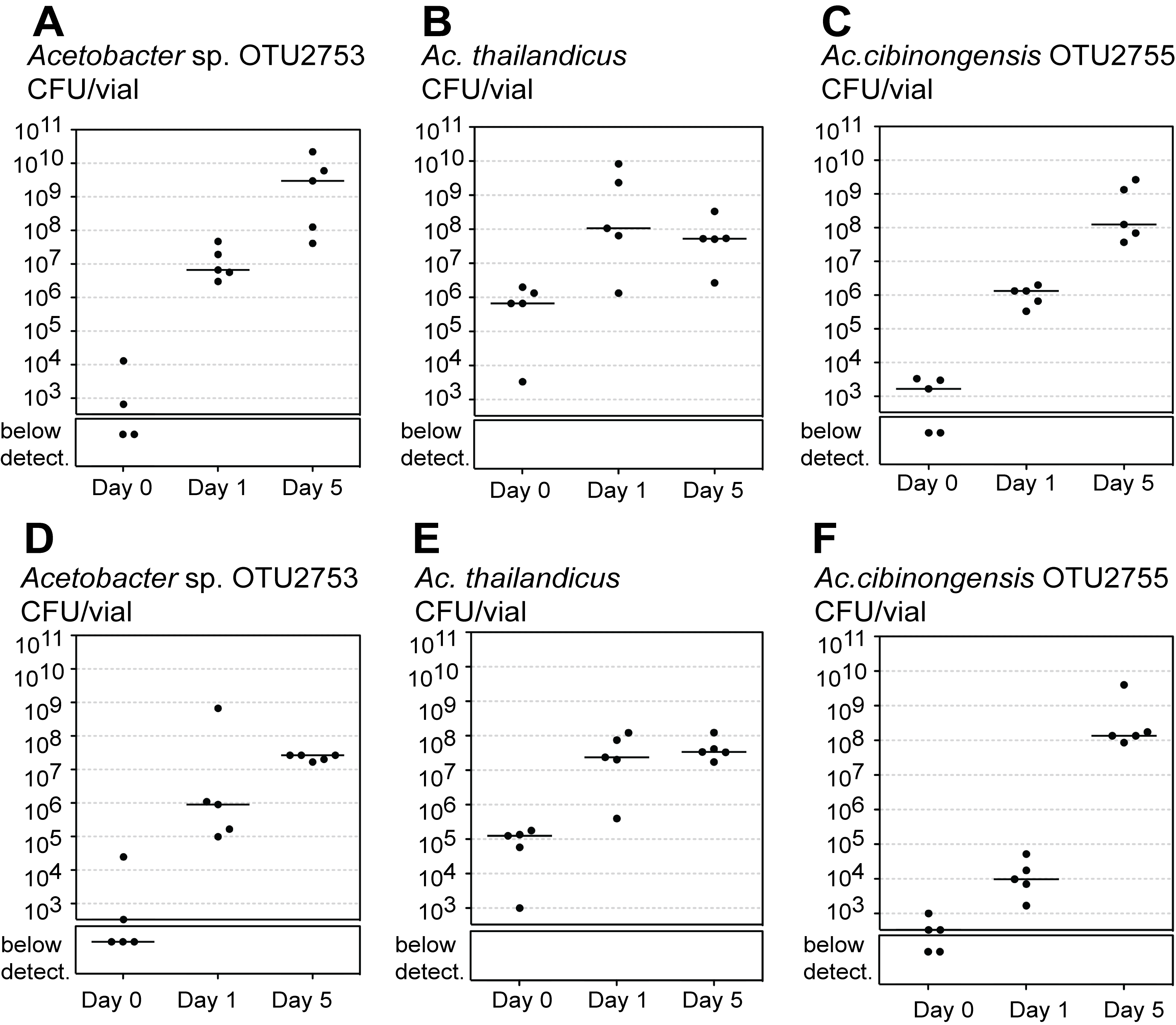

Supplement: S11 Fig — Single 3–6-day-old w1118 iso males from a monoassociated stock with Acetobacter OTU2753 (A, D), A. thailandicus (B, E), or A. cibinongensis (C, F) were placed per vial for a period of 16 hours and then discarded. Bacterial levels on the food were determined by plating after discarding the flies (Day 0) and after 1 or 5 days of incubating these vials. Levels of Acetobacter on the food increase for all conditions between Day 0 and Day 5 (lmm, p < 0.001). Five vials were used per condition. Each dot represents the bacterial levels on the food of one vial and lines represent medians. Supporting data can be found in S11 Data. lmm, linear mixed model; w1118 iso, w1118 DrosDel isogenic strain. (TIF) [file pbio.2005710.s011.tif]

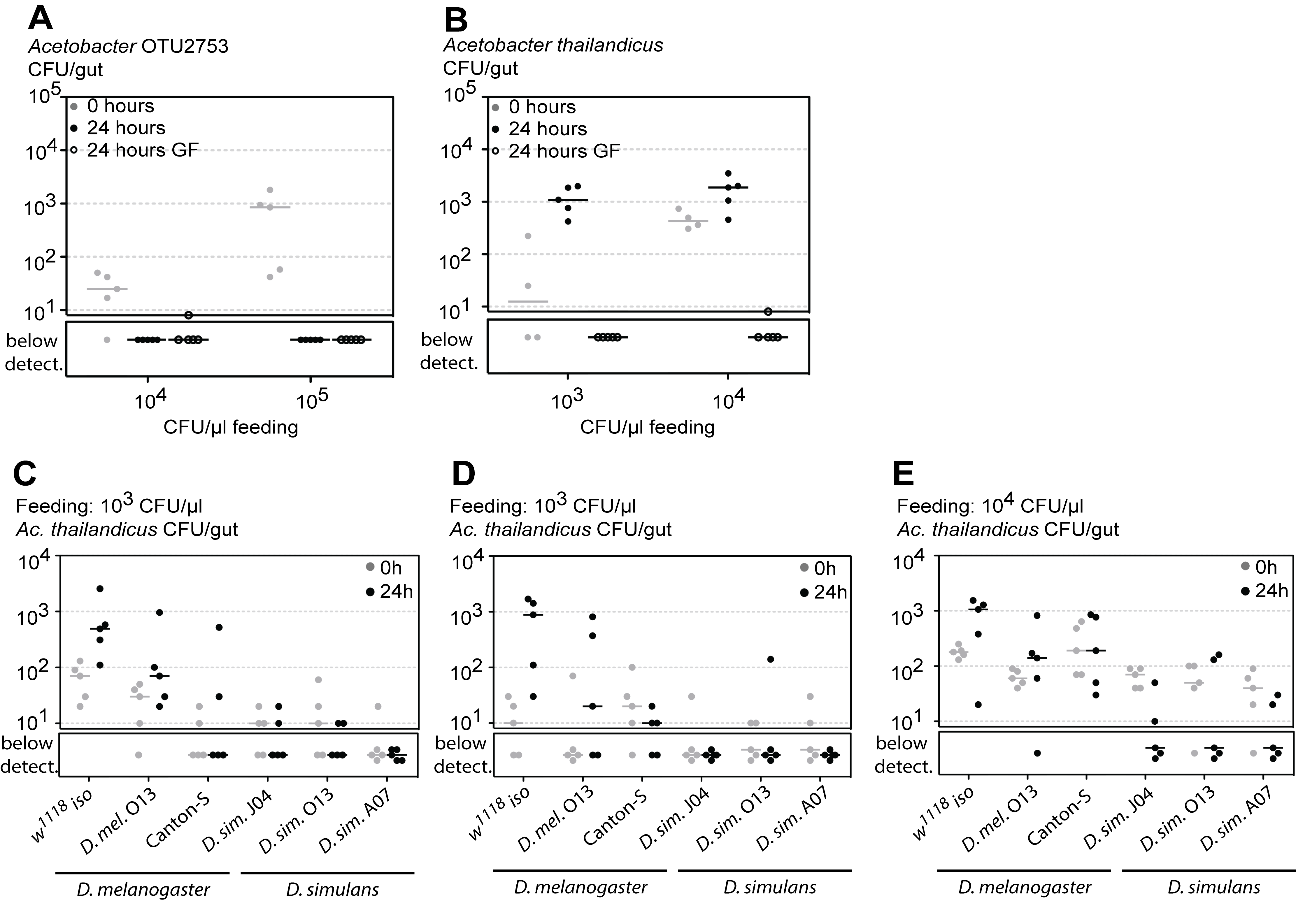

Supplement: S12 Fig — (A, B) Optimization of proliferation protocol in bottles. Axenic 3–6-day-old w1118 iso were inoculated for 6 hours with different concentrations of Acetobacter OTU2753 (A) or A. thailandicus (B). Bacterial levels were assessed 0 and 24 hours postinoculation. During this period, males were singly placed in bottles (food surface of 28 cm2) together with an axenic chaser male, from which bacterial levels were also assessed at 24 hours. Levels of Acetobacter OTU2753 decrease between days (lmm, p < 0.001). Levels of A. thailandicus increase when flies are inoculated with the lowest concentration (p < 0.001) and are maintained when flies are inoculated with the highest concentration (p = 0.426). Supporting data can be found in S12 Data. (C–E) Axenic 3–6-day-old D. melanogaster or D. simulans males were inoculated for 6 hours with 103 CFU/μL (C, D) or 104 CFU/μL (E) of A. thailandicus. Bacterial levels were assessed 0 and 24 hours postinoculation. During this period, males were singly placed in bottles. Three different genetic backgrounds of D. melanogaster (w1118 iso, D. mel. O13, and Canton-S) and of D. simulans (D. sim. J04, D. sim. O13, and D. sim. A07) were used. Bacterial levels in the gut increase in D. melanogaster and decrease in D. simulans (p < 0.001). Supporting data can be found in S13 Data. Five individuals were analyzed for each condition, per experimental replicate, and total number of CFUs per gut determined by plating. Each dot represents one gut and the line represents medians. Statistical analyses were performed together with the replicate experiment shown in Fig 5H. CFU, colony-forming unit; lmm, linear mixed model; w1118 iso, w1118 DrosDel isogenic strain. (TIF) [file pbio.2005710.s012.tif]

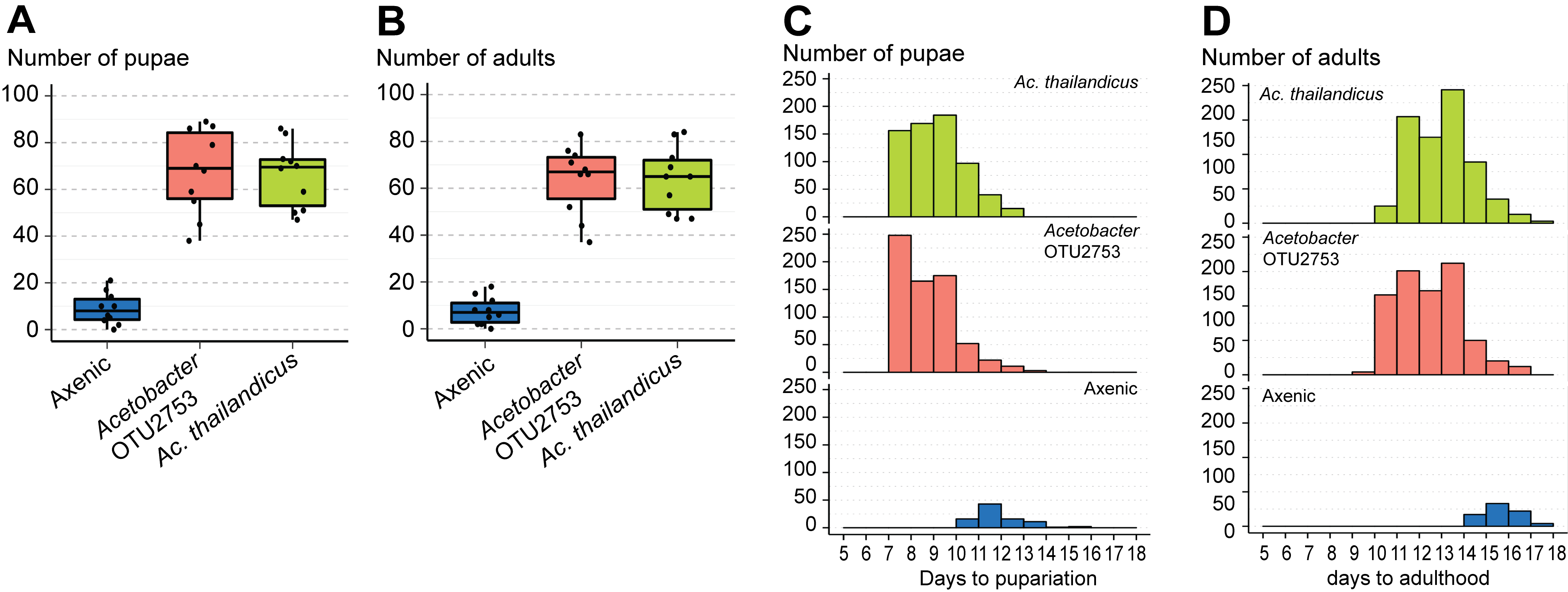

Supplement: S13 Fig — Total number of pupae (A), total number of adults (B), developmental time to pupariation (C), and developmental time to adulthood (D) were analyzed in flies from a monoassociated stock with Acetobacter OTU2753 or A. thailandicus, or in axenic flies. One female and three males from each condition were placed per vial for 3 days and then discarded. Number of pupae or emerged adults was daily assessed. Ten vials were used per condition. Flies monoassociated with either Acetobacter species develop faster and have higher fertility than axenic flies (lm, p < 0.001). (A, B) Each dot represents the total progeny of one female. Supporting data can be found in S14 Data. lm, linear model. (TIF) [file pbio.2005710.s013.tif]

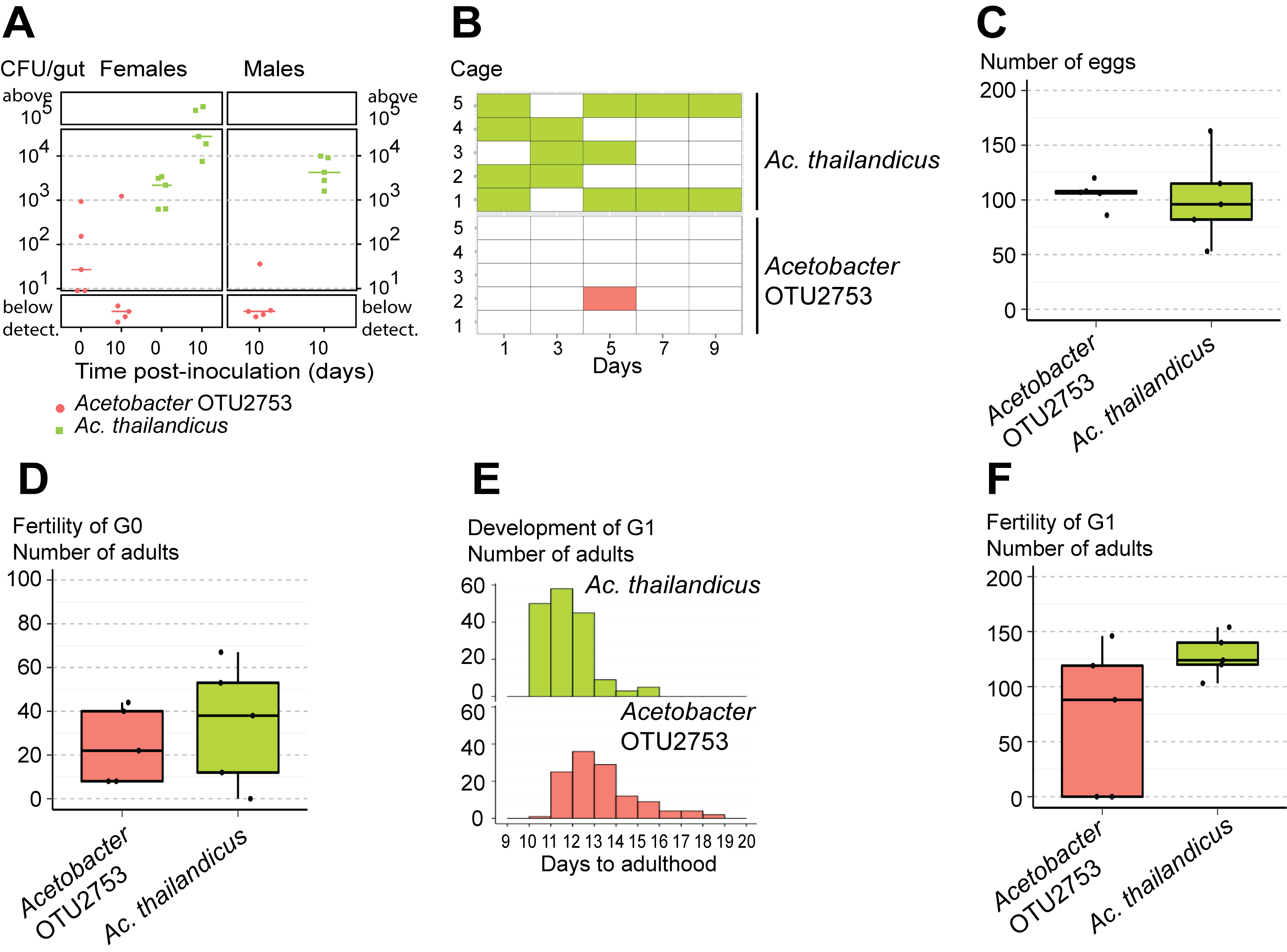

Supplement: S14 Fig — Axenic 1–3-day-old w1118 iso males and females (G0) were in contact with an inoculum of 105 CFU/μL of Acetobacter OTU2753 or A. thailandicus for 6 hours. Two males and one female were placed per cage, with five cages for each condition, during 10 days, with daily changed food. (A) Bacterial levels in single guts of females 0 days and 10 days postinoculation and in males 10 days postinoculation, analyzed by plating. Bacterial levels between the two time points increased in females inoculated with A. thailandicus and decreased in females inoculated with Acetobacter OTU2753 (Mann–Whitney test, p < 0.001 and p = 0.048, respectively). Supporting data can be found in S15 Data. (B) Presence of bacteria on the food collected from cages at days 1, 3, 5, 7, and 9 of the protocol, analyzed by plating. Filled rectangles represent presence of bacteria. A. thailandicus is transmitted to the food with higher frequency than Acetobacter OTU2753 (glm-binomial, p < 0.001). Supporting data can be found in S16 Data. (C–F) Effect of bacterial association on the fitness of D. melanogaster. Total number of eggs laid by flies inoculated with different Acetobacter (C) and total number of adults that emerged from these eggs (D). Total number of eggs or adults is not different between conditions (lmm, p > 0.484). (E) Developmental time to adulthood of the progeny (G1) of flies inoculated with different Acetobacter. Developmental time to adulthood is faster in progeny from flies inoculated with A. thailandicus than in progeny from flies inoculated with Acetobacter OTU2753 (lmm, p < 0.001). Supporting data can be found in S17 and S18 Data. (F) Fertility of G1. Two males and one female of G1 were placed per vial and flipped every other day for 10 days. Five couples were made per condition. Total number of emerged adults was analyzed. Fertility is higher in progeny from flies inoculated with A. thailandicus than in progeny from flies inoculated with Acetobacter OTU2753 (lmm, p < 0.001). Supporting [file pbio.2005710.s014.tif]

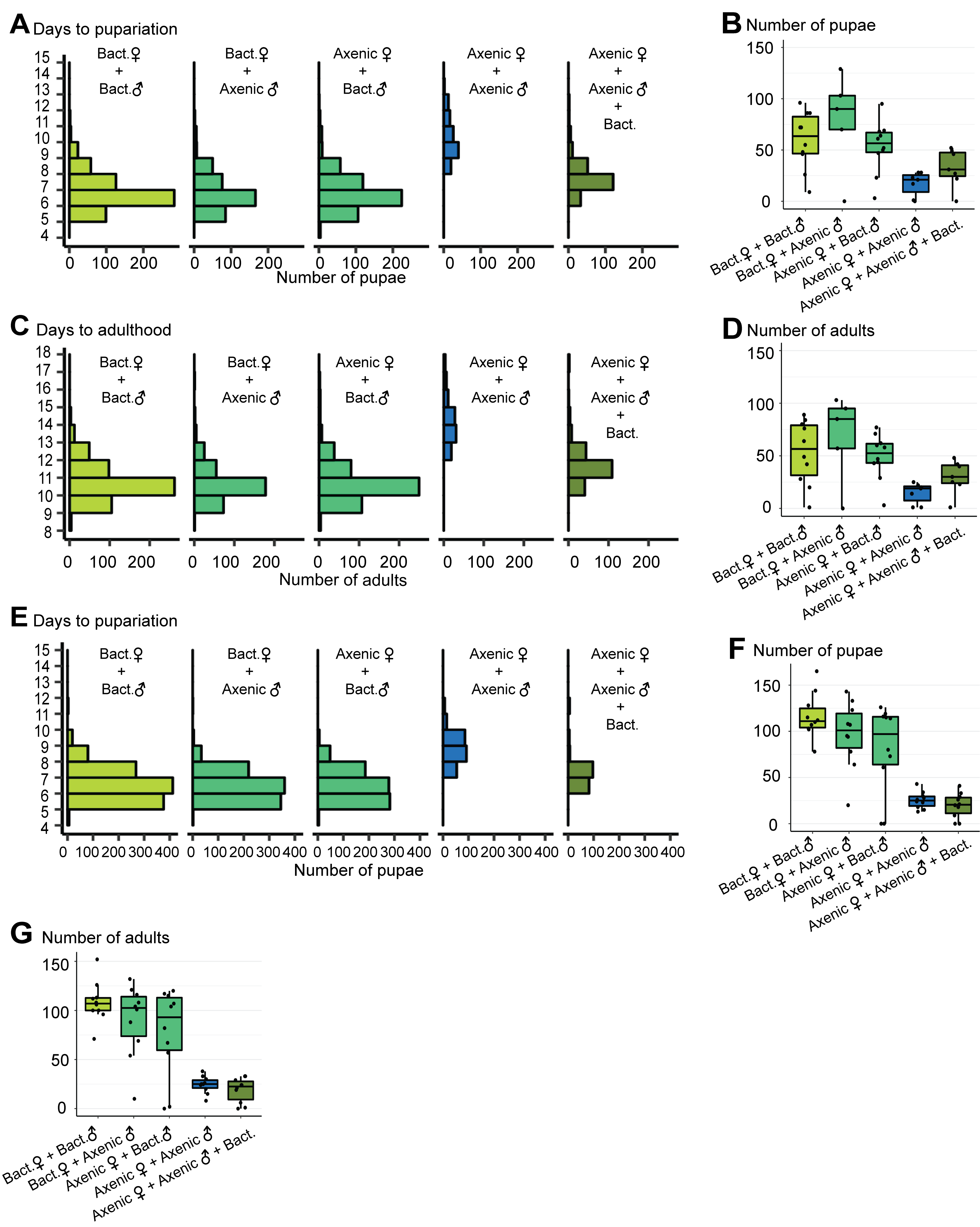

Supplement: S15 Fig — Combinations of one male and one female 1–2 days old w1118 iso, either axenic or monoassociated with A. thailandicus (Bact.), were placed in vials and flipped every other day for 10 days. To one set of vials with axenic parents, A. thailandicus was added on the eggs after passing the parents. Ten couples were made per condition. Developmental time to pupariation (A, E), to adulthood (C), total number of pupae (B, F), and total number of adults (D, G) was assessed. (A–D) correspond to one experimental replicate and (E–G) correspond to another experimental replicate, together with data from Fig 6H. Progeny from couples in which either or both parents are monoassociated and progeny from axenic flies in which A. thailandicus culture is added on the eggs develop faster than progeny from axenic flies (lmm, p < 0.001, for all these comparisons). Total number of progeny (pupae or adults) from couples in which either or both parents are monoassociated with A. thailandicus is higher than in progeny from axenic flies (lmm, p < 0.001). (B, D, F, G) Each dot represents the total progeny of one female. Statistical analyses were performed together with replicate experiment shown in Fig 6H. Supporting data can be found in S20 Data. Bact., A. thailandicus; lmm, linear mixed model; w1118 iso, w1118 DrosDel isogenic strain. (TIF) [file pbio.2005710.s015.tif]

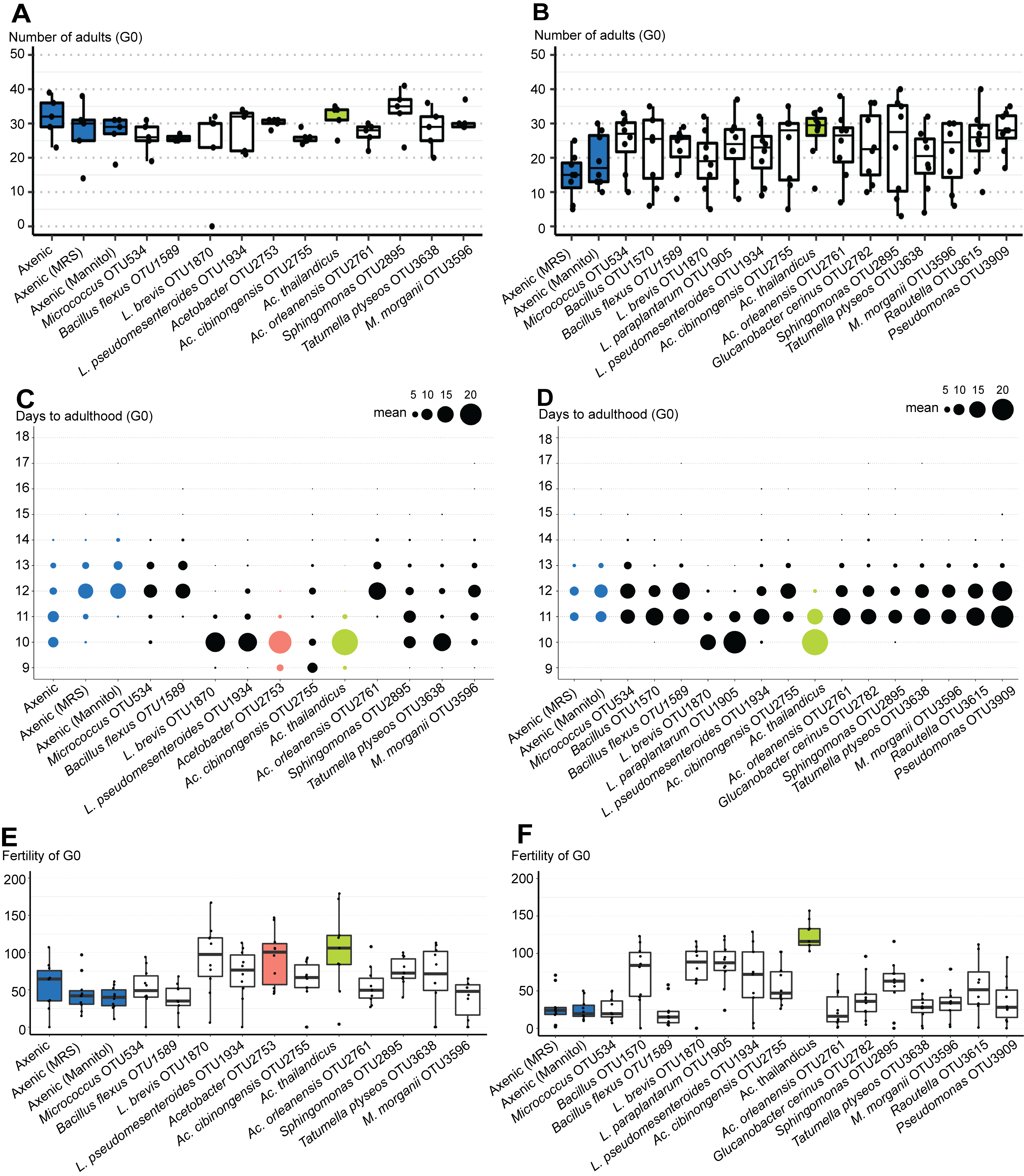

Supplement: S16 Fig — Fifty w1118 iso eggs were associated with different bacteria isolated from the gut of wild-caught Drosophila melanogaster. As controls, axenic eggs that had no treatment (GF) or in which sterile media were added (GF MRS and GF Mannitol) were used. Ten vials were used for each condition. Total number of emerged adults (A, B) and their developmental time to adulthood was daily assessed (C, D). Number of emerged adults is not significantly different between conditions (lmm, p > 0.282 for all pairwise comparisons). Flies from eggs associated with Acetobacter thailandicus developed faster than from axenic eggs or eggs associated with 11 out of the other 15 bacteria (lmm, p < 0.038 for these pairwise comparisons). Supporting data can be found in S21 Data. (E, F) Fertility of G0 was assessed. Two males and one female that developed in the presence of different bacteria (G0) were placed per vial and flipped every other day for 8 (E) or 10 (F) days. Five couples were made per condition. Total number of emerged adults was analyzed. Flies associated with A. thailandicus are more fertile than axenic flies or flies associated with 11 out of the other 15 bacteria (lmm, p < 0.018). Supporting data can be found in S22 Data. (A, C, E) and (B, D, F) correspond to two experimental replicates. Correlation between developmental time and fertility is represented in Fig 7B. Each dot represents the total progeny of one female (A, B, E, F) and the size of the circle represents the mean number of adults that emerged per day (C, D). Statistical groups of significance for C, D, E, F are shown in S17 Fig. lmm, linear mixed model; GF, germ free (axenic); MRS, de Man, Rogosa and Shrape broth. (TIF) [file pbio.2005710.s016.tif]

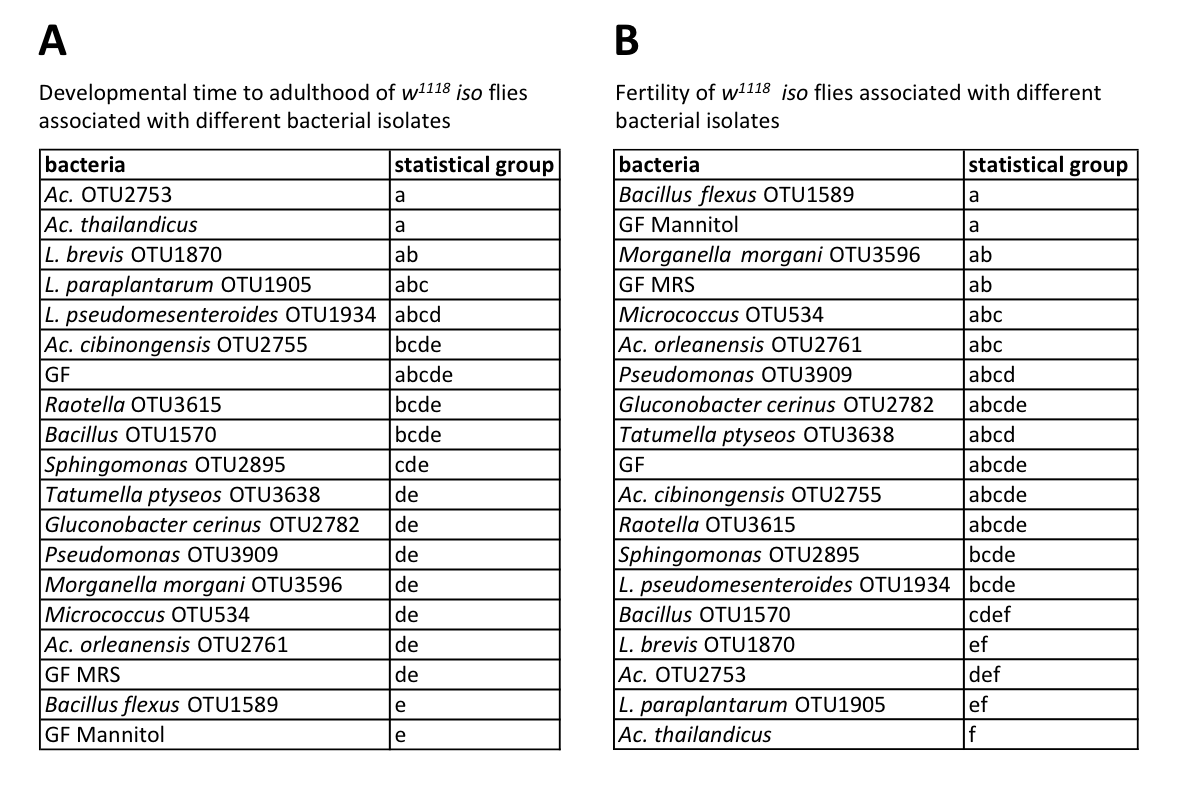

Supplement: S17 Fig — Developmental time to adulthood (A) and fertility (B) of flies associated with different bacterial isolates from S16 Fig were analyzed with Tukey’s pairwise comparisons on the lmm estimates. Statistical groups of significance were generated with cld function in R. Groups with the same letter are not significantly different from each other. Supporting data can be found in S21 and S22 Data. lmm, linear mixed model. (TIF) [file pbio.2005710.s017.tif]

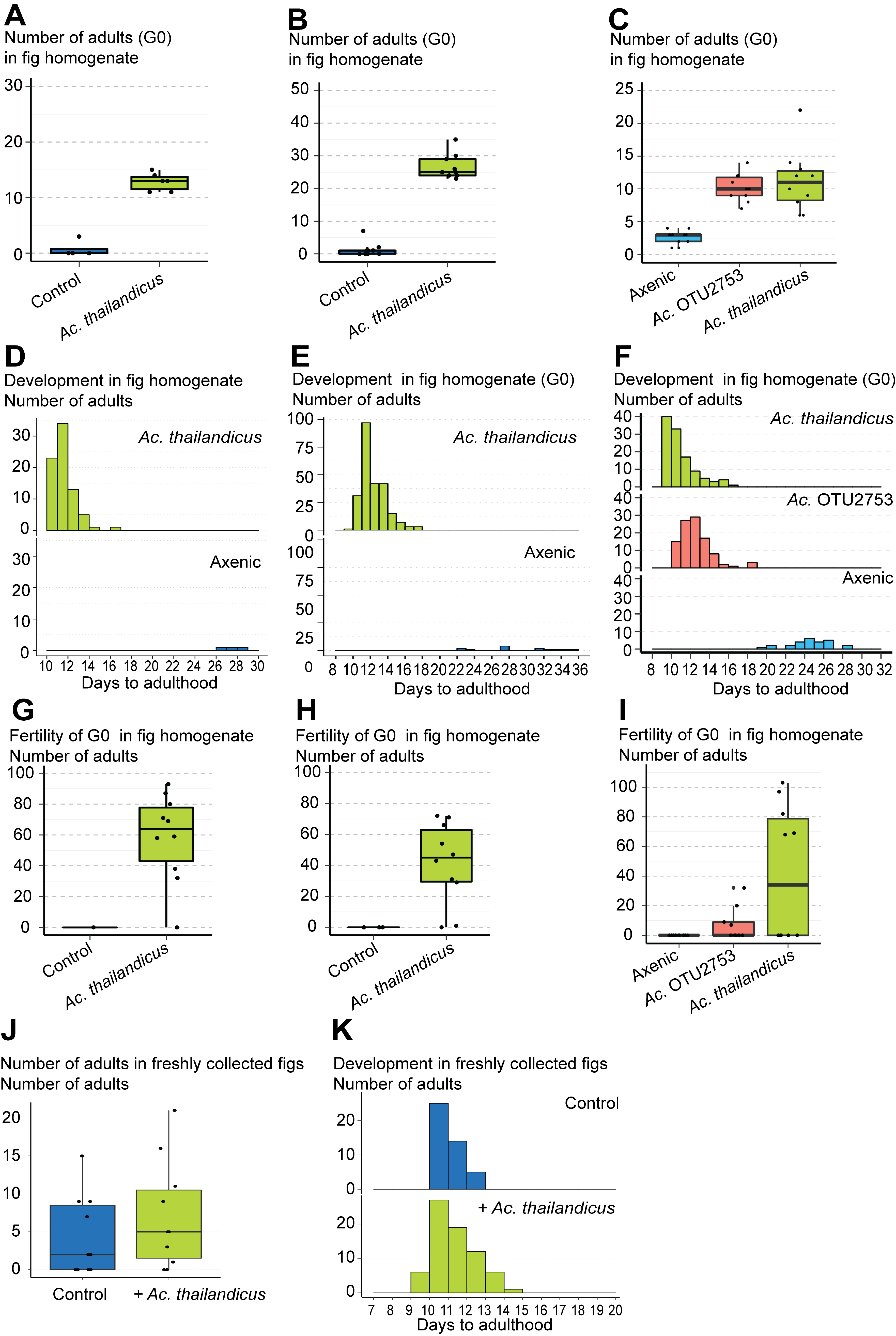

Supplement: S18 Fig — (A–F) Thirty axenic w1118 iso eggs were placed in vials containing sterilized fig homogenate. A. thailandicus, Acetobacter OTU2753, or sterile culture media were added on the top of the eggs. Four to ten vials were used per condition. Total number of adults that emerged (A–C) and developmental time to adulthood (D–F) were determined. More eggs inoculated with A. thailandicus and Acetobacter OTU2753 developed to adulthood, and faster than axenic eggs (lmm, p < 0.001 for both comparisons). Supporting data can be found in S23 Data. (G–I) Progeny of flies developed in fig homogenate with and without the addition of Acetobacter species. One male and one female were collected from G0 of each condition and placed per vial containing fig homogenate for 10 days, with vials flipped every other day. A. thailandicus and Acetobacter OTU2753 conditions have 10 replicates, but only 1 to 9 replicates from axenic eggs were possible to perform. Flies that were inoculated with A. thailandicus had higher progeny numbers than flies inoculated with Acetobacter OTU2753 or sterile media (lmm, p < 0.001). Supporting data can be found in S24 Data. (J, K) Fifty axenic w1118 iso eggs were placed in vials containing freshly collected nonsterile figs. A. thailandicus culture or sterile media (Control) was added on the top of the eggs. The total number of adults that emerged (J) and their developmental time to adulthood (K) were analyzed. Ten vials were analyzed per condition. There were more adults emerging from vials inoculated with A. thailandicus (lmm, p = 0.010). Developmental time to adulthood was not significantly different in this experimental replicate but was faster in eggs inoculated with A. thailandicus in the other replicate represented in Fig 7J (lmm, p = 0.557 and p < 0.001, respectively). Supporting data can be found in S25 Data. Statistical analyses were performed together with replicate experiments shown in Fig 7D–7J. lmm, linear mixed model; w1118 iso, w1118 DrosDel isogenic st [file pbio.2005710.s018.tif]
