# Supplementary material for: Drosophila melanogaster establishes a species-specific mutualistic interaction with stable gut-colonizing bacteria
Source: PLoS Biol. 2018 Jul 5;16(7):e2005710. doi: 10.1371/journal.pbio.2005710 (PMC6049943; doi:10.1371/journal.pbio.2005710)
Supplement: S1 Text — Sequence obtained by amplifying the gene with the primers 27F and 1495r. Code corresponds to code of laboratory isolate. Results of analysis on Greengenes, and of the BLAST analysis against the NCBI 16S rRNA sequences (Bacteria and Archaea) database are also shown. (DOCX) [file pbio.2005710.s019.docx]

| Code | B_00376/11 |
| --- | --- |
| Greengenes taxonomy | *Leuconostoc pseudomesenteroides* OTU1934 |
| Greengenes ID | 100% |
| NCBI best match | *Leuconostoc pseudomesenteroides* |
| NCBI ID | 100% |
| NCBI accession nº. | MG808351.1 |
| Sequence 16S | ACCTTTCAAGTGAGTGGCGAACGGGTGAGTAACACGTGGACAACCTGCCTCAAGGCTGGGGATAACATTTGGAAACAGATGCTAATACCGAATAAAACTCAGTGTCGCATGACACAAAGTTAAAAGGCGCTTTGGCGTCACCTAGAGATGGATCCGCGGTGCATTAGTTAGTTGGTGGGGTAAAGGCCTACCAAGACAATGATGCATAGCCGAGTTGAGAGACTGATCGGCCACATTGGGACTGAGACACGGCCCAAACTCCTACGGGAGGCTGCAGTAGGGAATCTTCCACAATGGGCGAAAGCCTGATGGAGCAACGCCGCGTGTGTGATGAAGGCTTTCGGGTCGTAAAGCACTGTTGTACGGGAAGAACAGCTAGAATAGGGAATGATTTTAGTTTGACGGTACCATACCAGAAAGGGACGGCTAAATACGTGCCAGCAGCCGCGGTAATACGTATGTCCCGAGCGTTATCCGGATTTATTGGGCGTAAAGCGAGCGCAGACGGTTGATTAAGTCTGATGTGAAAGCCCGGAGCTCAACTCCGGAATGGCATTGGAAACTGGTTAACTTGAGTGCAGTAGAGGTAAGTGGAACTCCATGTGTAGCGGTGGAATGCGTAGATATATGGAAGAACACCAGTGGCGAAGGGCGGCTTACTGGACTGTAACTGACGTTGAGGCTCGAAAGGTGTGGGTAGCAAACAGGATTAGATACCCTGGTAGTCCACACCGTAAACGATGAACCACTAGGTGTTAGGAGGTTTCCGCCTCTTAGTGCCGAAGCTAACGCATTAAGTGTTCCGCCTGGGGAGTACGACCGCAAGGTTGAAACTCAAAGGAATTGACGGGGACCCGCACAAGCGGTGGAGCATGTGGTTTAATTCGAAGCAACGCGAAGAACCTTACCAGGTCTTGACATCCTTTGAAGCTTTTAGAGATAGAAGTGTTCTCTTCGGAGACAAAGTGACAGGTGGTGCATGGTCGTCGTCAGCTCGTGTCGTGAGATGTTGGGTTAAGTCCCGCAACGAGCGCAACCCTTATTGTTAGTTGCCAGCATTCAGATGGGCACTCTAGCGAGACTGCCGGTGACAAACCGGAGGAAGGCGGGGNCGACGTCAGATCATCATGCCCCTTATGACCTGGGCTACACACGTGCTACAATGGCGTATACAACGAGTTGCCAACCCGCGAGGGTGAGCTAATCTCTTAAAGTACGTCTCAGTTCGGATTGTAGTCTGCAACTCGACTACATGAAGTCGGAATCGCTAGTAATCGCGGATCAGCACGCCGCGGTGAATACGTTCCCGGGTCTTGTACACACCGCCCGTCAC |

| Code | B_00356/12 |
| --- | --- |
| Greengenes taxonomy | *Lactobacillus brevis* OTU1870 |
| Greengenes ID | 99% |
| NCBI best match | *Lactobacillus brevis* |
| NCBI ID | 99% |
| NCBI accession nº. | MG808350.1 |
| Sequence 16S | TGCTTGCACTGATTTCAACATTGAAGCGAGTGGCGAACTGGTGAGTAACACGTGGGAAATCTGCCCAGAAGCAGGGGATAACACTTGGAAACAGGTGCTAATACCGTATAACAACAAAATCCGCATGGATTTTGTTTGAAAGGTGGCTTCGGCTATCACTTCTGGATGATCCCGCGGCGTATTAGTTAGTTGGTGAGGTAAAGGCCCACCAAGACGATGATACGTAGCCGACCTGAGAGGGTAATCGGCCACATTGGGACTGAGACACGGCCCAAACTCCTACGGGAGGCAGCAGTAGGGAATCTTCCACAATGGACGAAAGTCTGATGGAGCAATGCCGCGTGAGTGAAGAAGGGTTTCGGCTCGTAAAACTCTGTTGTTAAAGAAGAACACCTTTGAGAGTAACTGTTCAAGGGTTGACGGTATTTAACCAGAAAGCCACGGCTAACTACGTGCCAGCAGCCGCGGTAATACGTAGGTGGCAAGCGTTGTCCGGATTTATTGGGCGTAAAGCGAGCGCAGGCGGTTTTTTAAGTCTGATGTGAAAGCCTTCGGCTTAACCGGAGAAGTGCATCGGAAACTGGGAGACTTGAGTGCAGAAGAGGACAGTGGAACTCCATGTGTAGCGGTGGAATGCGTAGATATATGGAAGAACACCAGTGGCGAAGGCGGCTGTCTAGTCTGTAACTGACGCTGAGGCTCGAAAGCATGGGTAGCGAACAGGATTAGATACCCTGGTAGTCCATGCCGTAAACGATGAGTGCTAAGTGTTGGAGGGTTTCCGCCCTTCAGTGCTGCAGCTAACGCATTAAGCACTCCGCCTGGGGAGTACGACCGCAAGGTTGAAACTCAAAGGAATTGACGGGGGCCCGCACAAGCGGTGGAGCATGTGGTTTAATTCGAAGCTACGCGAAGAACCTTACCAGGTCTTGACATCTTCTGCCAATCTTAGAGATAAGACGTTCCCTTCGGGGACAGAATGACAGGTGGTGCATGGTTGTCGTCAGCTCGTGTCGTGAGATGTTGGGTTAAGTCCCGCAACGAGCGCAACCCTTATTATCAGTTGCCAGCATTCAGTTGGGCACTCTGGTGAGACTGCCGGTGACAAACCGGAGGAAGGTGGGGATGACGTCAAATCATCATGCCCCTTATGACCTGGGCTACACACGTGCTACAATGGACGGTACAACGAGTCGCGAAGTCGTGAGGCTAAGCTAATCTCTTAAAGCCGTTCTCAGTTCGGATTGTAGGCTGCAACTCGCCTACATGAAGTTGGAATCGCTAGTAATCGCGGATCAGCATGCCGCGGTGAATACGTTCCCGGGCCTTGTACACACCGCCCGTCACACCATGAGAGTTTGAACACCCAAA |

| Code | B_00384/12 |
| --- | --- |
| Greengenes taxonomy | *Lactobacillus paraplantarum* OTU1870 |
| Greengenes ID | 99% |
| NCBI best match | *Lactobacillus plantarum and Lactobacillus pentosus* |
| NCBI ID | 100% both |
| NCBI accession nº. | MG808352.1 |
| Sequence 16S | AAGGTTACCCCACCGACTTTGGGTGTTACAAACTCTCATGGTGTGACGGGCGGTGTGTACAAGGCCCGGGAACGTATTCACCGCGGCATGCTGATCCGCGATTACTAGCGATTCCGACTTCATGTAGGCGAGTTGCAGCCTACAATCCGAACTGAGAATGGCTTTAAGAGATTAGCTTACTCTCGCGAGTTCGCAACTCGTTGTACCATCCATTGTAGCACGTGTGTAGCCCAGGTCATAAGGGGCATGATGATTTGACGTCATCCCCACCTTCCTCCGGTTTGTCACCGGCAGTCTCACCAGAGTGCCCAACTTAATGCTGGCAACTGATAATAAGGGTTGCGCTCGTTGCGGGACTTAACCCAACATCTCACGACACGAGCTGACGACAACCATGCACCACCTGTATCCATGTCCCCGAAGGGAACGTCTAATCTCTTAGATTTGCATAGTATGTCAAGACCTGGTAAGGTTCTTCGCGTAGCTTCGAATTAAACCACATGCTCCACCGCTTGTGCGGGCCCCCGTCAATTCCTTTGAGTTTCAGCCTTGCGGCCGTACTCCCCAGGCGGAATGCTTAATGCGTTAGCTGCAGCACTGAAGGGCGGAAACCCTCCAACACTTAGCATTCATCGTTTACGGTATGGACTACCAGGGTATCTAATCCTGTTTGCTACCCATACTTTCGAGCCTCAGCGTCAGTTACAGACCAGACAGCCGCCTTCGCCACTGGTGTTCTTCCATATATCTACGCATTTCACCGCTACACATGGAGTTCCACTGTCCTCTTCTGCACTCAAGTTTCCCAGTTTCCGATGCACTTCTTCGGTTGAGCCGAAGGCTTTCACATCAGACTTAAAAAACCGCCTGCGCTCGCTTTACGCCCAATAAATCCGGACAACGCTTGCCACCTACGTATTACCGCGGCTGCTGGCACGTAGTTAGCCGTGGCTTTCTGGTTAAATACCGTCAATACCTGAACAGTTACTCTCAGATATGTTCTTCTTTAACAACAGAGTTTTACGAGCCGAAACCCTTCTTCACTCACGCGGCGTTGCTCCATCAGACTTTCGTCCATTGTGGAAGATTCCCTACTGCTGCCTCCCGTAGGAGTTTGGGCCGTGTCTCAGTCCCAATGTGGCCGATTACCCTCTCAGGTCGGCTACGTATCATTGCCATGGTGAGCCGTTACCCCACCATCTAGCTAATACGCCGCGGGACCATCCAAAAGTGATAGCCGAAGCCATCTTTCAAACTCGGACCATGCGGTCCAAGTTGTTATGCGGTATTAGCATCTGTTTCCAGGTGTTATCCCCCGCTTCTGGGCAGGTTTCCCACGTGTTACTCACCAGTTCGCCACTCACTCAAATGTAAATCATGATGCAAGCACCAA |

| Code | B_00466/12 |
| --- | --- |
| Greengenes taxonomy | *Morganella morganii* OTU3596 |
| Greengenes ID | 99% |
| NCBI best match | *Morganella morganii* |
| NCBI ID | 99% |
| NCBI accession nº. | MG808353.1 |
| Sequence 16S | TTCTCTGCTGACGAGCGGCGGACGGGTGAGTAATGTATGGGGATCTGCCTGATGGCGGGGGATAACTACTGGAAACGGTAGCTAATACCGCATAATGTCTTCGGACCAAAGCGGGGGACCTTCGGGCCTCGCGCCATCAGATGAACCCATATGGGATTAGCTGGTAGGTGGGGTAACGGCTCACCTAGGCGACGATCCCTAGCTGGTCTGAGAGGATGATCAGCCACACTGGGACTGAGACACGGCCCAGACTCCTACGGGAGGCAGCAGTGGGGAATATTGCACAATGGGCGCAAGCCTGATGCAGCCATGCCGCGTGTATGAAGAAGGCCTTCGGGTTGTAAAGTACTTTCAGTCGGGAGGAAGGTGGTGAGCTTAATACGCTCATCAATTGACGTTACCGACAGAAGAAGCACCGGCTAACTCCGTGCCAGCAGCCGCGGTAATACGGAGGGTGCAAGCGTTAATCGGAATTACTGGGCGTAAAGCGCACGCAGGCGGTTGATTGAGTCAGATGTGAAATCCCCGGGCTTAACCCGGGAATTGCATCTGATACTGGTCAGCTAGAGTCTTGTAGAGGGGGGTAGAATTCCATGTGTAGCGGTGAAATGCGTAGAGATGTGGAGGAATACCGGTGGCGAAGGCGGCCCCCTGGACAAAGACTGACGCTCAGGTGCGAAAGCGTGGGGAGCAAACAGGATTAGATACCCTGGTAGTCCACGCTGTAAACGATGTCGACTTGGAGGTTGTGCCCTTGAGGCGTGGCTTCCGGAGCTAACGCGTTAAGTCGACCGCCTGGGGAGTACGGCCGCAAGGTTAAAACTCAAATGAATTGACGGGGGCCCGCACAAGCGGTGGAGCATGTGGTTTAATTCGATGCAACGCGAAGAACCTTACCTACTCTTGACATCCAGAGAACTTGGCAGAGATGCTTTGGTGCCTTCGGGAACTCTGAGACAGGTGCTGCATGGCTGTCGTCAGCTCGTGTTGTGAAATGTTGGGTTAAGTCCCGCAACGAGCGCAACCCTTATCCTTTGTTGCCAGCGCGTGATGGCGGGAACTCAAAGGAGACTGCCGGTGATAAACCGGAGGAAGGTGGGGATGACGTCAAGTCATCATGGCCCTTACGAGTAGGGCTACACACGTGCTACAATGGCGTATACAAAGGGAAGCGACCCTGCGAAGGCAAGCGGAACTCATAAAGTACGTCGTAGTCCGGATTGGAGTCTGCAACTCGACTCCATGAAGTCGGAATCGCTAGTAATCGTAGATCAGAATGCTACGGTGAATACGTTCCCGGGCCTTGTACACACCGCCCGTCACACCATGGGAGTGGGTTGCAAAAGA |

| Code | B_00487/12 |
| --- | --- |
| Greengenes taxonomy | *Ac. orleanensis* OTU2761 |
| Greengenes ID | 99% |
| NCBI best match | *Acetobacter malorum* |
| NCBI ID | 100% |
| NCBI accession nº. | MG808354.1 |
| Sequence 16S | GGACGGGTGAGTAACGCGTAGGAATCTATCCACGGGTGGGGGATAACTCCGGGAAACTGGAGCTAATACCGCATGATACCTGAGGGTCAAAGGCGCAAGTCGCCTGTGGAGGAGCCTGCGTTTGATTAGCTTGTTGGTGGGGTAATGGCCTACCAAGGCGATGATCAATAGCTGGTCTGAGAGGATGATCAGCCACACTGGGACTGAGACACGGCCCAGACTCCTACGGGAGGCAGCAGTGGGGAATATTGGACAATGGGGGCAACCCTGATCCAGCAATGCCGCGTGTGTGAAGAAGGTTTTCGGATTGTAAAGCACTTTCGGCGGGGACGATGATGACGGTACCCGCAGAAGAAGCCCCGGCTAACTTCGTGCCAGCAGCCGCGGTAATACGAAGGGGGCTAGCGTTGCTCGGAATGACTGGGCGTAAAGGGCGTGTAGGCGGTTTGTACAGTTAGATGTGAAATCCCCGGGCTTAACCTGGGAGCTGCATTTAATACGTGCAGACTAGAGTGTGAGAGAGGGTTGTGGAATTCCCAGTGTAGAGGTGAAATTCGTAGATATTGGGAAGAACACCGGTGGCGAAGGCGGCAACCTGGCTCATTACTGACGCTGAGGCGCGAAAGCGTGGGGAGCAAACAGGATTAGATACCCTGGTAGTCCACGCTGTAAACGATGTGTGCTGGATGTTGGGTAACTTAGTTACTCAGTGTCGTAGCTAACGCGATAAGCACACCGCCTGGGGAGTACGGCCGCAAGGTTGAAACTCAAAGGAATTGACGGGGGCCCGCACAAGCGGTGGAGCATGTGGTTTAATTCGAAGCAACGCGCAGAACCTTACCAGGGCTTGTATGGGTAGGCTGTGTCCAGAGATGGGCATTTCCCGCAAGGGACCTACCGCACAGGTGCTGCATGGCTGTCGTCAGCTCGTGTCGTGAGATGTTGGGTTAAGTCCCGCAACGAGCGCAACCCCTATCTTTAGTTGCCAGCATGTTTGGGTGGGCACTCTAGAGAGACTGCCGGTGACAAGCCGGAGGAAGGTGGGGATGACGTCAAGTCCTCATGGCCCTTATGTCCTGGGCTACACACGTGCTACAATGGCGATGACAATGGGAAGCTAGATGGCGACATCGTGCTGATCTCAAAAAGTCGTCTCAGTTCGGATTGCACTCTGCAACTCGAGTGCATGAAGGTGGAATCGCTAGTAATCGCGGATCAGCATGCCGCGGTGAATACGTTCCCGGGCCTTGTACACACCGCCCGTCACACCATGGGAGTTGGTTTGACCTTAAGCC |

| Code | B_00539/12 |
| --- | --- |
| Greengenes taxonomy | *Micrococcus luteus* OTU533 |
| Greengenes ID | 98% |
| NCBI best match | *Micrococcus yunnanensis* |
| NCBI ID | 99% |
| NCBI accession nº. | MG808355.1 |
| Sequence 16S | GATTAGTGGCGAACGGGTGAGTAACACGTGAGTAACCTGCCCTTAACTCTGGGATAAGCCTGGGAAACTGGGTCTAATACCGGATAGGAGCGTCCACCGCATGGTGGGTGTTGGAAAGATTTATCGGTTTTGGATGGACTCGCGGCCTATCAGCTTGTTGGTGAGGTAATGGCTCACCAAGGCGACGACGGGTAGCCGGCCTGAGAGGGTGACCGGCCACACTGGGACTGAGACACGGCCCAGACTCCTACGGGAGGCAGCAGTGGGGAATATTGCACAATGGGCGAAAGCCTGATGCAGCGACGCCGCGTGAGGGATGACGGCCTTCGGGTTGTAAACCTCTTTCAGTAGGGAAGAAGCGAAAGTGACGGTACCTGCAGAAGAAGCACCGGCTAACTACGTGCCAGCAGCCGCGGTAATACGTAGGGTGCGAGCGTTATCCGGAATTATTGGGCGTAAAGAGCTCGTAGGCGGTTTGTCGCGTCTGTCGTGAAAGTCCGGGGCTTAACCCCGGATCTGCGGTGGGTACGGGCAGACTAGAGTGCAGTAGGGGAGACTGGAATTCCTGGTGTAGCGGTGGAATGCGCAGATATCAGGAGGAACACCGATGGCGAAGGCAGGTCTCTGGGCTGTAACTGACGCTGAGGAGCGAAAGCATGGGGAGCGAACAGGATTAGATACCCTGGTAGTCCATGCCGTAAACGTTGGGCACTAGGTGTGGGGACCATTCCACGGTTTCCGCGCCGCAGCTAACGCATTAAGTGCCCCGCCTGGGGAGTACGGCCGCAAGGCTAAAACTCAAAGGAATTGACGGGGGCCCGCACAAGCGGCGGAGCATGCGGATTAATTCGATGCAACGCGAAGAACCTTACCAAGGCTTGACATGTTCTCGATCGCCGTAGAGATACGGTTTCCCCTTTGGGGCGGGTTCACAGGTGGTGCATGGTTGTCGTCAGCTCGTGTCGTGAGATGTTGGGTTAAGTCCCGCAACGAGCGCAACCCTCGTTCCATGTTGCCAGCACGTAGTGGTGGGGACTCATGGGAGACTGCCGGGGTCAACTCGGAGGAAGGTGAGGACGACGTCAAATCATCATGCCCCTTATGTCTTGGGCTTCACGCATGCTACAATGGCCGGTACAATGGGTTGCGATACTGTGAGGTGGAGCTAATCCCAAAAAGCCGGTCTCAGTTCGGATTGGGGTCTGCAACTCGACCCCATGAAGTCGGAGTCGCTAGTAATCGCAGATCAGCAACGCTGCGGTGAATACGTTCCCGGGCCTTGTACACACCGCCCGTCAAGTCACGAAAGTTGGTAACACCCGAAGCC |

| Code | B_00600/12 |
| --- | --- |
| Greengenes taxonomy | *Pseudomonas* Unclassified OTU3909 |
| Greengenes ID | 100% |
| NCBI best match | *Pseudomonas oryzihabitans, Pseudomonas psychrotolerans,* |
| NCBI ID | 99% both |
| NCBI accession nº. | MG808356.1 |
| Sequence 16S | GCTCCTCGATTCAGCGGCGGACGGGTGAGTAATGCCTAGGAATCTGCCTAGTAGTGGGGGACAACGTTTCGAAAGGAACGCTAATACCGCATACGTCCTACGGGAGAAAGTGGGGGATCTTCGGACCTCACGCTATTAGATGAGCCTAGGTCGGATTAGCTAGTTGGTAGGGTAAAGGCCTACCAAGGCGACGATCCGTAACTGGTCTGAGAGGATGATCAGTCACACTGGAACTGAGACACGGTCCAGACTCCTACGGGAGGCAGCAGTGGGGAATATTGGACAATGGGCGAAAGCCTGATCCAGCCATGCCGCGTGTGTGAAGAAGGCCCTCGGGTCGTAAAGCACTTTAAGTTGGGAGGAAGGGCTTACAGCGAATACCTGTGAGTTTTGACGTTACCGACAGAATAAGCACCGGCTAACTTCGTGCCAGCAGCCGCGGTAATACGAAGGGTGCAAGCGTTAATCGGAATTACTGGGCGTAAAGCGCGCGTAGGTGGCTTGATAAGTTGGATGTGAAATCCCCGGGCTCAACCTGGGAACTGCATCCAAAACTGTCTGGCTAGAGTGCGGTAGAGGGTAGTGGAATTTCCAGTGTAGCGGTGAAATGCGTAGATATTGGAAGGAACACCAGTGGCGAAGGCGACTACCTGGACTGACACTGACACTGAGGTGCGAAAGCGTGGGGAGCAAACAGGATTAGATACCCTGGTAGTCCACGCCGTAAACGATGTCAACTAGCCGTTGGGATCCTTGAGATCTTAGTGGCGCAGCTAACGCATTAAGTTGACCGCCTGGGGAGTACGGCCGCAAGGTTAAAACTCAAATGAATTGACGGGGGCCCGCACAAGCGGTGGAGCATGTGGTTTAATTCGAAGCAACGCGAAGAACCTTACCTGGCCTTGACATGCTGAGAACTTTCCAGAGATGGATTGGTGCCTTCGGGAACTCAGACACAGGTGCTGCATGGCTGTCGTCAGCTCGTGTCGTGAGATGTTGGGTTAAGTCCCGTAACGAGCGCAACCCTTGTCCTTAGTTACCAGCACGTTATGGTGGGCACTCTAAGGAGACTGCCGGTGACAAACCGGAGGAAGGTGGGGATGACGTCAAGTCATCATGGCCCTTACGGCCAGGGCTACACACGTGCTACAATGGTCGGTACAAAGGGTTGCCAAGCCGCGAGGTGGAGCTAATCCCATAAAACCGATCGTAGTCCGGATCGCAGTCTGCAACTCGACTGCGTGAAGTCGGAATCGCTAGTAATCGTGAATCAGAACGTCACGGTGAATACGTTCCCGGGCCTTGTACACACCGCCCGTCACACCATGGGAGTGGGTTGCTCCAGA |

| Code | B_00626/12 |
| --- | --- |
| Greengenes taxonomy | *Bacillus flexus* OTU1589 |
| Greengenes ID | 99% |
| NCBI best match | *Bacillus aryabhattai* |
| NCBI ID | 99% |
| NCBI accession nº. | MG808357.1 |
| Sequence 16S | GCTTCTATGACGTTAGCGGCGGACGGGTGAGTAACACGTGGGCAACCTGCCTGTAAGACTGGGATAACTTCGGGAAACCGAAGCTAATACCGGATAGGATCTTCTCCTTCATGGGAGATGATTGAAAGATGGTTTCGGCTATCACTTACAGATGGGCCCGCGGTGCATTAGCTAGTTGGTGAGGTAACGGCTCACCAAGGCAACGATGCATAGCCGACCTGAGAGGGTGATCGGCCACACTGGGACTGAGACACGGCCCAGACTCCTACGGGAGGCAGCAGTAGGGAATCTTCCGCAATGGACGAAAGTCTGACGGAGCAACGCCGCGTGAGTGATGAAGGCTTTCGGGTCGTAAAACTCTGTTGTTAGGGAAGAACAAGTACGAGAGTAACTGCTCGTACCTTGACGGTACCTAACCAGAAAGCCACGGCTAACTACGTGCCAGCAGCCGCGGTAATACGTAGGTGGCAAGCGTTATCCGGAATTATTGGGCGTAAAGCGCGCGCAGGCGGTTTCTTAAGTCTGATGTGAAAGCCCACGGCTCAACCGTGGAGGGTCATTGGAAACTGGGGAACTTGAGTGCAGAAGAGAAAAGCGGAATTCCACGTGTAGCGGTGAAATGCGTAGAGATGTGGAGGAACACCAGTGGCGAAGGCGGCTTTTTGGTCTGTAACTGACGCTGAGGCGCGAAAGCGTGGGGAGCAAACAGGATTAGATACCCTGGTAGTCCACGCCGTAAACGATGAGTGCTAAGTGTTAGAGGGTTTCCGCCCTTTAGTGCTGCAGCTAACGCATTAAGCACTCCGCCTGGGGAGTACGGTCGCAAGACTGAAACTCAAAGGAATTGACGGGGGCCCGCACAAGCGGTGGAGCATGTGGTTTAATTCGAAGCAACGCGAAGAACCTTACCAGGTCTTGACATCCTCTGACAACTCTAGAGATAGAGCGTTCCCCTTCGGGGGACAGAGTGACAGGTGGTGCATGGTTGTCGTCAGCTCGTGTCGTGAGATGTTGGGTTAAGTCCCGCAACGAGCGCAACCCTTGATCTTAGTTGCCAGCATTTAGTTGGGCACTCTAAGGTGACTGCCGGTGACAAACCGGAGGAAGGTGGGGATGACGTCAAATCATCATGCCCCTTATGACCTGGGCTACACACGTGCTACAATGGATGGTACAAAGGGCTGCAAGACCGCGAGGTCAAGCCAATCCCATAAAACCATTCTCAGTTCGGATTGTAGGCTGCAACTCGCCTACATGAAGCTGGAATCGCTAGTAATCGCGGATCAGCATGCCGCGGTGAATACGTTCCCGGGCCTTGTACACACCGCCCGTCACACCACGAGAGTTTGAACACCCGAAG |

| Code | B_00708/12 |
| --- | --- |
| Greengenes taxonomy | *Tatumella ptyseos* OTU3638 |
| Greengenes ID | 99% |
| NCBI best match | *Tatumella punctata* |
| NCBI ID | 99% |
| NCBI accession nº. | MG808358.1 |
| Sequence 16S | CTTGCTCCTTGGGTGACGAGTGGCGGACGGGTGAGTAATGTCTGGGAAACTGCCTGATGGAGGGGGATAACTACTGGAAACGGTAGCTAATACCGCATAATGTCGCAAGACCAAAGCGGGGGACCTTCGGGCCTCGCACCATCGGATGTGCCCAGATGGGATTAGCTAGTAGGTGGGGTAATGGCTCACCTAGGCGACGATCCCTAGCTGGTCTGAGAGGATGACCAGCCACACTGGAACTGAGACACGGTCCAGACTCCTACGGGAGGCAGCAGTGGGGAATATTGCACAATGGGCGCAAGCCTGATGCAGCCATGCCGCGTGTATGAAGAAGGCCTTCGGGTTGTAAAGTACTTTCAGCGGGGAGGAAGGCATTGCGGTTAATAACCGCAGTGATTGACGTTACCCGCAGAAGAAGCACCGGCTAACTCCGTGCCAGCAGCCGCGGTAATACGGAGGGTGCAAGCGTTAATCGGAATTACTGGGCGTAAAGCGCACGCAGGCGGTCTGTCAAGTCGGATGTGAAATCCCCGGGCTCAACCCGGGAACTGCATTCGAAACTGGCAGACTAGAGTCTTGTAGAGGGGGGTAGAATTCCAGGTGTAGCGGTGAAATGCGTAGAGATCTGGAGGAATACCGGTGGCGAAGGCGGCCCCCTGGACAAAGACTGACGCTCAGGTGCGAAAGCGTGGGGAGCAAACAGGATTAGATACCCTGGTAGTCCACGCCGTAAACGATGTCGACTTGGAGGCTGTGCCCTTGAGGCGTGGCTTCCGGAGCTAACGCGTTAAGTCGACCGCCTGGGGAGTACGGCCGCAAGGTTAAAACTCAAATGAATTGACGGGGGCCCGCACAAGCGGTGGAGCATGTGGTTTAATTCGATGCAACGCGAAGAACCTTACCTACTCTTGACATCCAGAGAACTTAGCAGAGATGCTTTGGTGCCTTCGGGAACTCTGAGACAGGTGCTGCATGGCTGTCGTCAGCTCGTGTTGTGAAATGTTGGGTTAAGTCCCGCAACGAGCGCAACCCCTATCCTTTGTTGCCAGCGATTCGGTCGGGAACTCAAAGGAGACTGCCGGTGATAAACCGGAGGAAGGTGGGGATGACGTCAAGTCATCATGGCCCTTACGAGTAGGGCTACACACGTGCTACAATGGCGCATACAAAGAGAAGCGACCTCGCGAGAGCAAGCGGACCTCATAAAGTGCGTCGTAGTCCGGATTGGAGTCTGCAACTCGACTCCATGAAGTCGGAATCGCTAGTAATCGTAGATCAGAATGCTACGGTGAATACGTTCCCGGGCCTTGTACACACCGCCCGTCACACCATGGGAGTGGGTTGCAAA |

| Code | B_00764/12 |
| --- | --- |
| Greengenes taxonomy | *Gluconobacter cerinus* OTU2782 |
| Greengenes ID | 99% |
| NCBI best match | *Gluconobacter cerinus* |
| NCBI ID | 99% |
| NCBI accession nº. | MG808359.1 |
| Sequence 16S | CGGACGGGTGAGTAACGCGTAGGGATCTATCCACGGGTGGGGGACAACTCCGGGAAACTGGAGCTAATACCGCATGATACCTGAGGGTCAAAGGCGCAAGTCGCCTGTGGAGGAACCTGCGTTCGATTAGCTAGTTGGTGGGGTAAAGGCCTACCAAGGCGATGATCGATAGCTGGTTTGAGAGGATGATCAGCCACACTGGGACTGAGACACGGCCCAGACTCCTACGGGAGGCAGCAGTGGGGAATATTGGACAATGGGCGAAAGCCTGATCCAGCAATGCCGCGTGTGTGAAGAAGGTCTTCGGATTGTAAAGCACTTTCGACGGGGACGATGATGACGGTACCCGTAGAAGAAGCCCCGGCTAACTTCGTGCCAGCAGCCGCGGTAATACGAAGGGGGCTAGCGTTGCTCGGAATGACTGGGCGTAAAGGGCGCGTAGGCGGTTTATGCAGTCAGATGTGAAATCCCCGGGCTTAACCTGGGAACTGCATTTGAGACGCATAGACTAGAGGTCGAGAGAGGGTTGTGGAATTCCCAGTGTAGAGGTGAAATTCGTAGATATTGGGAAGAACACCGGTGGCGAAGGCGGCAACCTGGCTCGATACTGACGCTGAGGCGCGAAAGCGTGGGGAGCAAACAGGATTAGATACCCTGGTAGTCCACGCTGTAAACGATGTGTGCTGGATGTTGGGTAACTTAGTTACTCAGTGTCGAAGCTAACGCGCTAAGCACACCGCCTGGGGAGTACGGCCGCAAGGTTGAAACTCAAAGGAATTGACGGGGGCCCGCACAAGCGGTGGAGCATGTGGTTTAATTCGAAGCAACGCGCAGAACCTTACCAGGACTTGCATGGGGAGGACGTACTCAGAGATGGGTATTTCTTCGGACCTCCCGCACAGGTGCTGCATGGCTGTCGTCAGCTCGTGTCGTGAGATGTTGGGTTAAGTCCCGCAACGAGCGCAACCCTTGTCTTTAGTTGCCAGCACTTTCAGGTGGGCACTCTAGAGAGACTGCCGGTGACAAGCCGGAGGAAGGTGGGGATGACGTCAAGTCCTCATGGCCCTTATGTCCTGGGCTACACACGTGCTACAATGGCGGTGACAGTGGGAAGCTACATGGCGACATGGTGCTGATCTCTAAAAGCCGTCTCAGTTCGGATTGTACTCTGCAACTCGAGTACATGAAGGTGGAATCGCTAGTAATCGCGGATCAGCATGCCGCGGTGAATACGTTCCCGGGCCTTGTACACACCGCCCGTCACACCATGGGAGTTGGTTCGACCTTAA |

| Code | B_00977/12 |
| --- | --- |
| Greengenes taxonomy | *Bacillus* Unclassified OTU1570 |
| Greengenes ID | 99% |
| NCBI best match | *Bacillus safensis* |
| NCBI ID | 100% |
| NCBI accession nº. | MG808360.1 |
| Sequence 16S | CTCCCGGATGTTAGCGGCGGACGGGTGAGTAACACGTGGGTAACCTGCCTGTAAGACTGGGATAACTCCGGGAAACCGGAGCTAATACCGGATAGTTCCTTGAACCGCATGGTTCAAGGATGAAAGACGGTTTCGGCTGTCACTTACAGATGGACCCGCGGCGCATTAGCTAGTTGGTGGGGTAATGGCTCACCAAGGCGACGATGCGTAGCCGACCTGAGAGGGTGATCGGCCACACTGGGACTGAGACACGGCCCAGACTCCTACGGGAGGCAGCAGTAGGGAATCTTCCGCAATGGACGAAAGTCTGACGGAGCAACGCCGCGTGAGTGATGAAGGTTTTCGGATCGTAAAGCTCTGTTGTTAGGGAAGAACAAGTGCGAGAGTAACTGCTCGCACCTTGACGGTACCTAACCAGAAAGCCACGGCTAACTACGTGCCAGCAGCCGCGGTAATACGTAGGTGGCAAGCGTTGTCCGGAATTATTGGGCGTAAAGGGCTCGCAGGCGGTTTCTTAAGTCTGATGTGAAAGCCCCCGGCTCAACCGGGGAGGGTCATTGGAAACTGGGAAACTTGAGTGCAGAAGAGGAGAGTGGAATTCCACGTGTAGCGGTGAAATGCGTAGAGATGTGGAGGAACACCAGTGGCGAAGGCGACTCTCTGGTCTGTAACTGACGCTGAGGAGCGAAAGCGTGGGGAGCGAACAGGATTAGATACCCTGGTAGTCCACGCCGTAAACGATGAGTGCTAAGTGTTAGGGGGTTTCCGCCCCTTAGTGCTGCAGCTAACGCATTAAGCACTCCGCCTGGGGAGTACGGTCGCAAGACTGAAACTCAAAGGAATTGACGGGGGCCCGCACAAGCGGTGGAGCATGTGGTTTAATTCGAAGCAACGCGAAGAACCTTACCAGGTCTTGACATCCTCTGACAACCCTAGAGATAGGGCTTTCCCTTCGGGGACAGAGTGACAGGTGGTGCATGGTTGTCGTCAGCTCGTGTCGTGAGATGTTGGGTTAAGTCCCGCAACGAGCGCAACCCTTGATCTTAGTTGCCAGCATTCAGTTGGGCACTCTAAGGTGACTGCCGGTGACAAACCGGAGGAAGGTGGGGATGACGTCAAATCATCATGCCCCTTATGACCTGGGCTACACACGTGCTACAATGGACAGAACAAAGGGCTGCAAGACCGCAAGGTTTAGCCAATCCCATAAATCTGTTCTCAGTTCGGATCGCAGTCTGCAACTCGACTGCGTGAAGCTGGAATCGCTAGTAATCGCGGATCAGCATGCCGCGGTGAATACGTTCCCGGGCCTTGTACACACCGCCCGTCACACCACGAGAGTTTGCAACACCCG |

| Code | B_01114/12 |
| --- | --- |
| Greengenes taxonomy | *Acetobacter cibinongensis* OTU2755 |
| Greengenes ID | 100% |
| NCBI best match | *Acetobacter cibinongensis* |
| NCBI ID | 100% |
| NCBI accession nº. | MG808361.1 |
| Sequence 16S | TTTCGGCCTTAGTGGCGGACGGGTGAGTAACGCGTAGGAATCTATCCATGGGTGGGGGATAACTCTGGGAAACTGGAGCTAATACCGCATGACACCTTAGGGTCAAAGGCGCAAGTCGCCTGTGGAGGAGCCTGCGTTTGATTAGCTTGTTGGTGGGGTAATGGCCTACCAAGGCGATGATCAATAGCTGGTCTGAGAGGATGATCAGCCACACTGGGACTGAGACACGGCCCAGACTCCTACGGGAGGCAGCAGTGGGGAATATTGGACAATGGGGGCAACCCTGATCCAGCAATGCCGCGTGTGTGAAGAAGGTTTTCGGATTGTAAAGCACTTTCGGCGGGGACGATGATGACGGTACCCGCAGAAGAAGCCCCGGCTAACTTCGTGCCAGCAGCCGCGGTAATACGAAGGGGGCTAGCGTTGCTCGGAATGACTGGGCGTAAAGGGCGTGTAGGCGGTTTGTACAGTCAGATGTGAAATCCCCGGGCTTAACCTGGGAGCTGCATTTGATACGTGCAGACTAGAGTGTGAGAGAGGGTTGTGGAATTCCCAGTGTAGAGGTGAAATTCGTAGATATTGGGAAGAACACCGGTGGCGAAGGCGGCAACCTGGCTCATAACTGACGCTGAGGCGCGAAAGCGTGGGGAGCAAACAGGATTAGATACCCTGGTAGTCCACGCTGTAAACGATGTGTGCTAGATGTTGGGTAACTTAGTTATTCAGTGTCGCAGTTAACGCGTTAAGCACACCGCCTGGGGAGTACGGCCGCAAGGTTGAAACTCAAAGGAATTGACGGGGGCCCGCACAAGCGGTGGAGCATGTGGTTTAATTCGAAGCAACGCGCAGAACCTTACCAGGGCTTGTATGGGTAGGCTGTATCCAGAGATGGATATTTCCCGCAAGGGACCTACCGCACAGGTGCTGCATGGCTGTCGTCAGCTCGTGTCGTGAGATGTTGGGTTAAGTCCCGCAACGAGCGCAACCCCTATCTTTAGTTGCCAGCATGTTTGGGTGGGCACTCTAGAGAGACTGCCGGTGACAAGCCGGAGGAAGGTGGGGATGACGTCAAGTCCTCATGGCCCTTATGCCCTGGGCTACACACGTGCTACAATGGCGGTGACAGTGGGAAGCTAGATGGCGACATTGTGCTGATCTCTAAAAACCGTCTCAGTTCGGATTGCACTCTGCAACTCGAGTGCATGAAGGTGGAATCGCTAGTAATCGCGGATCAGCATGCCGCGGTGAATACGTTCCCGGGCCTTGTACACACCGCCCGTCACACCATGGGAGTTGGTTTGACCTTAAGCCGGTGAGCGAACCCGCAAAGGGGCCGCAG |

| Code | B_01153/12 |
| --- | --- |
| Greengenes taxonomy | *Acetobacter cibinongensis* OTU2755*, Acetobacter indonesiensis* OTU2758*, Acetobacter orientalis* OTU2760 |
| Greengenes ID | 98% |
| NCBI best match | *Acetobacter thailandicus* |
| NCBI ID | 99% |
| NCBI accession nº. | MG808362.1 |
| Sequence 16S | GCAGTCGCACGAAGGTTTCGGCCTTAGTGGCGGACGGGTGAGTAACGCGTAGGAATCTATCCACGGGTGGGGGATAACTTCGGGAAACTGAAGCTAATACCGCATGATACCTGAGGGTCAAAGGCGCAAGTCGCCTGTGGAGGAGCCTGCGTTTGATTAGCTTGTTGGTGGGGTAATGGCCTACCAAGGCGATGATCGATAGCTGGTTTGAGAGGATGATCAGCCACACTGGGACTGAGACACGGCCCAGACTCCTGCGGGAGGCAGCAGTGGGGAATATTGGACAATGGGGGCAACCCTGATCCAGCAATGCCGCGTGTGTGAAGAAGGTTTTCGGATTGTAAAGCACTTTCGGCGGGGACGATGATGACGGTACCCGCAGAAGAAGCCCCGGCTAACTTCGTGCCAGCAGCCGCGGTAATACGAAGGGGGCTAGCGTTGCTCGGAATGACTGGGCGTAAAGGGCGTGTAGGCGGTTTGTACAGTCAGATGTGAAATCCCCGGGCTTAACCTGGGAGCTGCATTTGATACGTGCAAACTAGAGTGTGAGAGAGGGTTGTGGAATTCCCAGTGTAGAGGTGAAATTCGTAGATATTGGGAAGAACACCGGTGGCGAAGGCGGCAACCTGGCTCATAACTGACGCTGAGGCGCGAAAGCGTGGGGAGCAAACAGGATTAGATACCCTGGTAGTCCACGCTGTAAACGATGTGTGCCAGATGTTGGGTAACTTAGTTATTCAGTGTCGTAGTTAACGCGTTAAGCACACCGCCTGGGGAGTACGGCCGCAAGGTTGAAACTCAAAGGAATTGACGGGGGCCCGCACAAGCGGTGGAGCATGTGGTTTAATTCGAAGCAACGCGCAGAACCTTACCAGGGCTTGAATGGGCAGGCCGTATCCAGAGATGGATATTTCCCGCAAGGGACCTGCCACACAGGTGCTGCATGGCTGTCGTCAGCTCGTGTCGTGAGATGTTGGGTTAAGTCCCGCAACGAGCGCAACCCTTATCTTTAGTTGCCAGCATGTTTGGGTGGGCACTCTAGAGAGACTGCCGGTGACAAGCCGGAGGAAGGTGGGGATGACGTCAAGTCCTCATGGCCCTTATGTCCTGGGCTACACACGTGCTACAATGGCGGTGACAGTGGGAAGCTAGATGGCGACATCGTGCTGATCTCTAAAAGCCGTCTCAGTTCGGATTGCACTCTGCAACTCGAGTGCATGAAGGTGGAATCGCTAGTAATCGCGGATCAGCATGCCGCGGTGAATACGTTCCCGGGCCTTGTACACACCGCCCGTCACACCATGGGAGTTGGTTTGACCTTAAGCCGGGAGCGAACCGCAAAGGGACGCAGCCG |

| Code | B_01485/12 |
| --- | --- |
| Greengenes taxonomy | *Raoutella* Unclassified OTU3615 |
| Greengenes ID | 99% |
| NCBI best match | *Obesumbacterium proteus* |
| NCBI ID | 99% |
| NCBI accession nº. | MG808363.1 |
| Sequence 16S | GCTTGCTCTCTGGGTGACGAGCGGCGGACGGGTGAGTAATGTCTGGGAAACTGCCTGATGGAGGGGGATAACTACTGGAAACGGTAGCTAATACCGCATGACGTCTTCGGACCAAAGTGGGGGACCTTCGGGCCTCACGCCATCAGATGTGCCCAGATGGGATTAGCTAGTAGGTGGGGTAATGGCTCACCTAGGCGACGATCTCTAGCTGGTCTGAGAGGATGACCAGCCACACTGGAACTGAGACACGGTCCAGACTCCTACGGGAGGCAGCAGTGGGGAATATTGCACAATGGGCGCAAGCCTGATGCAGCCATGCCGCGTGTATGAAGAAGGCCTTCGGGTTGTAAAGTACTTTCAGCGAGGAGGAAGGCATTGTGGTTAATAACCGCAGTGATTGACGTTACTCGCAGAAGAAGCACCGGCTAACTCCGTGCCAGCAGCCGCGGTAATACGGAGGGTGCAAGCGTTAATCGGAATTACTGGGCGTAAAGCGCACGCAGGCGGTTGATTAAGTCAGATGTGAAATCCCCGAGCTTAACTTGGGAACTGCATTTGAAACTGGTCAGCTAGAGTCTTGTAGAGGGGGGTAGAATTCCAGGTGTAGCGGTGAAATGCGTAGAGATCTGGAGGAATACCGGTGGCGAAGGCGGCCCCCTGGACAAAGACTGACGCTCAGGTGCGAAAGCGTGGGGAGCAAACAGGATTAGATACCCTGGTAGTCCACGCTGTAAACGATGTCGACTTGGAGGTTGTGCCCTTGAGGCGTGGCTTCCGGAGCTAACGCGTTAAGTCGACCGCCTGGGGAGTACGGCCGCAAGGTTAAAACTCAAATGAATTGACGGGGGCCCGCACAAGCGGTGGAGCATGTGGTTTAATTCGATGCAACGCGAAGAACCTTACCTACTCTTGACATCCAGAGAATTTGCTAGAGATAGCTTAGTGCCTTCGGGAACTCTGAGACAGGTGCTGCATGGCTGTCGTCAGCTCGTGTTGTGAAATGTTGGGTTAAGTCCCGCAACGAGCGCAACCCTTATCCTTTGTTGCCAGCACGTAATGGTGGGAACTCAAAGGAGACTGCCGGTGATAAACCGGAGGAAGGTGGGGATGACGTCAAGTCATCATGGCCCTTACGAGTAGGGCTACACACGTGCTACAATGGCATATACAAAGAGAAGCGAACTCGCGAGAGCAAGCGGACCTCATAAAGTATGTCGTAGTCCGGATTGGAGTCTGCAACTCGACTCCATGAAGTCGGAATCGCTAGTAATCGTAGATCAGAATGCTACGGTGAATACGTTCCCGGGCCTTGTACACACCGCCCGTCACACCATGGGAGTGGGTTGCAAAAG |

| Code | B_01504/12 |
| --- | --- |
| Greengenes taxonomy | *Sphingomonas* Unclassified OTU2895 |
| Greengenes ID | 99% |
| NCBI best match | *Sphingomonas panni* |
| NCBI ID | 99% |
| NCBI accession nº. | MG808364.1 |
| Sequence 16S | GTGGCGCACGGGTGCGTAACGCGTGGGAATCTGCCCTTAGGTTCGGAATAACAGTTAGAAATGACTGCTAATACCGGATGATGTCGATAAGACCAAAGATTTATCGCCTGAGGATGAGCCCGCGTAGGATTAGCTAGTTGGTGTGGTAAAGGCGCACCAAGGCGACGATCCTTAGCTGGTCTGAGAGGATGATCAGCCACACTGGGACTGAGACACGGCCCAGACTCCTACGGGAGGCAGCAGTGGGGAATATTGGACAATGGGCGCAAGCCTGATCCAGCAATGCCGCGTGAGTGATGAAGGCCTTAGGGTTGTAAAGCTCTTTTACCCGGGATGATAATGACAGTACCGGGAGAATAAGCCCCGGCTAACTCCGTGCCAGCAGCCGCGGTAATACGGAGGGGGCTAGCGTTGTTCGGAATTACTGGGCGTAAAGCGCACGTAGGCGGCTTTGTAAGTTAGAGGTGAAAGCCTGGAGCTCAACTCCAGAACTGCCTTTAAGACTGCATCGCTTGAATCCAGGAGAGGTGAGTGGAATTCCGAGTGTAGAGGTGAAATTCGTAGATATTCGGAAGAACACCAGTGGCGAAGGCGGCTCACTGGACTGGTATTGACGCTGAGGTGCGAAAGCGTGGGGAGCAAACAGGATTAGATACCCTGGTAGTCCACGCCGTAAACGATGATAACTAGCTGTCCGGGGACATGGTCTTTGGGTGGCGCAGCTAACGCATTAAGTTATCCGCCTGGGGAGTACGGTCGCAAGATTAAAACTCAAAGGAATTGACGGGGGCCTGCACAAGCGGTGGAGCATGTGGTTTAATTCGAAGCAACGCGCAGAACCTTACCAGCGTTTGACATGTCCGGACGACTTCTGGAGACAGATTTCTTCCCTTCGGGGACTGGAACACAGGTGCTGCATGGCTGTCGTCAGCTCGTGTCGTGAGATGTTGGGTTAAGTCCCGCAACGAGCGCAACCCTCGCCTTTAGTTACCATCATTTAGTTGGGTACTCTAAAGGAACCGCCGGTGATAAGCCGGAGGAAGGTGGGGATGACGTCAAGTCCTCATGGCCCTTACGCGCTGGGCTACACACGTGCTACAATGGCAGGTACAGTGGGCAGCAATCCCGCGAGGGTGAGCTAATCTCCAAAACCTGTCTCAGTTCGGATTGTTCTCTGCAACTCGAGAGCATGAAGGCGGAATCGCTAGTAATCGCGGATCAGCATGCCGCGGTGAATACGTTCCCAGGCCTTGTACACACCGCCCGTCACACCATGGGAGTTGGATTCACCCGA |

| Code | B_01959/12 |
| --- | --- |
| Greengenes taxonomy | *Acetobacter* Unclassified OTU2753 |
| Greengenes ID | 100% |
| NCBI best match | *Acetobacter pasteurianus* |
| NCBI ID | 100% |
| NCBI accession nº. | MG808365.1 |
| Sequence 16S | GGTTTTCGGCCTTAGTGGCGGACGGGTGAGTAACGCGTAGGTATCTATCCATGGGTGGGGGATAACACTGGGAAACTGGTGCTAATACCGCATGACACCTGAGGGTCAAAGGCGTAAGTCGCCTGTGGAGGAGCCTGCGTTTGATTAGCTAGTTGGTGGGGTAAAGGCCTACCAAGGCGATGATCAATAGCTGGTTTGAGAGGATGATCAGCCACACTGGGACTGAGACACGGCCCAGACTCCTACGGGAGGCAGCAGTGGGGAATATTGGACAATGGGGGCAACCCTGATCCAGCAATGCCGCGTGTGTGAAGAAGGTCTTCGGATTGTAAAGCACTTTCGACGGGGACGATGATGACGGTACCCGTAGAAGAAGCCCCGGCTAACTTCGTGCCAGCAGCCGCGGTAATACGAAGGGGGCTAGCGTTGCTCGGAATGACTGGGCGTAAAGGGCGTGTAGGCGGTTTGTACAGTCAGATGTGAAATCCCCGGGCTTAACCTGGGAGCTGCATTTGATACGTGCAGACTAGAGTGTGAGAGAGGGTTGTGGAATTCCCAGTGTAGAGGTGAAATTCGTAGATATTGGGAAGAACACCGGTGGCGAAGGCGGCAACCTGGCTCATTACTGACGCTGAGGCGCGAAAGCGTGGGGAGCAAACAGGATTAGATACCCTGGTAGTCCACGCTGTAAACGATGTGTGCTAGATGTTGGGTGACTTAGTCATTCAGTGTCGCAGTTAACGCGTTAAGCACACCGCCTGGGGAGTACGGCCGCAAGGTTGAAACTCAAAGGAATTGACGGGGGCCCGCACAAGCGGTGGAGCATGTGGTTTAATTCGAAGCAACGCGCAGAACCTTACCAGGGCTTGAATGTAGAGGCTGCAAGCAGAGATGTTTGTTTCCCGCAAGGGACCTCTAACACAGGTGCTGCATGGCTGTCGTCAGCTCGTGTCGTGAGATGTTGGGTTAAGTCCCGCAACGAGCGCAACCCCTATCTTTAGTTGCCATCAGGTTGGGCTGGGCACTCTAGAGAGACTGCCGGTGACAAGCCGGAGGAAGGTGGGGATGACGTCAAGTCCTCATGGCCCTTATGTCCTGGGCTACACACGTGCTACAATGGCGGTGACAGTGGGAAGCTAGGTGGTGACACCATGCTGATCTCTAAAAGCCGTCTCAGTTCGGATTGCACTCTGCAACTCGAGTGCATGAAGGTGGAATCGCTAGTAATCGCGGATCAGCATGCCGCGGTGAATACGTTCCCGGGCCTTGTACACACCGCCCGTCACACCTTGGGAGTTGGTTTGACCTTAAGCCGGTGAGCGAACCGCAAGGGACGCAGCC |
